# Stereoselective Synthesis of Heavily Hydroxylated Azepane Iminosugars via Osmium-Catalyzed Tethered Aminohydroxylation

**DOI:** 10.1021/acs.orglett.3c02087

**Published:** 2023-07-29

**Authors:** Macarena Martínez-Bailén, Camilla Matassini, Francesca Clemente, Cristina Faggi, Andrea Goti, Francesca Cardona

**Affiliations:** Dipartimento di Chimica “Ugo Schiff” (DICUS), Università di Firenze, Via della Lastruccia 3-13, 50019 Sesto Fiorentino, FI, Italy

## Abstract

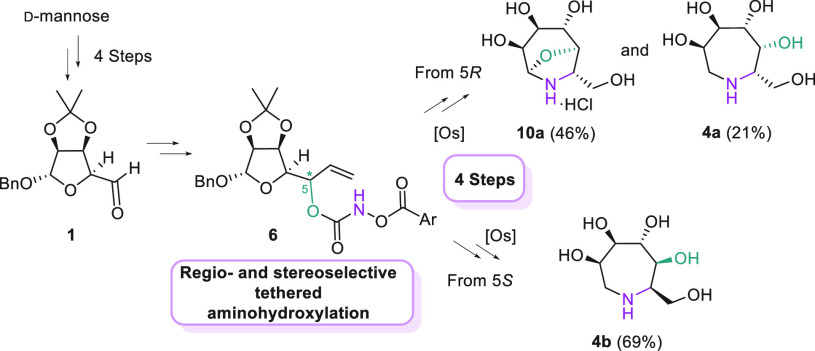

A novel stereoselective synthetic approach to pentahydroxyazepane iminosugars is described. The strategy relies on a key osmium-catalyzed aminohydroxylation reaction of allylic alcohols obtained via addition of vinylmagnesium bromide to a d-mannose-derived aldehyde, which forms the new C–N bond with complete regio- and stereocontrol according to the tethering approach. Subsequent intramolecular reductive amination afforded the desired azepanes. This method represents the first application of the osmium-catalyzed tethered aminohydroxylation reaction to the synthesis of iminosugars.

Iminosugars are glycomimetics with a basic nitrogen atom replacing the endocyclic oxygen in carbohydrates.^1^ As structural analogues of sugars, they offer the opportunity to mimic the biological action of carbohydrates while circumventing their drawbacks, thereby representing valuable and promising tools in medicinal chemistry, mainly due to their ability to inhibit glycosidases and glycosyltransferases.^2−4^ Most of the research has been focused on the synthesis of five- and six-membered iminosugars (polyhydroxylated pyrrolidines and piperidines, respectively).^5^ Polyhydroxyazepanes, their seven-membered ring analogues, also named polyhydroxyperhydroazepines or seven-membered iminocyclitols, have been investigated much less frequently. In 1996, Wong and co-workers reported that these compounds also inhibit a wide range of glycosidases.^6−8^ Polyhydroxyazepanes have several properties that make them potentially useful as drug candidates, such as the higher flexibility of their ring in the interaction with the biological target^9^ and the possibility of installing more functional groups, which may improve the bioavailability and selectivity toward a specific enzyme.^10^

From a synthetic point of view, the main challenges are the cyclization to the seven-membered ring and the control of the configuration at the stereocenters. Approaches for accessing polyhydroxyazepanes have been (i) the regioselective ring opening of carbohydrate-derived bis-epoxides by ammonia or primary amines developed by Wong,^7,8^ Lohray,^11^ and Depezay^12^ (Scheme 1a), (ii) the tandem Staudinger-aza Wittig-mediated ring expansion from 6-azido sugars reported by Blériot^13^ (Scheme 1b), (iii) the double reductive amination (DRA) of sugar-derived dialdehydes, recently applied by Shih and Lin for the synthesis of trihydroxyazepanes^14^ (Scheme 1c), and (iv) the ring-closing metathesis (RCM) of diene precursors^15^ (Scheme 1d). Only a few approaches have addressed the stereoselective installation of the C–N bond. Sinaÿ and Blériot reported in 2004 the first examples of seven-membered iminoalditols with an extra hydroxymethyl substituent, where the formation of the new C–N bond through reductive amination (RA) occurred with low selectivity^15^ (Scheme 1d). This strategy was subsequently improved by introducing the amine moiety through nucleophilic substitution.^16^ An effective synthesis of a pentahydroxylated azepane was accomplished by means of nitrogen nucleophilic ring opening of a cyclic sulfate derived from d-glucose after asymmetric hydroxylation, and the final cyclization by RA was reported by Dhavale and co-workers^17^ (Scheme 1e). More recently, an indirect method for the stereoselective formation of the C–N bond was pursued by Yu and co-workers^18^ and Désiré, Blériot, and co-workers,^19,20^ who exploited the stereoselective organometallic addition to sugar-derived azepane nitrones (Scheme 1f) and a bicyclic *N*,*O*-acetal, respectively (Scheme 1g).

**Scheme 1:**
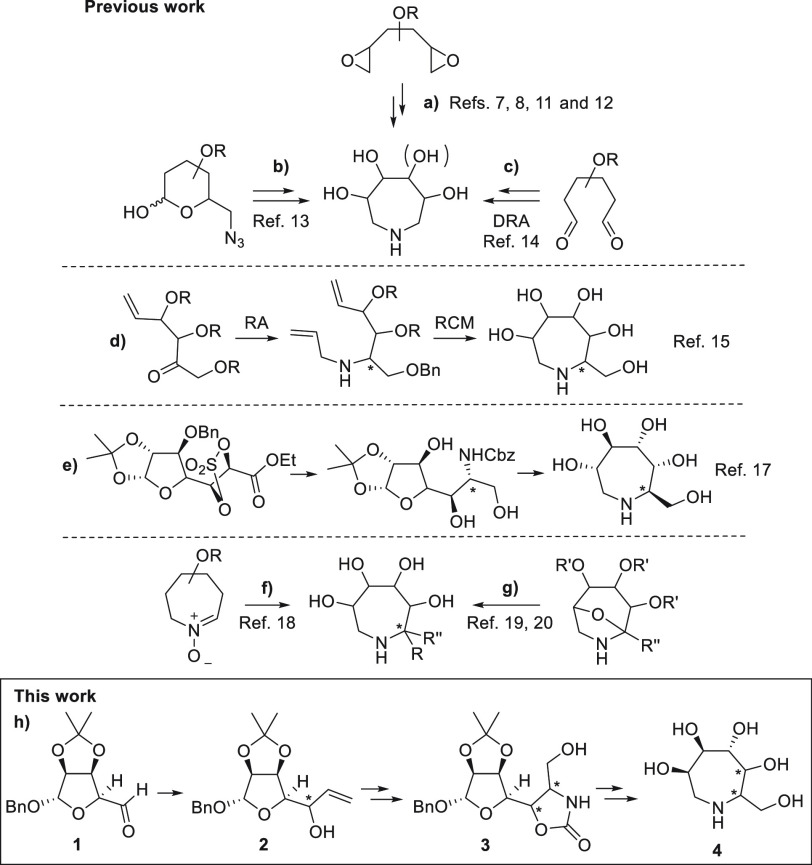
Previous Synthetic Approaches to Polyhydroxylated Azepanes and the Aim of This Work

We envisaged that an osmium-catalyzed Sharpless *syn*-aminohydroxylation of olefins^21^ would have accomplished the desired stereoselective formation of the new C–N bond when the reaction is carried out on appropriate allylic alcohols according to the tethering strategy developed by Donohoe in 2001 [osmium-catalyzed tethered aminohydroxylation (TA)], which would also guarantee full regiocontrol.^22^ Some of us have recently exploited this approach for the synthesis of 2- and 3-aminosugars from glycals and other unsaturated carbohydrates.^23,24^ The required allyl alcohol **2** would be obtained via addition of vinylmagnesium bromide to d-mannose-derived aldehyde **1**,^25^ which installs the first new stereocenter. Conversion of **2** into the required aroyloxycarbamate and accomplishment of the key TA would lead to oxazolidinone **3** with control of the configuration at the new C–N bond. Final deprotections and RA would give the desired pentahydroxylated azepane **4** (Scheme 1h). We disclose herein the results of this synthetic plan.

The addition of vinylmagnesium bromide to aldehyde **1**, as previously reported,^26^ proceeded with good yields (79%) but afforded epimeric allylic alcohols **2a** and **2b** with no significant stereoselectivity (Table 1, entry 1). Decreasing the reaction temperature resulted in a better diastereoselectivity in favor of **2a** (dr 2.3) but with a lower overall yield of 54% (Table 1, entry 2). Addition of a Lewis acid (1.2 equiv) was then tested, leading to a slight improvement in the diastereoselectivity to 3:1 in favor of **2a** and a satisfactory 69% overall yield when BF_3_·Et_2_O was employed (Table 1, entry 4). The diastereoselectivity of the reaction in favor of isomer **2a** with the *R* configuration at C-5 was confirmed by crystallographic analysis of minor diastereoisomer **2b**, which resulted in being *S*-configured at C-5 [CCDC 2265730 (see Figure S2)]. This outcome is in agreement with previous research by our group.^26^

**Table 1:**
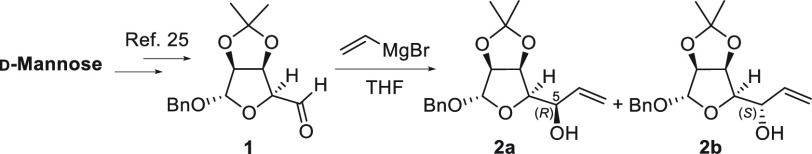
Addition of Vinylmagnesium Bromide (1.8 equiv) to Aldehyde **1**

entry	Lewis acid (equiv)	temp (°C)	time (h)	**2a**:**2b** ratio^a^	yield (%)^b^
1^c^	none	0	2	1.1:1	79
2	none	–78	3	2.3:1	54
3	ZnCl_2_ (1.2)	0	3	2.5:1	51
4	BF_3_·Et_2_O (1.2)	–78	3	3:1	69

a Determined by integration of the signals in the ^1^H NMR spectra of the crude reaction mixture.

b Determined on the basis of the total amount of alcohol (*R* and *S*) recovered after purification by flash column chromatography (FCC).

c Data from ref (26).

Allylic alcohols **2** were partially separated by flash column chromatography and then used as key intermediates for the preparation of the aroyloxycarbamates required for the following tethered aminohydroxylation. This reaction allows the insertion of the amine moiety with total regio- and stereocontrol thanks to the tethered approach.^22^ We chose to use the third-generation protocol for TA (vide infra), which proved to be more efficient with other substrates.^22^ The aroyloxycarbamate needed for this reaction (**6a**) was prepared following the sequence of reactions shown in Scheme 2, which were optimized for major diastereoisomer **2a**.

**Scheme 2:**
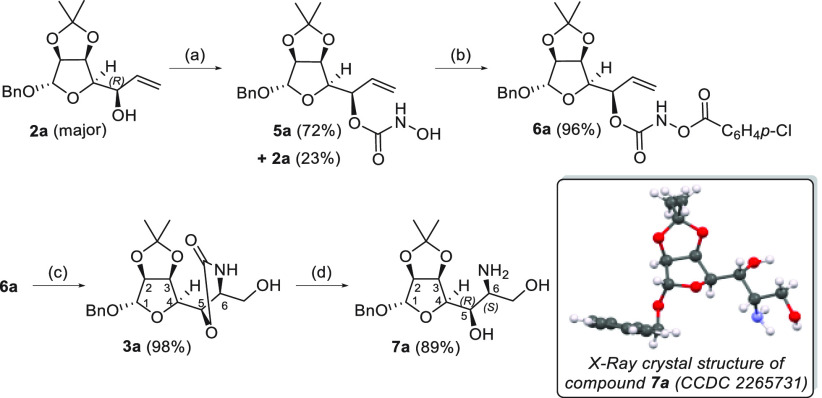
Synthesis of Aroyloxycarbamate **6a**, Osmium-Catalyzed TA, and Oxazolidinone Hydrolysis to Obtain Amino Derivative **7a** Reaction conditions: (a) (1) CDI, toluene, 60 °C, 2 h; (2) NH_2_OH·HCl, Py/toluene, 60 °C, 3.5 h; (b) *p*-ClC_6_H_4_COCl, Et_3_N, CH_2_Cl_2_, −30 °C, 2 h; (c) K_2_OsO_2_(OH)_4_ (3 mol %), 3:1 ^*t*^BuOH/H_2_O, 35–40 °C, 15 h; (d) 2 M NaOH/EtOH (1:1), microwave, 1 h, 90 °C.

Reaction of *R*-configured allylic alcohol **2a** with 1,1-carbonyldiimidazole (CDI) followed by treatment with hydroxylamine in toluene at 60 °C afforded hydroxycarbamate **5a** in good yield (72%, with 23% recovery of the starting material). Treatment with *p*-chlorobenzoyl chloride afforded the desired *O*-aroyloxycarbamate **6a** in excellent yield (96%). Reaction of **6a** in the presence of catalytic K_2_OsO_2_(OH)_4_ (3 mol %) in a 3:1 ^*t*^BuOH/H_2_O solvent at 35–40 °C for 15 h afforded oxazolidinone **3a** in excellent yield (98%) (Scheme 2). The configuration of the new stereogenic center at C-6 was assigned on the basis of one-dimensional (1D) NOE correlation peaks between protons H-1 and H-6 (see the Supporting Information for 1D NOESY spectra) and further confirmed after the next step through X-ray analysis.

Hydrolysis of the oxazolidinone ring with LiOH under conventional heating (80 °C) furnished the desired amine **7a** in good yield (81%) but required long reaction times (>24 h). The use of 2 M aqueous NaOH/EtOH (1:1) under microwave irradiation allowed us to obtain **7a** in a better yield (89%) after just 1 h (Scheme 2). Single-crystal X-ray analysis of **7a** established the *S* configuration at C-6 (Scheme 2), confirming the *syn*-stereoselectivity of the previous TA reaction, resulting from a preferred approach that minimizes A^[1,3]^ strain, as previously shown by Donohoe et al.^27^

The formation of the desired azepane ring required debenzylation and intramolecular RA, which were expected to occur in a domino fashion under hydrogenolytic conditions. To our surprise, catalytic hydrogenation of amine **7a** (Table 2) afforded the desired azepane **9a**(28) as a minor reaction product, together with *N*,*O*-acetal **8a** as the major one, regardless of the palladium source used in the reaction (Table 2).

**Table 2:**
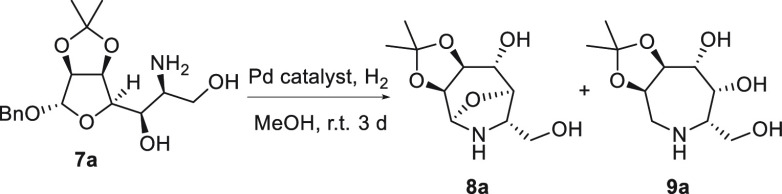
Reaction Yields for Derivatives **8a** and **9a** with Different Palladium Sources

entry	palladium source	yield of **8a** (%)^a^	yield of **9a** (%)^a^
1	Pd(OH)_2_/C	53	27
2	Pd/C with AcOH	49	16

a Isolated yield after purification by flash column chromatography (FCC).

The formation of compound **8a** can be explained on the basis of the mechanism proposed in Scheme 3. According to this mechanism, amine **7a** first undergoes *O*-debenzylation to give hemiacetal **A** in equilibrium with its tautomeric aldehyde **B**. Intramolecular nucleophilic attack of the amine triggers cyclization to seven-membered cyclic hemiaminal **C**, whose presence in the reaction mixture was confirmed by ESI-MS. Acid-catalyzed dehydration of **C** gives intermediate **D** en route to iminium ion **E**, which may undergo deprotonation to form **F** and hydrogenation to azepane **9a** as predicted. Alternatively, intermediate **D** may undergo an intramolecular attack of the hydroxy group at C-5 to afford bicyclic *N*,*O*-acetal **8a**, which may also be obtained from a similar attack at the level of iminium ion **E**. The intramolecular nucleophilic attack of the OH at C-5 in intermediates **D** and **E** is favored by the configuration and geometry of the compounds [as one can infer by the structure of **8a** (see Scheme 4)], with the attack occurring from the opposite face with respect to the vicinal acetonide moiety.

**Scheme 3:**
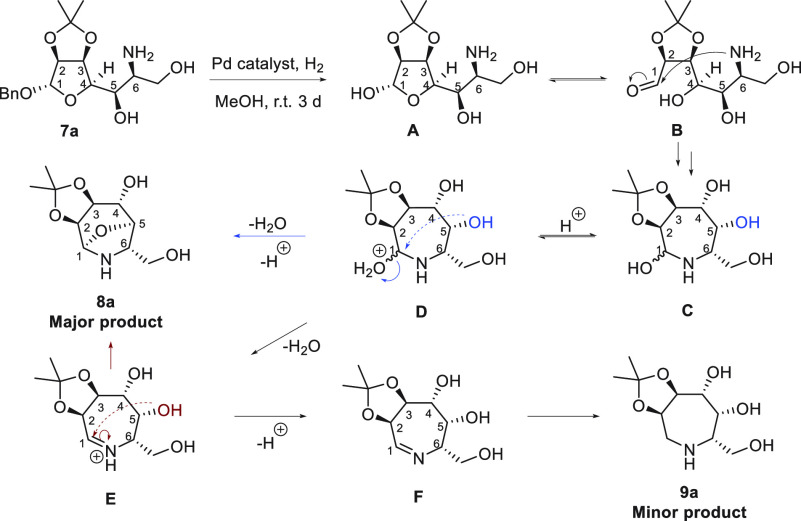
Mechanism Proposed for the Formation of Azepanes **8a** and **9a** The intramolecular attack of the hydroxy group at C-5 on either hemiaminal **D** (blue arrows) or iminium ion **E** (red-purple arrows) would afford bicyclic azepane **8a**.

**Scheme 4:**
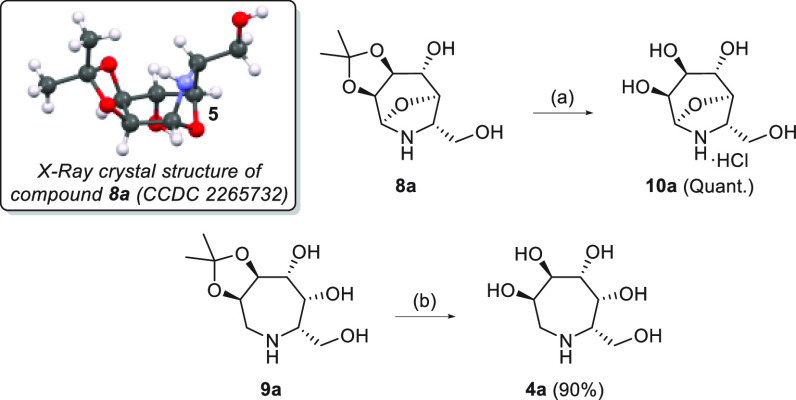
Synthesis of Polyhydroxylated Azepanes **10a** and **4a** Reaction conditions: (a) 1 M HCl/THF (1:1), rt, 1 day; (b) (1) 1 M HCl/THF (1:1), rt, 2 days; (2) Dowex 50WX2.

The structure of compound **8a** was confirmed by two-dimensional (2D) NMR experiments, with an HMBC correlation observed between C-5 and H-1 (Supporting Information), and X-ray crystallography (Scheme 4). The *R* configuration at C-5 clearly favors the intramolecular attack that affords derivative **8a** (Scheme 4). Both compounds **8a** and **9a** were deprotected in acidic media, and the final tetrahydroxylated 8-oxa-6-azabicyclo[3.2.1]octane **10a** and the known tetrahydroxylated azepane **4a**(28) were obtained in excellent yields (Scheme 4).

The same synthetic strategy was applied to the 5*S*-configured minor diastereoisomer (**2b**) obtained from the addition of the vinyl Grignard reagent to aldehyde **1** (Table 1). The corresponding (*S*)-aroyloxycarbamate **6b** was obtained using the same set of reactions and with comparable reaction yields (Scheme 5). Osmium-catalyzed TA gave the corresponding diastereoisomer (**3b**) in excellent yield with the opposite *R* configuration at C-6 installed during formation of the new C–N bond, as confirmed by NOE correlation between proton H-6 and protons H-3 and H-4 (Scheme 5; see the Supporting Information for 1D NOESY spectra). Hydrolytic ring opening of oxazolidinone **3b** occurred promptly under microwave conditions as described above to give the desired amine **7b** (96%) as a precursor of the azepane ring. It was expected that with this substrate, intramolecular attack of OH at C-5 to C-1 would be hampered by steric factors, due to the presence of the acetonide ring on the same face of the azepane, thus preventing the formation of the bicyclic product. Indeed, the hydrogenation of amine **7b** afforded azepane **9b** as a single compound in very good yield (80%) (Scheme 5), which was deprotected to the final pentahydroxyazepane **4b** (96%), whose enantiomer was previously obtained as a hydrochloride salt through a different strategy (Scheme 1d).^15^ The overall yields of polyhydroxyazepanes **4** suffer from the scarce selectivity of the addition step, which, however, offers the opportunity to achieve structural and stereochemical diversity that is useful for biological screening purposes.

**Scheme 5:**
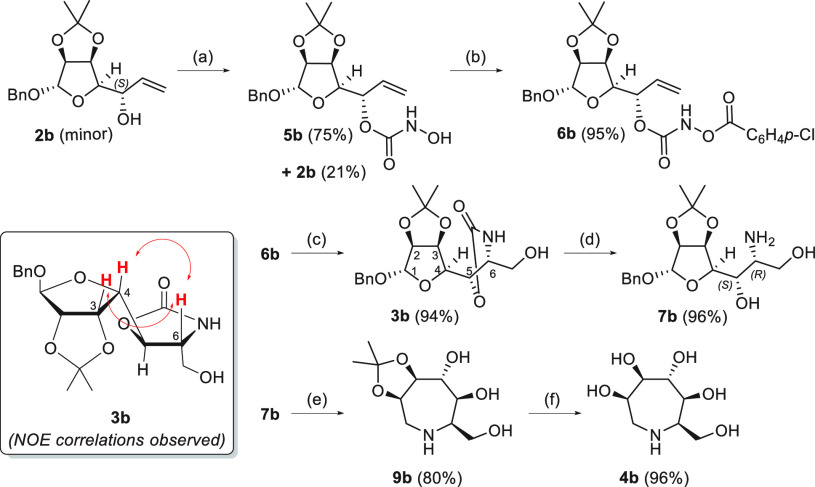
Synthesis of Polyhydroxylated Azepane **4b** Reaction conditions: (a) (1) CDI, toluene, 60 °C, 2 h; (2) NH_2_OH·HCl, Py/toluene, 60 °C, 4.5 h; (b) *p*-ClC_6_H_4_COCl, Et_3_N, CH_2_Cl_2_, −30 °C, 2 h; (c) K_2_OsO_2_(OH)_4_ (3 mol %), 3:1 ^*t*^BuOH/H_2_O, 35–40 °C, 15 h; (d) 2 M NaOH/EtOH (1:1), microwave, 1 h, 90 °C; (e) Pd(OH)_2_/C, H_2_, MeOH, rt, 1.5 days; (f) (1) 1 M HCl/THF (1:1), rt, 2 days; (2) Dowex 50WX2.

Polyhydroxylated azepane iminosugars **4a**, **4b**, and **10a** were tested with respect to a panel of 12 human lysosomal glycosidases [namely α- and β-mannosidase, α- and β-galactosidase, α- and β-glucosidase, α-*N*-acetylgalactosaminidase, α-iduronidase, α-fucosidase, galactosamine (*N*-acetyl)-6-sulfatase, β-hexosaminidase A (Hex A), and β-hexosaminidases (Hex A + Hex B)] due to the importance of these enzymes in lysosomal storage disorders (see the Supporting Information), but scarce inhibition was observed, in analogy with previous results obtained with commercially available glycosidases on **4a**(28) and ***ent*****-4b**.^15^ However, as reported previously, the most promising azepane derivatives display the presence of an alkyl chain on the nitrogen^19^ or are multimerized^29^ to improve the inhibitory potency. As a result, our future work will address these aspects for the development of novel lysosomal glycosidase inhibitors with potential therapeutic effects.

In conclusion, we proved for the first time that the TA reaction on sugar-derived aroyloxycarbamates is a viable methodology for the stereoselective synthesis of iminosugars, as demonstrated in this work for heavily hydroxylated azepane derivatives. Though the final products described herein did not show significant glycosidase inhibition, the synthetic strategy is innovative and could be in principle applied to access iminosugars differing in terms of their configuration, substitution, and even ring size, starting from other carbohydrates and organometal derivatives. Work is underway in our laboratories in this direction.

## Data Availability

The data underlying this study are available in the published article and its Supporting Information.

## Supporting Information Available

Detailed experimental procedures, 1D and 2D NMR spectra of the compounds, data for lysosomal glycosidase inhibition assays and crystallographic data (PDF)
