# Supplementary material for: Stereoselective Synthesis of Heavily Hydroxylated Azepane Iminosugars via Osmium-Catalyzed Tethered Aminohydroxylation
Source: Org Lett. 2023 Jul 29;25(31):5833–7. doi: 10.1021/acs.orglett.3c02087 (PMC10425973; doi:10.1021/acs.orglett.3c02087)
Supplement: Supplementary file 1 — ol3c02087_si_001.pdf [file ol3c02087_si_001.pdf]

SUPPORTING INFORMATION

Stereoselective Synthesis of Heavily Hydroxylated Azepane Iminosugars via Osmium-Catalyzed Tethered Aminohydroxylation

Macarena Martínez-Bailén,*† Camilla Matassini, Francesca Clemente, Cristina Faggi, Andrea Goti and Francesca Cardona*

Dipartimento di Chimica "Ugo Schiff" (DICUS), Università di Firenze, Via della Lastruccia 3-13, 50019 Sesto Fiorentino, FI, Italy

†Present Address: Glycosystems Laboratory, Instituto de Investigaciones Químicas (IIQ), CSIC – Universidad de Sevilla, Av. Américo Vespucio 49, 41092 Seville, Spain

TABLE OF CONTENTS

**1.**	**Experimental Section**	
	1.1. General Methods	S1
	1.2. Detailed experimental procedures	S2-S11
**2.**	**NMR Spectra for compounds (^{1}H, ^{13}C{^{1}H}, gCOSY, gHSQC, gHMBC and 1D NOESY spectra)**	S12-S38
**3.**	**Data for lysosomal glycosidase inhibition assays**	S39-S43
**4.**	**Crystal structure determination for compounds 2b, 7a and 8a**	S44-S49
**5.**	**References**	S50

1. Experimental Section

1.1. General Methods

Commercial reagents were used as received. All reactions were carried out under magnetic stirring and monitored by TLC on 0.25 mm silica gel plates (Merck F254). Column chromatographies were carried out on Silica Gel 60 (32–63 μm) or on silica gel (230–400 mesh, Merck, Darmstadt, Germany). Yields refer to spectroscopically and analytically pure compounds unless otherwise stated. ^{1}H and $^{13}C\{^{1}H\}$ NMR spectra were recorded on a Varian Gemini 200 MHz, a Varian Mercury 400 MHz, or on a Varian INOVA 400 MHz instrument at 25 ºC for solutions in $CDCl_3$, CD_3OD or D_2O. δ are given in ppm and *J* in Hz. *J* are assigned and not repeated. Structural assignments were made with additional information from gCOSY, gHSQC and gHMBC experiments, which were recorded on a Varian Mercury 400 MHz or on a Varian INOVA 400 MHz instrument. 1D NOESY experiments were performed when necessary. The following abbreviations were used to designate multiplicities: s = singlet, d = doublet, t = triplet, q = quartet, m = multiplet, dd = doublet of doublets, td = triplet of doublets, br. = broad. Small-scale microwave-assisted syntheses were carried out in a microwave apparatus for synthesis (CEM Discover, CEM Corporation, North Carolina, US) with a septum-sealed reaction vessel and the reaction mixture temperature is monitored by an infrared temperature sensor. IR spectra were recorded with a Shimadzu IRAffinity-1 or -1S spectrophotometer. ESI-MS spectra were recorded with a Thermo Scientific LCQ fleet ion trap mass spectrometer. Elemental analyses were performed with a Thermoscientific FlashSmart Elemental Analyzer CHNS/O. Optical rotation measurements were performed on a JASCO DIP-370 polarimeter.

1.2. *Detailed experimental procedures*

Synthesis of allylic alcohols 2

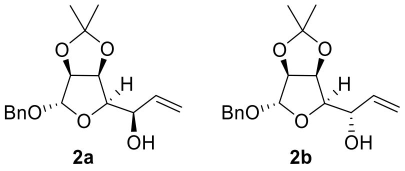

To a solution of **1**[1] (101 mg, 0.363 mmol) in anhydrous THF (5 mL), $BF_3{\cdot}Et_2O$ (56 µL, 0.43 mmol) was added dropwise. After stirring for 20 min at r.t., the solution was cooled at -78 °C and vinylmagnesium bromide (1M in THF, 0.65 mL, 0.65 mmol) was added dropwise and the reaction mixture was stirred at -78 °C for 3 h. After this time, NaOH (1M, 5 mL) was added and the mixture was stirred at r.t. for 10 min. The crude was extracted with CH_2Cl_2 (x3) and the combined organic phases were washed with brine, dried over Na_2SO_4, filtered and concentrated under reduced pressure to give a mixture of **2a**[2] and **2b**[2] (**a**:**b** ratio 3:1, as attested by integrating the signals in the ^{1}H NMR spectrum of the crude, ***Figure S1***). The residue was purified by flash column chromatography on silica gel (EtOAc:Petroleum ether 1:5) to give 29.6 mg (0.0966 mmol, 27%) of **2a**[2] as a colourless oil, 18.2 mg (0.0594 mmol, 16%) of **2b**[2] as a white solid, and 28.9 mg (0.0943 mmol) of a mixture of the two diastereoisomers, impossible to be separated, for a total yield of 69% (76.7 mg, 0.250 mmol).

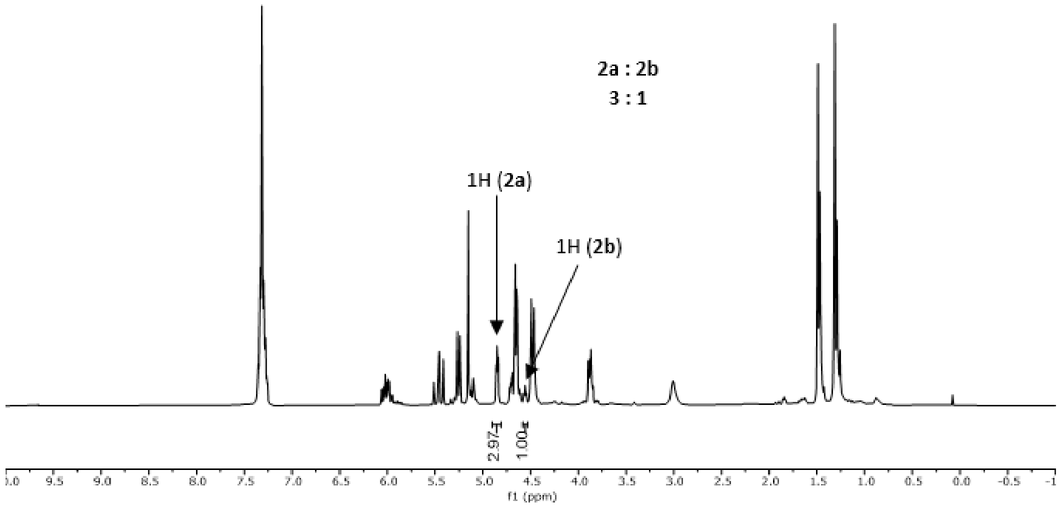

Figure S1. ^{1}H NMR spectrum of the crude reaction mixture ($CDCl_3$, 400 MHz)

Compound **2b** (M.p. = 73.5-75.5 ºC) was crystallized from EtOH to give crystals for X-ray analysis (***Figure S2***, see **section 4** for crystallographic data). ^{1}H NMR data fit with previously reported results in reference 2.

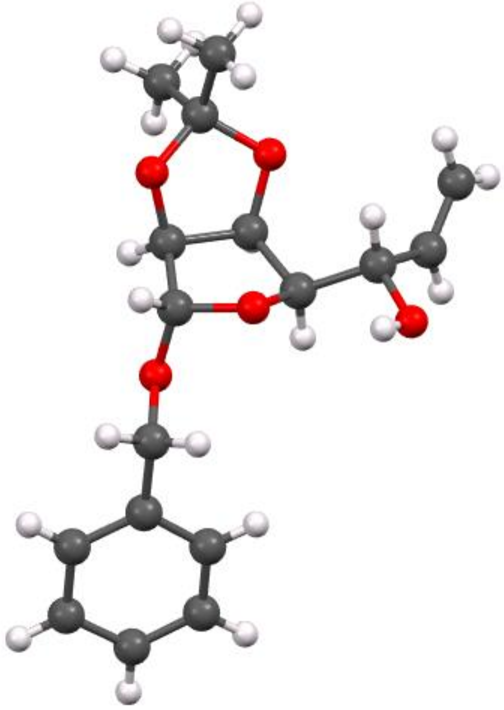

Figure S2. X-Ray crystal structure of minor diastereoisomer **2b** (CCDC 2265730)

Synthesis of hydroxycarbamate 5a

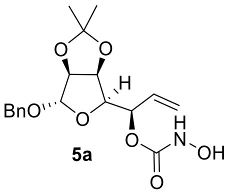

A solution of **2a** (508 mg, 1.66 mmol) in toluene (11.3 mL) was added dropwise to a suspension of 1,1-carbonyldiimidazole (CDI, 746 mg, 4.14 mmol) in toluene (5.7 mL) at 60 ºC (oil bath) and the reaction mixture was further stirred at 60 ºC for 2 h. After this time, the reaction was cooled at room temperature, filtered into a separatory funnel and 70 mL of sat. aq. sol. of NH_4Cl were added. The phases were separated and the aqueous phase was extracted twice with EtOAc. The combined organic layers were washed with brine, dried over Na_2SO_4, filtered, and evaporated to give the corresponding carbonylimidazole derivative which was used in the next step without further purification. A solution of this compound in toluene (11 mL) was added dropwise to a solution of $NH_2OH{\cdot}HCl$ (411 mg, 5.80 mmol) in pyridine (11 mL) at 60 ºC (oil bath). After 3.5

h at 60 ºC, 70 mL of HCl (1M) were added, the phases were separated and the aqueous layer was extracted with EtOAc (x3). The combined organic phases were washed with water and brine (x2), dried over Na_2SO_4, filtered, and concentrated under reduced pressure. The resulting residue was purified by flash column chromatography on silica gel (gradient eluent EtOAc:Petroleum ether from 1:2 to 1:1) to give **5a** (433 mg, 1.19 mmol, 72%, 2 steps, white solid) and **2a** (117 mg, 0.382 mmol, 23%, 2 steps, colourless oil).

Data for compound 5a. M.p. = 129.6-131.4 ºC. $[\alpha]_D^{21}$ + 82.4 (*c* 0.66, $CHCl_3$). IR ($CHCl_3$, ν cm^{-1}) 3546, 3400 (OH, NH), 2941, 1747 (C=O), 1455, 1375, 1209, 1090, 928. ^1H NMR ($CDCl_3$, 400 MHz, δ ppm) δ 7.70 (br. s, 1H, -NH),* 7.36-7.26 (m, 5H, H-Ar), 7.13 (br. s, 1H, -OH),* 5.98-5.90 (m, 1H, H-6), 5.47-5.39 (m, 2H, H-5, H-7a), 5.31 (d, 1H, $J_{7b,6}$ = 10.6 Hz, H-7b), 5.13 (s, 1H, H-1), 4.76-4.73 (m, 1H, H-3), 4.66-4.62 (m, 2H, H-2, -OCH_2Ph-a), 4.48 (d, 1H, $^2J_{H,H}$ = 11.8 Hz, -OCH_2Ph-b), 4.01 (dd, 1H, $J_{4,5}$ = 8.7 Hz, $J_{4,3}$ = 3.6 Hz, H-4), 1.47 (s, 3H, -C(CH_3)$_2$), 1.31 (s, 3H, -C(CH_3)$_2$). $^{13}C\{^1H\}$ NMR ($CDCl_3$, 100 MHz, δ ppm) δ 158.0 (C=O), 137.2 (Cq Ar), 133.9 (C-6), 128.6 (2C, C Ar), 128.1 (2C, C Ar), 128.0 (C Ar), 118.5 (C-7), 113.2 (-*C*(CH_3)$_2$), 105.6 (C-1), 84.9 (C-2), 80.5 (C-4), 79.4 (C-3), 73.1 (C-5), 69.3 (-O*C*H_2Ph), 26.0 (-C(*C*H_3)$_2$), 25.0 (-C(*C*H_3)$_2$). MS (ESI) m/z: $[M + Na]^+$ Calcd for $C_{18}H_{23}NO_7Na$ 388.14; Found 388.07. Anal. Calcd for $C_{18}H_{23}NO_7$: C, 59.17; H, 6.35; N, 3.83. Found: C, 59.34; H, 6.41; N, 3.76.

Synthesis of *O*-aroyloxycarbamate 6a

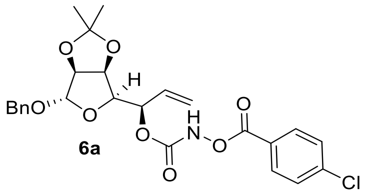

To solution of **5a** (513 mg, 1.40 mmol) in anhydrous CH_2Cl_2 (25 mL) at -30 ºC, Et_3N (198 μL, 1.40 mmol) and *p*-chlorobenzoyl chloride (182 μL, 1.42 mmol) were added and the reaction mixture was stirred at -30 °C for 2 h. After this time, the crude was diluted with CH_2Cl_2 and 40 mL of HCl (1M) were added. The phases were separated and the aqueous layer was extracted with CH_2Cl_2 (x3). The combined organic phases were washed with aq. sat. sol. of $NaHCO_3$ (x2) and brine, dried over Na_2SO_4, filtered and concentrated under reduced pressure. The resulting residue was purified by flash column chromatography on silica gel (gradient eluent EtOAc:Petroleum ether from 1:10 to 1:2) to give **6a** (679 mg, 1.35 mmol, 96%) as a white solid. M.p. = 97.6-98.4 ºC. $[\alpha]_D^{22}$ + 66.5 (*c* 0.56, $CHCl_3$). IR ($CHCl_3$, ν cm^{-1}) 3341 (NH), 2941, 1751 (C=O), 1598 (C=O), 1455, 1375, 1234, 1093, 937. ^1H NMR ($CDCl_3$, 400 MHz, δ ppm) δ 8.55 (s, 1H, NH), 8.02 (d, 2H, $^2J_{H,H}$ = 8.6 Hz, H-Ar), 7.46 (d, 2H, H-Ar), 7.37-7.26 (m, 5H, H-Ar), 6.02-5.93 (m, 1H, H-6), 5.53-5.46 (m, 2H, H-5, H-7a), 5.36 (d, 1H, $J_{7b,6}$ = 10.6 Hz, H-7b), 5.13 (s, 1H,

* Exchangeable signals.

H-1), 4.75-4.72 (m, 1H, H-3), 4.65-4.62 (m, 2H, H-2, -OCH_2Ph-a), 4.47 (d, 1H, $^2J_{H,H}$ = 11.8 Hz, -OCH_2Ph-b), 4.05 (dd, 1H, $J_{4,5}$ = 8.4 Hz, $J_{4,3}$ = 3.6 Hz, H-4), 1.46 (s, 3H, -C(CH_3)$_2$), 1.30 (s, 3H, -C(CH_3)$_2$). ^{13}C{^{1}H} NMR ($CDCl_3$, 100 MHz, δ ppm) δ 165.0 (C=O), 155.2 (C=O), 141.0 (Cq Ar), 137.2 (Cq Ar), 133.4 (C-6), 131.4 (2C, C Ar), 129.3 (2C, C Ar), 128.6 (2C, C Ar), 128.1 (2C, C Ar), 128.0 (C Ar), 125.2 (Cq Ar), 118.9 (C-7), 113.0 (-*C*(CH_3)$_2$), 105.7 (C-1), 84.7 (C-2), 80.4 (C-4), 79.4 (C-3), 74.3 (C-5), 69.3 (-O*C*H_2Ph), 26.0 (-C(*C*H_3)$_2$), 24.9 (-C(*C*H_3)$_2$). MS (ESI) m/z: [M + Na]$^+$ Calcd for $C_{25}H_{26}ClNO_8Na$ 526.12; Found 526.01. Anal. Calcd for $C_{25}H_{26}ClNO_8$: C, 59.59; H, 5.20; N, 2.78. Found: C, 59.31; H, 5.16; N, 2.69.

Synthesis of oxazolidinone 3a

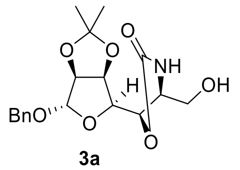

3a

To a solution of **6a** (664 mg, 1.32 mmol) in ^{t}BuOH:H_2O 3:1 (total volume 36 mL), $K_2OsO_2(OH)_4$ (14.9 mg, 0.0395 mmol, 3 mol%) was added and the reaction mixture was stirred at 35-40 ºC (oil bath) overnight (15 h). After this time, Na_2SO_3 (267 mg, 2.10 mmol) was added and the solvent was evaporated after the addition of toluene. The resulting residue was dissolved in EtOAc and water, and the phases were separated. The aqueous layer was extracted twice with EtOAc. The combined organic phases were washed with aq. sat. sol. of $NaHCO_3$ and brine, dried over Na_2SO_4, filtered and concentrated under reduced pressure. The resulting residue was purified by flash column chromatography on silica gel (gradient eluent from EtOAc:Petroleum ether 2:1 to EtOAc) to give **3a** (475 mg, 1.30 mmol, 98%) as a whitish solid.

M.p. = 72.9-74.3 ºC. $[\alpha]_D^{22}$ + 12.0 (*c* 0.69, $CHCl_3$). IR ($CHCl_3$, ν cm^{-1}) 3460 (OH, NH), 2941, 1759 (C=O), 1456, 1383, 1234, 1162, 1082. ^{1}H NMR ($CDCl_3$, 400 MHz, δ ppm) δ 7.36-7.26 (m, 5H, H-Ar), 6.85 (br. s, 1H, NH), 5.13 (s, 1H, H-1), 4.81-4.78 (m, 1H, H-3), 4.71 (t, *J* = 5.1 Hz, 1H, H-5), 4.67-4.63 (m, 2H, H-2, -OCH_2Ph-a), 4.49 (d, 1H, $J_{H,H}$ = 11.9 Hz, -OCH_2Ph-b), 4.19-4.17 (m, 1H, H-4), 4.00-3.86 (m, 2H, H-6, OH), 3.76 (dd, 1H, $J_{7a,7b}$ = 11.7 Hz, $J_{7a,6}$ = 2.3 Hz, H-7a), 3.64 (dd, 1H, $J_{7b,6}$ = 4.8 Hz, H-7b), 1.43 (s, 3H, -C(CH_3)$_2$), 1.28 (s, 3H, -C(CH_3)$_2$). ^{13}C{^{1}H} NMR ($CDCl_3$, 50 MHz, δ ppm) δ 160.0 (C=O), 137.2 (Cq Ar), 128.6 (2C, C Ar), 128.1 (2C, C Ar), 128.0 (C Ar), 112.9 (-*C*(CH_3)$_2$), 105.8 (C-1), 85.1 (C-2), 79.7 (C-4), 79.4 (C-3), 76.0 (C-5), 69.5 (-O*C*H_2Ph), 63.3 (C-7), 56.3 (C-6), 25.7 (-C(*C*H_3)$_2$), 24.3 (-C(*C*H_3)$_2$). MS (ESI) m/z: [M + Na]$^+$ Calcd for $C_{18}H_{23}NO_7Na$ 388.14; Found 388.09. Anal. Calcd for $C_{18}H_{23}NO_7$: C, 59.17; H, 6.35; N, 3.83. Found: C, 59.05; H, 6.40; N, 3.57.

Synthesis of amine 7a

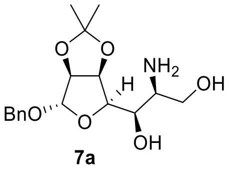

7a

A solution of **3a** (61.2 mg, 0.167 mmol) in 2M NaOH : EtOH (1:1, total volume 2.8 mL) was heated in a MW reactor (90 ºC, 1 h). After evaporation of the solvent, the resulting residue was purified by flash column chromatography on silica gel (CH_2Cl_2:MeOH:NH_4OH 10:1:0.05) to give **7a** (50.5 mg, 0.149 mmol, 89%) as a white solid.

M.p. = 119.4-120.8 ºC. $[\alpha]_D^{22}$ + 78.4 (*c* 1.01, MeOH). IR (neat, ν cm^{-1}) 3355 (OH, NH), 2924, 1586, 1455, 1377, 1206, 1074. ^1H NMR (CD_3OD, 400 MHz, δ ppm) δ 7.36-7.26 (m, 5H, H-Ar), 5.03 (s, 1H, H-1), 4.87-4.85 (m, 1H, H-3), 4.68-4.63 (m, 2H, H-2, -OCH_2Ph-a), 4.53 (d, 1H, $^2J_{H,H}$ = 12.0 Hz, -OCH_2Ph-b), 4.05-3.98 (m, 2H, H-4, H-5), 3.63 (dd, 1H, $J_{7a,7b}$ = 10.7 Hz, $J_{7a,6}$ = 6.1 Hz, H-7a), 3.53 (dd, 1H, $J_{7b,6}$ = 7.0 Hz, H-7b), 2.96-2.93 (m, 1H, H-6), 1.42 (s, 3H, -C(CH_3)$_2$), 1.32 (s, 3H, -C(CH_3)$_2$). $^{13}C\{^1H\}$ NMR (CD_3OD, 50 MHz, δ ppm) δ 139.2 (Cq Ar), 129.4 (2C, C Ar), 129.0 (2C, C Ar), 128.8 (C Ar), 113.5 (-*C*(CH_3)$_2$), 107.3 (C-1), 86.2 (C-2), 81.4 (C-3), 80.0 (C-4 or C-5), 70.2 (-O*C*H_2Ph), 68.9 (C-5 or C-4), 65.0 (C-7), 55.1 (C-6), 26.4 (-C(*C*H_3)$_2$), 25.0 (-C(*C*H_3)$_2$). MS (ESI) m/z: $[M + H]^+$ Calcd for $C_{17}H_{26}NO_6$ 340.18; Found 340.09. Anal. Calcd for $C_{17}H_{25}NO_6$: C, 60.16; H, 7.43; N, 4.13. Found: C, 60.07; H, 7.44; N, 4.04.

Compound **7a** was crystallized from EtOH to give crystals for X-ray analysis (see section 4 for crystallographic data).

Synthesis of polyhydroxylated azepanes 8a and 9a

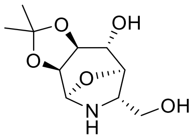

8a

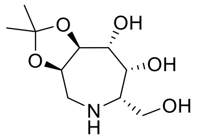

9a

To a solution of **7a** (49.8 mg, 0.147 mmol) in dry MeOH (15 mL), $Pd(OH)_2/C$ (25.3 mg) was added and the reaction mixture was stirred under an atmosphere of hydrogen for 3 d. The mixture was filtered through Celite(R) and the solvent removed under reduced pressure. The resulting residue was purified by flash column chromatography on silica gel (gradient eluent from CH_2Cl_2:MeOH:NH_4OH 7:1:0.05 to 5:1:0.1) to give **8a** (18.0 mg, 0.0778 mmol, 53%, transparent solid) and **9a** (9.3 mg, 0.040 mmol, 27%, white solid).

Data for compound 8a. M.p. = 138.4-139.2 ºC. $[\alpha]_D^{22}$ + 21.6 (*c* 0.59, MeOH). IR ($CHCl_3$, ν cm^{-1}) 3567, 3374 (OH, NH), 2939, 1457, 1384, 1234, 1196, 1053. ^1H NMR (CD_3OD, 400 MHz, δ ppm) δ 4.82-4.81 (m, 1H, H-1), 4.17-4.13 (m, 3H, H-2, H-3, H-5), 3.80 (br. s, 1H, H-4), 3.44 (dd, 1H, J_{-CH_2OH} = 10.3 Hz, $J_{-CH_2OH-a,6}$ = 5.7 Hz, -CH_2OH-a), 3.35 (dd, 1H, $J_{-CH_2OH-b,6}$ = 7.9 Hz, -CH_2OH-

b), 3.29-3.26 (m, 1H, H-6), 1.55 (s, 3H, -C(CH_3)$_2$), 1.32 (s, 3H, -C(CH_3)$_2$). $^{13}C\{^1H\}$ NMR (CD_3OD, 100 MHz, δ ppm) δ 110.1 (-*C*(CH_3)$_2$), 87.9 (C-1), 80.2 (C-5), 77.7, 72.6 (C-2, C-3), 71.3 (C-4), 65.5 (-*C*H_2OH), 58.3 (C-6), 26.6 (-C(*C*H_3)$_2$), 25.0 (-C(*C*H_3)$_2$). MS (ESI) m/z: $[M + Na]^+$ Calcd for $C_{10}H_{17}NO_5Na$ 254.10; Found 254.07. Anal. Calcd for $C_{10}H_{17}NO_5$: C, 51.94; H, 7.41; N, 6.06. Found: C, 52.13; H, 7.49; N, 5.67.

Compound **8a** was crystallized from MeOH to give crystals for X-ray analysis (see **section 4** for crystallographic data).

Data for compound 9a.[3] ^1H NMR (CD_3OD, 400 MHz, δ ppm) δ 4.41 (t, 1H), 4.32-4.26 (m, 1H), 3.89 (br. s, 1H), 3.81 (d, 1H, $J_{H,H}$ = 8.0 Hz), 3.56 (d, 2H, $J_{H,H}$ = 6.7 Hz), 3.03 (dd, 1H, $J_{H,H}$ = 13.7 Hz, $J_{H,H}$ = 4.0 Hz), 2.77 (t, 1H, $J_{H,H}$ = 6.6 Hz), 2.62-2.56 (m, 1H), 1.41 (s, 3H), 1.33 (s, 3H). MS (ESI) m/z: $[M + H]^+$ Calcd for $C_{10}H_{20}NO_5$ 234.13; Found 233.99.

Synthesis of polyhydroxylated azepane 10a

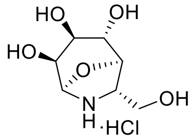

10a

A solution of **8a** (7.6 mg, 0.033 mmol) in 1M HCl : THF (1:1, total volume 1.3 mL), was stirred at r.t. for 1 d. Evaporation of the solvent afforded **10a** (0.033 mmol, quantitative) as a colourless oil.

$[\alpha]_D^{22}$ – 24.1 (*c* 0.86, H_2O). ^1H NMR (D_2O, 400 MHz, δ ppm) δ 5.52 (s, 1H, H-1), 4.53 (br. s, 1H, H-5), 4.45-4.42 (m, 1H, H-6), 4.14-4.07 (m, 3H, H-2, H-3, H-4), 3.94-3.89 (m, 1H, -CH_2OH-a), 3.74-3.68 (m, 1H, -CH_2OH-b). $^{13}C\{^1H\}$ NMR (D_2O, 100 MHz, δ ppm) δ 88.5 (C-1), 78.9 (C-5), 69.7, 69.3, 63.1 (C-2, C-3, C-4), 60.0 (-*C*H_2OH), 58.8 (C-6). MS (ESI) m/z: $[M]^+$ Calcd for $C_7H_{14}NO_5$ 192.09; Found 192.08. Anal. Calcd for $C_7H_{14}ClNO_5$: C, 36.93; H, 6.20; N, 6.15. Found C, 37.11; H, 6.59; N, 6.14.

Synthesis of polyhydroxylated azepane 4a[3]

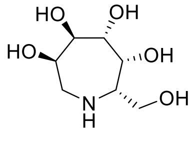

4a

A solution of **9a** (15.1 mg, 0.0647 mmol) in 1M HCl : THF (1:1, total volume 2.6 mL), was stirred at r.t. for 2 d. Evaporation of the solvent and purification through Dowex 50WX2 eluting with MeOH, H_2O and NH_4OH 16.5%, afforded **4a** (11.3 mg, 0.0585 mmol, 90%) as a colourless oil.

^1H NMR (CD_3OD, 400 MHz, δ ppm) δ 4.11-4.08 (m, 1H), 4.00-3.99 (m, 1H), 3.82 (dd, 1H, $J_{H,H}$ = 8.4 Hz, $J_{H,H}$ = 2.5 Hz), 3.72 (dd, 1H, $J_{H,H}$ = 8.4 Hz, $J_{H,H}$ = 2.6 Hz), 3.57 (d, 2H, $J_{H,H}$ = 7.2 Hz),

3.21-3.13 (m, 2H), 2.98 (dd, 1H, $J_{H,H}$ = 14.0 Hz, $J_{H,H}$ = 5.9 Hz). MS (ESI) m/z: $[M + H]^+$ Calcd for $C_7H_{16}NO_5$ 194.10; Found 194.00.

Synthesis of hydroxycarbamate 5b

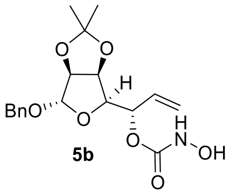

5b

A solution of **2b** (367 mg, 1.20 mmol) in toluene (6 mL) was added dropwise to a suspension of CDI (395 mg, 2.19 mmol) in toluene (3 mL) at 60 ºC (oil bath) and the reaction mixture was further stirred at 60 ºC for 2 h. After this time, the reaction was cooled at room temperature, filtered into a separatory funnel and 50 mL of sat. aq. sol. of NH_4Cl was added. The phases were separated and the aqueous phase was extracted twice with EtOAc. The combined organic layers were washed with brine, dried over Na_2SO_4, filtered and evaporated to give the corresponding carbonylimidazole derivative which was used in the next step without further purification. A solution of this compound in toluene (6 mL) was added dropwise to a solution of $NH_2OH·HCl$ (249 mg, 3.51 mmol) in pyridine (6 mL) at 60 ºC (oil bath). After 4.5 h at 60 ºC, 40 mL of HCl (1M) were added, the phases were separated and the aqueous layer was extracted with EtOAc (x3). The combined organic phases were washed with water and brine (x2), dried over Na_2SO_4, filtered and evaporated. The resulting residue was purified by flash column chromatography on silica gel (gradient eluent EtOAc:Petroleum ether from 1:2 to 1:1) to give **5b** (330 mg, 0.903 mmol, 75%, 2 steps, colourless oil) and **2b** (76.8 mg, 0.251 mmol, 21%, 2 steps, white solid).

Data for compound 5b. $[\alpha]_D^{22}$ + 46.4 (*c* 0.65, $CHCl_3$). IR ($CHCl_3$, ν cm^{-1}) 3547, 3401 (OH, NH), 2941, 1744 (C=O), 1455, 1383, 1234, 1199, 1022. ^1H NMR ($CDCl_3$, 400 MHz, δ ppm) δ 7.49 (br. s, 1H, -NH),* 7.36-7.26 (m, 5H, H-Ar), 6.90 (br. s, 1H, -OH),† 6.03-5.95 (m, 1H, H-6), 5.57-5.54 (m, 1H, H-5), 5.43 (d, 1H, $J_{7a,6}$ = 17.4 Hz, H-7a), 5.30 (d, 1H, $J_{7b,6}$ = 10.8 Hz, H-7b), 5.14 (s, 1H, H-1), 4.68-4.64 (m, 3H, H-2, H-3, -OCH_2Ph-a), 4.49 (d, 1H, $J_{H,H}$ = 11.8 Hz, -OCH_2Ph-b), 4.00 (dd, 1H, $J_{4,5}$ = 8.9 Hz, $J_{4,3}$ = 2.8 Hz, H-4), 1.48 (s, 3H, -C(CH_3)$_2$), 1.29 (s, 3H, -C(CH_3)$_2$). $^{13}C\{^1H\}$ NMR ($CDCl_3$, 50 MHz, δ ppm) δ 158.4 (C=O), 137.4 (Cq Ar), 132.3 (C-6), 128.6 (2C, C Ar), 128.1 (2C, C Ar), 128.0 (C Ar), 118.7 (C-7), 113.1 (-*C*(CH_3)$_2$), 105.9 (C-1), 85.2 (C-2 or C-3), 81.2 (C-4), 79.8 (C-3 or C-2), 74.8 (C-5), 69.3 (-O*C*H_2Ph), 26.2 (-C(*C*H_3)$_2$), 25.0 (-C(*C*H_3)$_2$). MS (ESI) m/z: $[M + Na]^+$ Calcd for $C_{18}H_{23}NO_7Na$ 388.14; Found 388.04. Anal. Calcd for $C_{18}H_{23}NO_7$: C, 59.17; H, 6.35; N, 3.83. Found: C, 59.08; H, 6.52; N, 3.81.

* Exchangeable signals.

Synthesis of *O*-aroyloxycarbamate 6b

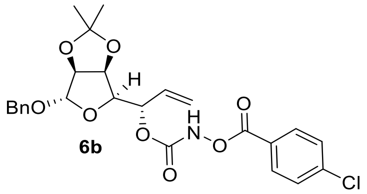

To solution of **5b** (383 mg, 1.05 mmol) in dry CH_2Cl_2 (20 mL) at -30 ºC, Et_3N (150 μL, 1.07 mmol) and *p*-chlorobenzoyl chloride (136 μL, 1.05 mmol) were added and the reaction mixture was stirred at -30 °C for 2 h. After this time, the crude was diluted with CH_2Cl_2 and 30 mL of HCl (1M) were added. The phases were separated and the aqueous layer was extracted with CH_2Cl_2 (x3). The combined organic phases were washed with aq. sat. sol. of $NaHCO_3$ (x2) and brine, dried over Na_2SO_4, filtered and evaporated. The resulting residue was purified by flash column chromatography on silica gel (gradient eluent EtOAc:Petroleum ether from 1:10 to 1:2) to give **6b** (506 mg, 1.00 mmol, 95%) as a white foam.

$[\alpha]_D^{23}$ + 28.3 (*c* 0.92, $CHCl_3$). IR ($CHCl_3$, ν cm^{-1}) 3343 (NH), 2941, 1751 (C=O), 1598 (C=O), 1454, 1361, 1234, 1093, 936. ^1H NMR ($CDCl_3$, 400 MHz, δ ppm) δ 8.46 (s, 1H, NH), 8.04-8.02 (m, 2H, H-Ar), 7.43-7.41 (m, 2H, H-Ar), 7.36-7.26 (m, 5H, H-Ar), 6.06-5.97 (m, 1H, H-6), 5.62-5.59 (m, 1H, H-5), 5.51 (d, 1H, $J_{7a,6}$ = 17.4 Hz, H-7a), 5.35 (d, 1H, $J_{7b,6}$ = 10.8 Hz, H-7b), 5.13 (s, 1H, H-1), 4.68-4.60 (m, 3H, H-2, H-3, -OCH_2Ph-a), 4.45 (d, 1H, $J_{H,H}$ = 11.8 Hz, -OCH_2Ph-b), 4.02-4.00 (m, 1H, H-4), 1.48 (s, 3H, -C(CH_3)$_2$), 1.29 (s, 3H, -C(CH_3)$_2$). $^{13}C\{^1H\}$ NMR ($CDCl_3$, 100 MHz, δ ppm) δ 165.0 (C=O), 155.6 (C=O), 140.9 (Cq Ar), 137.4 (Cq Ar), 131.8 (C-6), 131.4 (2C, C Ar), 129.2 (2C, C Ar), 128.6 (2C, C Ar), 128.1 (2C, C Ar), 127.9 (C Ar), 125.3 (Cq Ar), 119.1 (C-7), 113.1 (-*C*(CH_3)$_2$), 105.7 (C-1), 85.3 (C-2 or C-3), 81.1 (C-4), 79.8 (C-3 or C-2), 75.9 (C-5), 69.1 (-O*C*H_2Ph), 26.2 (-C(*C*H_3)$_2$), 25.1 (-C(*C*H_3)$_2$). MS (ESI) m/z: $[M + Na]^+$ Calcd for $C_{25}H_{26}ClNO_8Na$ 526.12; Found 526.10. Anal. Calcd for $C_{25}H_{26}ClNO_8$: C, 59.59; H, 5.20; N, 2.78. Found: C, 59.60; H, 5.10; N, 2.58.

Synthesis of oxazolidinone 3b

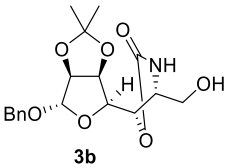

To a solution of **6b** (388 mg, 0.770 mmol) in ^{t}BuOH:H_2O 3:1 (20 mL), $K_2OsO_2(OH)_4$ (8.7 mg, 0.023 mmol, 3 mol%) was added and the reaction mixture was stirred at 35-40 ºC (oil bath) overnight (15 h). After this time, Na_2SO_3 (155 mg, 1.22 mmol) was added and the solvent was evaporated by adding toluene. The resulting residue was dissolved in EtOAc and water and the phases were separated. The aqueous layer was extracted twice with EtOAc. The combined organic phases were washed with aq. sat. sol. of $NaHCO_3$ and brine, dried over Na_2SO_4, filtered and

evaporated. The resulting residue was purified by flash column chromatography on silica gel (gradient eluent from EtOAc to EtOAc:MeOH 30:1) to give **3b** (264 mg, 0.723 mmol, 94%) as a white solid.

M.p. = 161.3-163.6 ºC. $[\alpha]_D^{21}$ + 115.4 (*c* 0.63, MeOH). IR (neat, ν cm^{-1}) 3576 (OH, NH), 2935, 1746 (C=O), 1379, 1237, 1161, 1074. ^{1}H NMR (CD_3OD, 400 MHz, δ ppm) δ 7.39-7.24 (m, 5H, H-Ar), 5.12 (s, 1H, H-1), 4.92-4.90 (m, 1H, H-3), 4.69-4.63 (m, 3H, H-2, H-5, -OCH_2Ph-a), 4.51 (d, 1H, $J_{H,H}$ = 11.7 Hz, -OCH_2Ph-b), 4.17 (dd, 1H, $J_{4,5}$ = 8.6 Hz, $J_{4,3}$ = 3.8 Hz, H-4), 3.81-3.78 (m, 1H, H-6), 3.68 (dd, 1H, $J_{7a,7b}$ = 11.6 Hz, $J_{7a,6}$ = 2.6 Hz, H-7a), 3.59 (dd, 1H, $J_{7b,6}$ = 4.6 Hz, H-7b), 1.44 (s, 3H, -C(CH_3)$_2$), 1.29 (s, 3H, -C(CH_3)$_2$). ^{13}C{^{1}H} NMR (CD_3OD, 100 MHz, δ ppm) δ 161.5 (C=O), 138.7 (Cq Ar), 129.4 (2C, C Ar), 129.3 (2C, C Ar), 128.8 (C Ar), 114.2 (-*C*(CH_3)$_2$), 107.0 (C-1), 86.2 (C-2), 82.6 (C-4), 80.7 (C-3), 79.0 (C-5), 70.0 (-O*C*H_2Ph), 63.8 (C-7), 57.5 (C-6), 26.2 (-C(*C*H_3)$_2$), 24.8 (-C(*C*H_3)$_2$). MS (ESI) m/z: $[M + Na]^+$ Calcd for $C_{18}H_{23}NO_7Na$ 388.14; Found 388.12. Anal. Calcd for $C_{18}H_{23}NO_7$: C, 59.17; H, 6.35; N, 3.83. Found: C, 59.18; H, 6.37; N, 3.67.

Synthesis of amine 7b

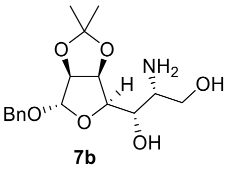

7b

A solution of **3b** (101 mg, 0.277 mmol) in 2M NaOH : EtOH (1:1, total volume 4 mL) was heated in a MW reactor (90 ºC, 1 h). After evaporation of the solvent, the resulting residue was purified by flash column chromatography on silica gel (CH_2Cl_2:MeOH:NH_4OH 10:1:0.05) to give **7b** (90.2 mg, 0.266 mmol, 96%) as a colourless oil.

$[\alpha]_D^{20}$ + 83.2 (*c* 0.82, MeOH). IR (neat, ν cm^{-1}) 3354 (OH, NH), 2937, 1586, 1455, 1374, 1208, 1073. ^{1}H NMR (CD_3OD, 400 MHz, δ ppm) δ 7.36-7.25 (m, 5H, H-Ar), 5.07 (s, 1H, H-1), 4.87-4.85 (m, 1H, H-3), 4.72 (d, 1H, $J_{H,H}$ = 11.7 Hz, -OCH_2Ph-a), 4.65 (d, 1H, $J_{2,3}$ = 5.8 Hz, H-2), 4.49 (d, 1H, -OCH_2Ph-b), 4.19 (dd, 1H, $J_{4,5}$ = 8.0 Hz, $J_{4,3}$ = 3.3 Hz, H-4), 4.01-3.99 (m, 1H, H-5), 3.61 (dd, 1H, $J_{7a,7b}$ = 10.7 Hz, $J_{7a,6}$ = 5.6 Hz, H-7a), 3.52 (dd, 1H, $J_{7b,6}$ = 7.4 Hz, H-7b), 3.01-2.98 (m 1H, H-6), 1.41 (s, 3H, -C(CH_3)$_2$), 1.28 (s, 3H, -C(CH_3)$_2$). ^{13}C{^{1}H} NMR (CD_3OD, 100 MHz, δ ppm) δ 139.0 (Cq Ar), 129.3 (2C, C Ar), 129.1 (2C, C Ar), 128.7 (C Ar), 113.6 (-*C*(CH_3)$_2$), 106.4 (C-1), 86.8 (C-2), 82.5 (C-4), 81.0 (C-3), 70.8 (C-5), 69.7 (-O*C*H_2Ph), 65.8 (C-7), 54.6 (C-6), 26.4 (-C(*C*H_3)$_2$), 24.9 (-C(*C*H_3)$_2$). MS (ESI) m/z: $[M + H]^+$ Calcd for $C_{17}H_{26}NO_6$ 340.18; Found 340.17. Anal. Calcd for $C_{17}H_{25}NO_6$: C, 60.16; H, 7.43; N, 4.13. Found: C, 60.17; H, 7.52; N, 3.90.

Synthesis of polyhydroxylated azepane 4b

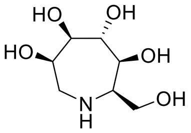

4b

To a solution of **7b** (37.1 mg, 0.109 mmol) in dry MeOH (11 mL), $Pd(OH)_2/C$ (18.6 mg) was added and the reaction mixture was stirred under an atmosphere of hydrogen for 1.5 d. The mixture was filtered through Celite(R) and the solvent removed under reduced pressure. The resulting residue was purified by flash column chromatography on silica gel (CH_2Cl_2:MeOH:NH_4OH 7:1:0.05) to give the corresponding protected azepane **9b** (20.3 mg, 0.0870 mmol, 80%) as a white solid. A solution of this compound (20.3 mg, 0.0870 mmol) in 1M HCl : THF (1:1, total volume 4 mL), was stirred at r.t. for 2 d. Evaporation of the solvent and purification through Dowex 50WX2 eluting with MeOH, H_2O and NH_4OH 16.5%, afforded **4b** (16.2 mg, 0.0839 mmol, 96%) as a colourless oil.

$[\alpha]_D^{22}$ – 27.9 (*c* 0.83, H_2O). ^{1}H NMR (D_2O, 400 MHz, δ ppm) δ 3.94 (br. s, 1H, H-6), 3.83 (dd, 1H, $J_{H,H}$ = 7.3 Hz, $J_{H,H}$ = 2.3 Hz, H-4 or H-5), 3.77-3.72 (m, 2H, H-5 or H-4, H-3), 3.51-3.43 (m, 2H, -CH_2OH), 3.02 (dd, 1H, $J_{7a,7b}$ = 14.4 Hz, $J_{7a,6}$ = 3.8 Hz, H-7a), 2.82-2.79 (m, 2H, H-7b, H-2). ^{13}C{^{1}H} NMR (D_2O, 100 MHz, δ ppm) δ 76.6, 76.3 (C-4, C-5), 72.7 (C-6), 72.0 (C-3), 62.3 (-*C*H_2OH), 57.8 (C-2), 50.4 (C-7). MS (ESI) m/z: $[M + Na]^+$ Calcd for $C_7H_{15}NO_5Na$ 216.08; Found 216.00. Anal. Calcd for $C_7H_{15}NO_5$: C, 43.52; H, 7.83; N, 7.25. Found: C, 43.38; H, 8.13 N, 6.86.

2. NMR Spectra for compounds (^{1}H, ^{13}C{^{1}H}, gCOSY, gHSQC, gHMBC and 1D NOESY spectra)

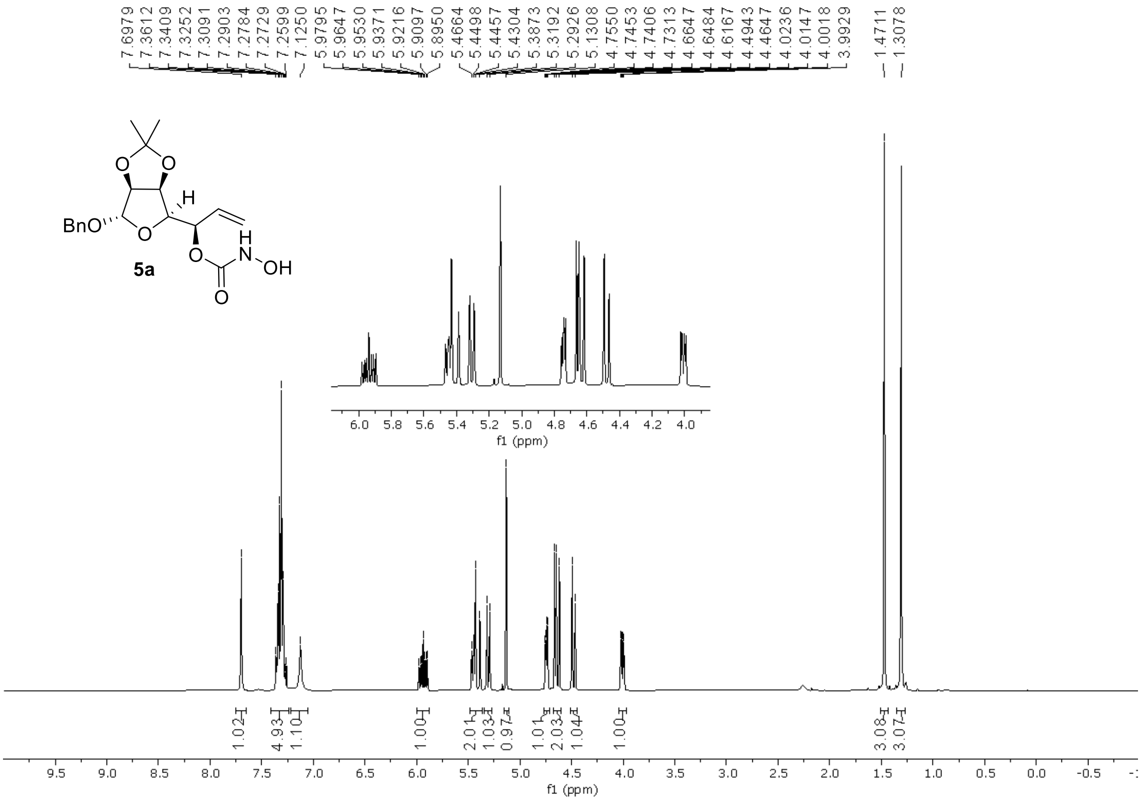

Figure S3. ^{1}H NMR spectrum of compound **5a** ($CDCl_3$, 400 MHz)

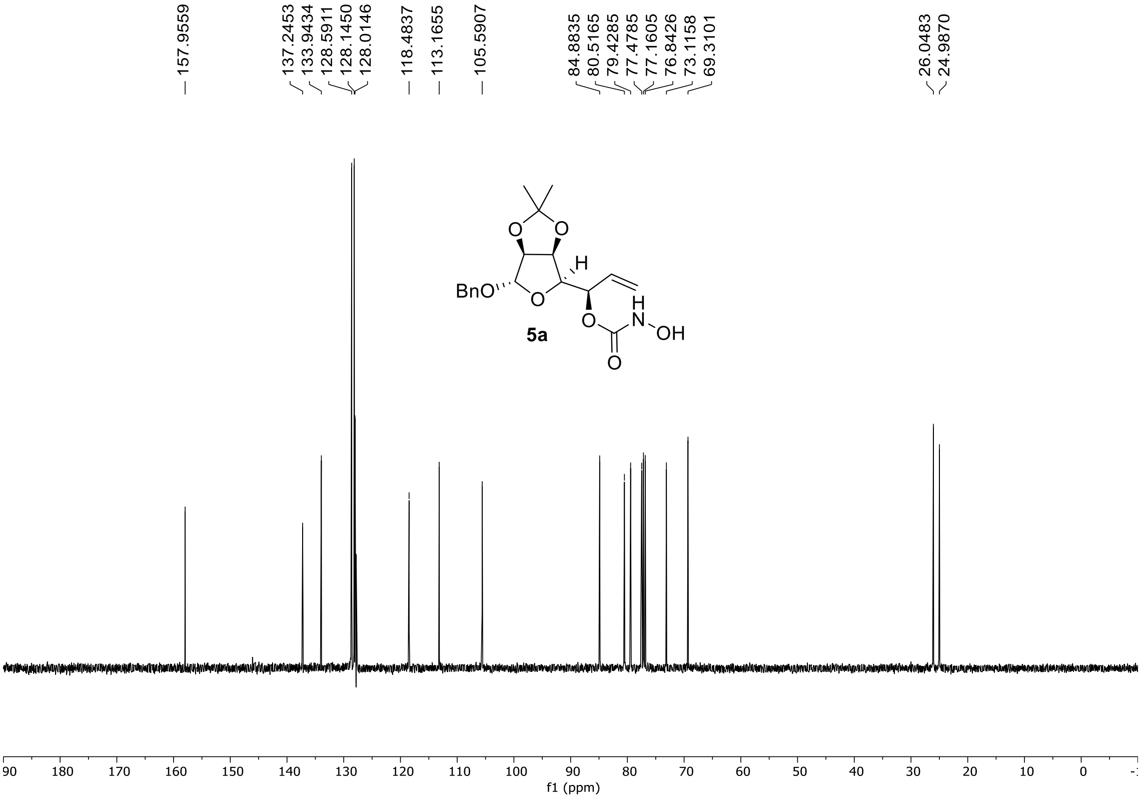

Figure S4. ^{13}C{^{1}H} NMR spectrum of compound **5a** ($CDCl_3$, 100 MHz)

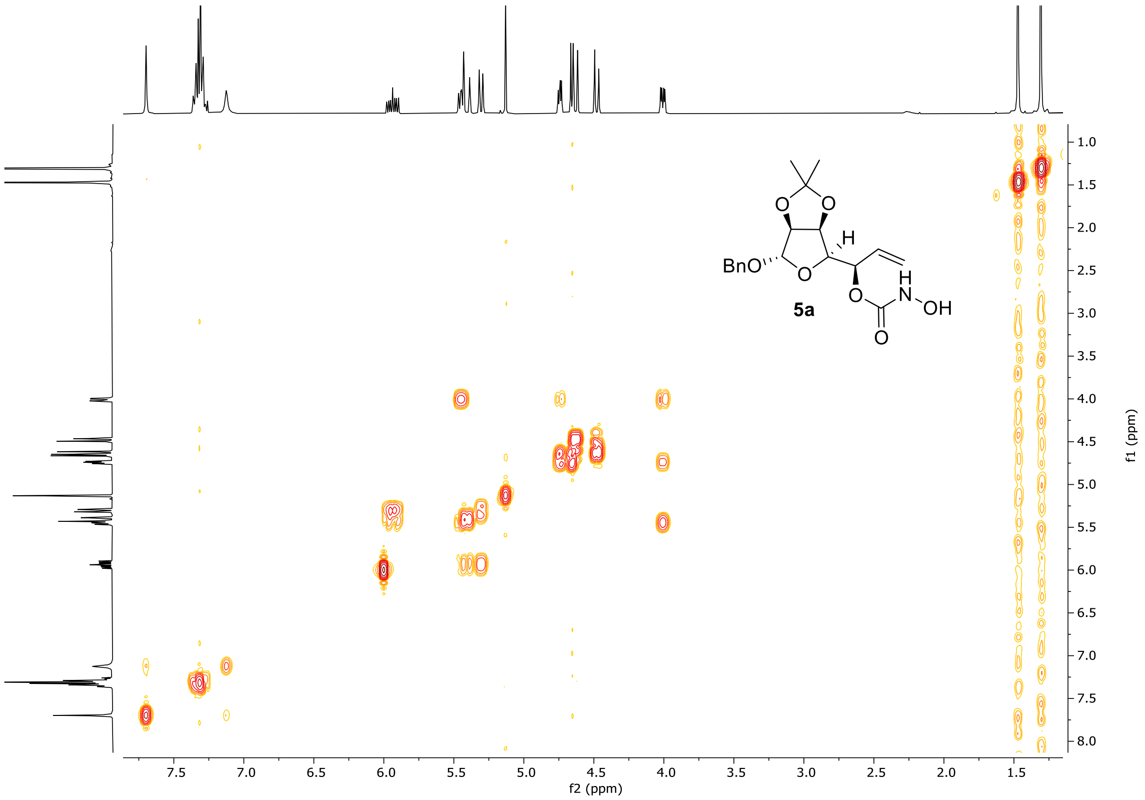

Figure S5. ^{1}H/^{1}H gCOSY spectrum of compound **5a** ($CDCl_3$, 400 MHz)

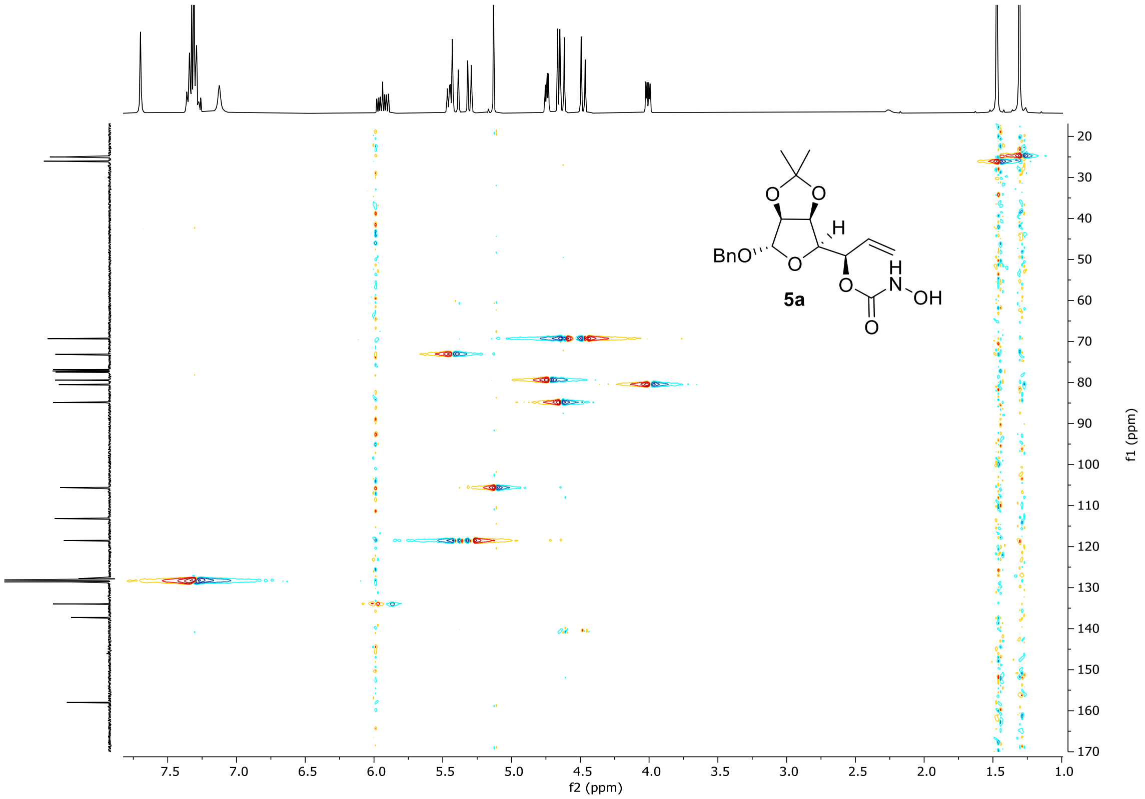

Figure S6. ^{1}H/^{13}C gHSQC spectrum of compound **5a** ($CDCl_3$, 400/100 MHz)

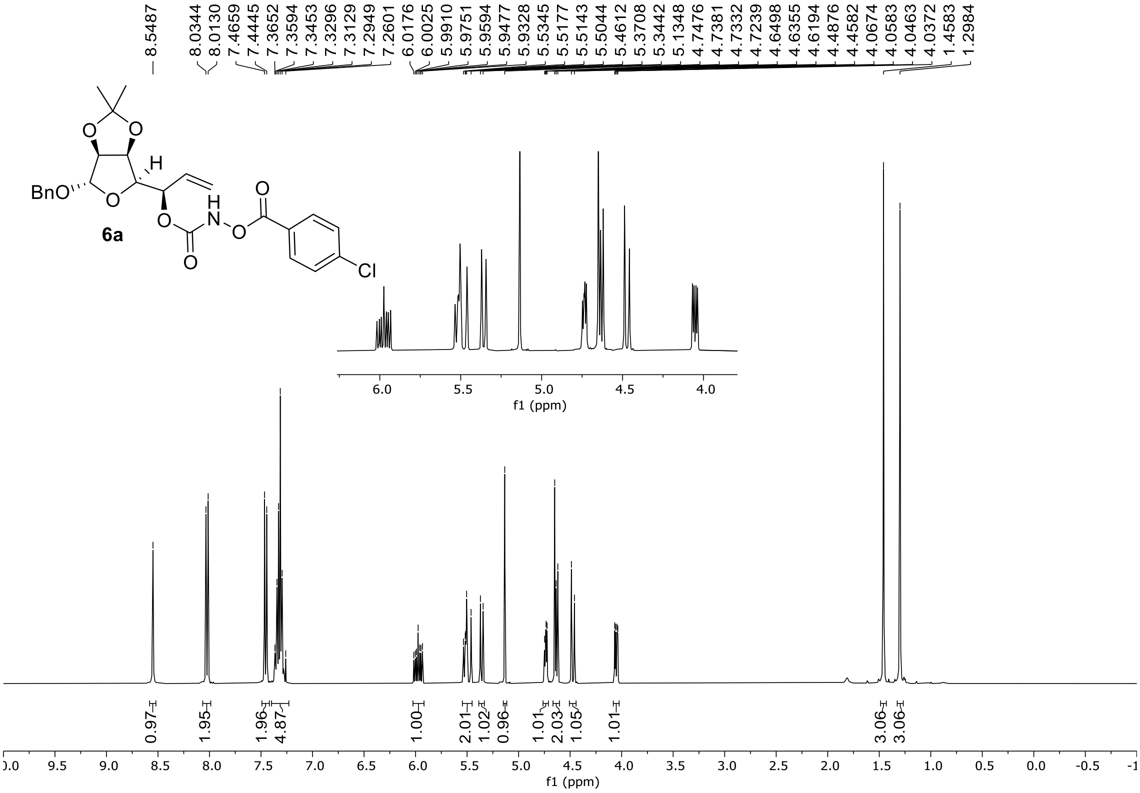

Figure S7. ^{1}H NMR spectrum of compound **6a** ($CDCl_3$, 400 MHz)

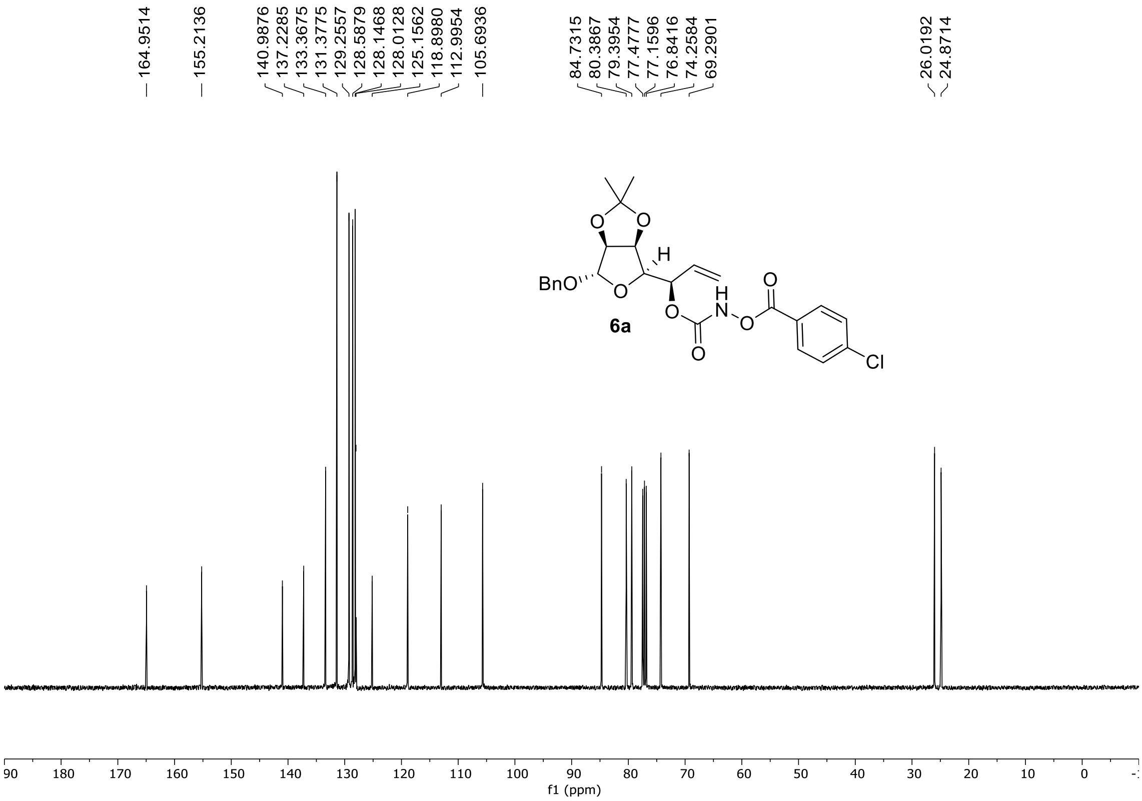

Figure S8. $^{13}C\{^{1}H\}$ NMR spectrum of compound **6a** ($CDCl_3$, 100 MHz)

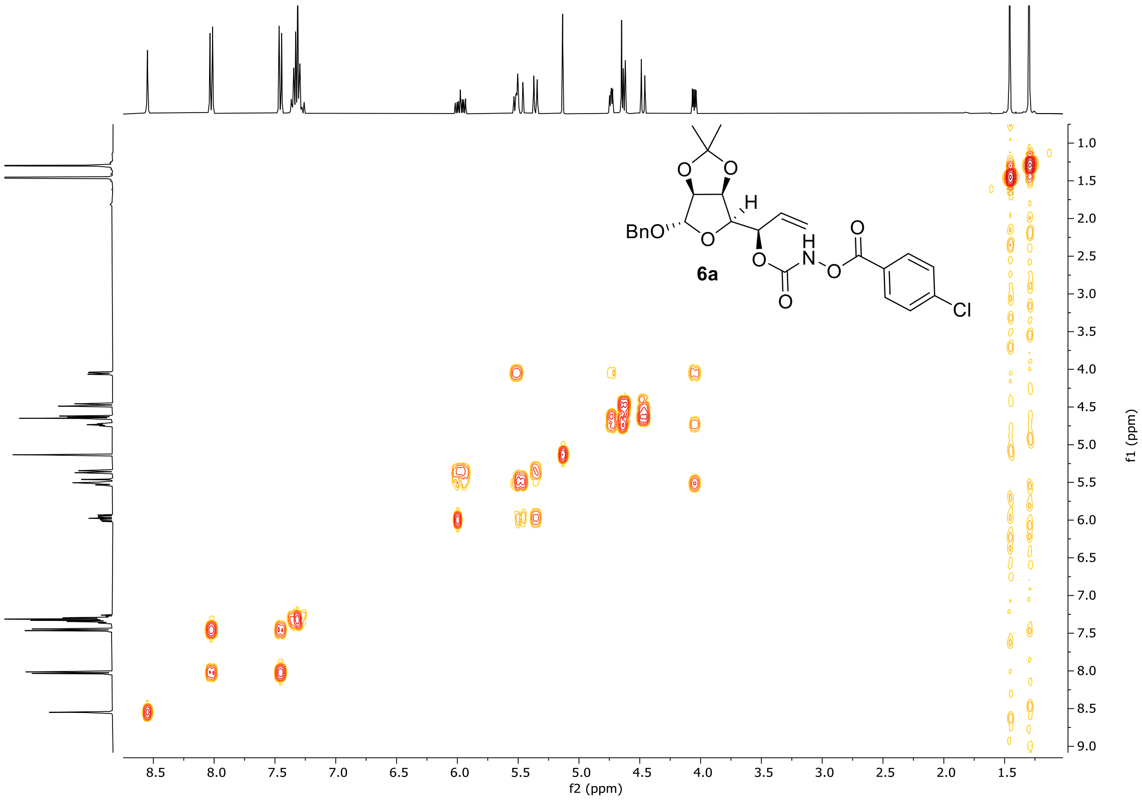

Figure S9. $^{1}H/^{1}H$ gCOSY spectrum of compound **6a** ($CDCl_3$, 400 MHz)

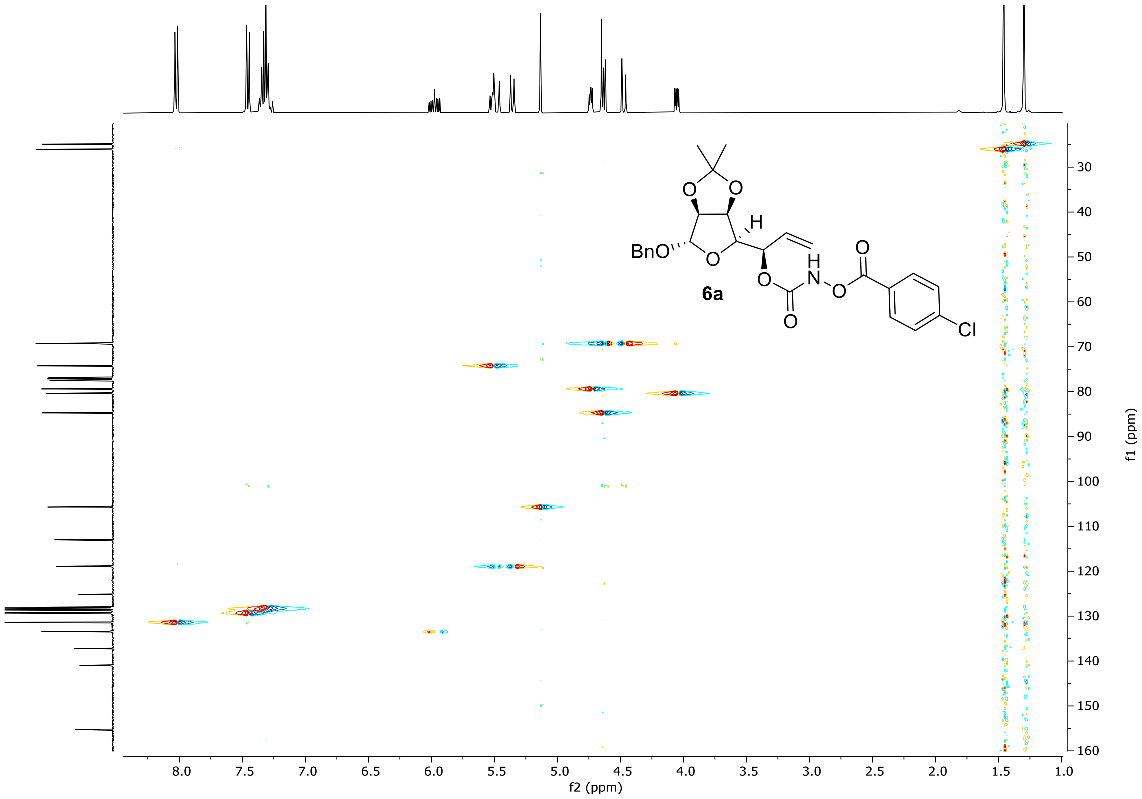

Figure S10. $^{1}H/^{13}C$ gHSQC spectrum of compound **6a** ($CDCl_3$, 400/100 MHz)

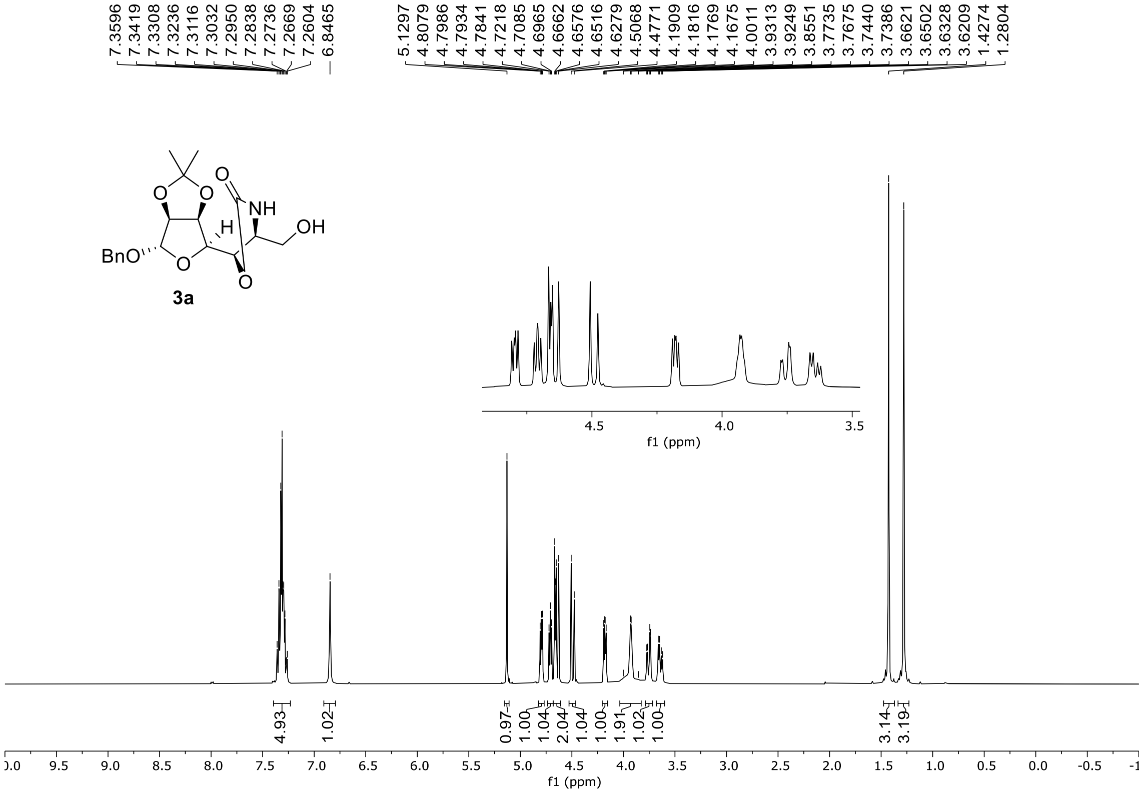

Figure S11. ^{1}H NMR spectrum of compound **3a** ($CDCl_3$, 400 MHz)

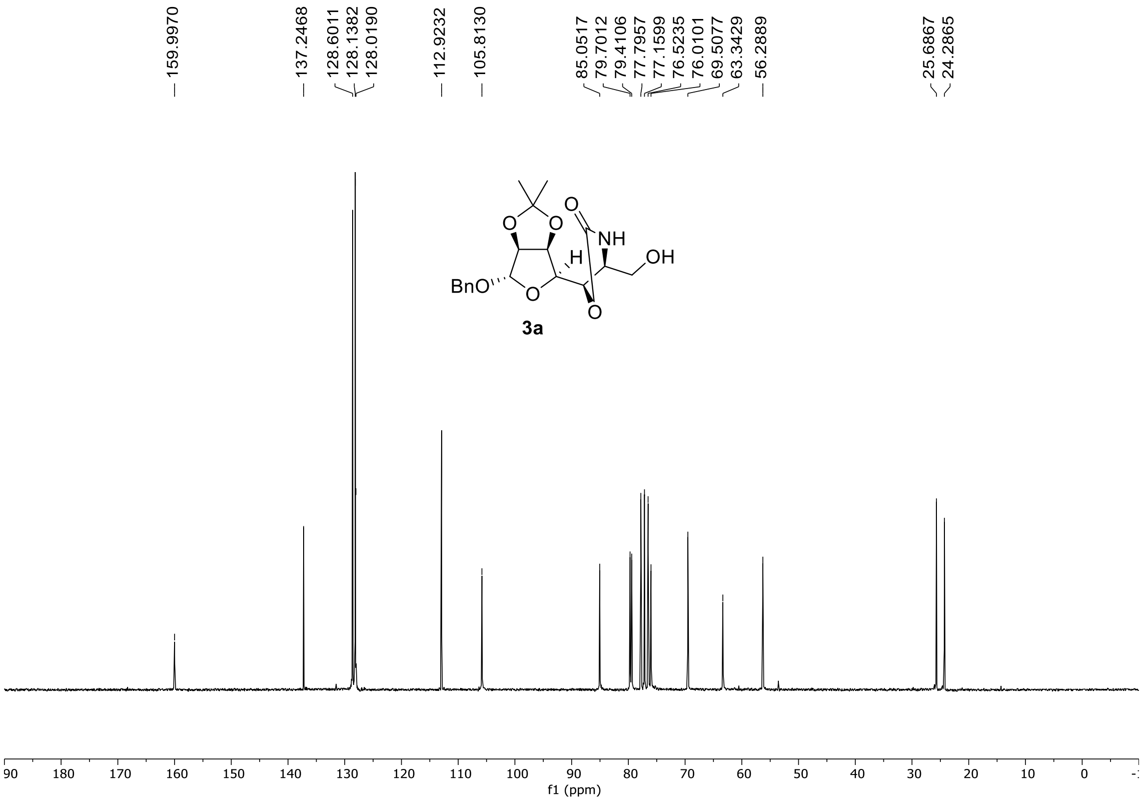

Figure S12. $^{13}C\{^{1}H\}$ NMR spectrum of compound **3a** ($CDCl_3$, 50 MHz)

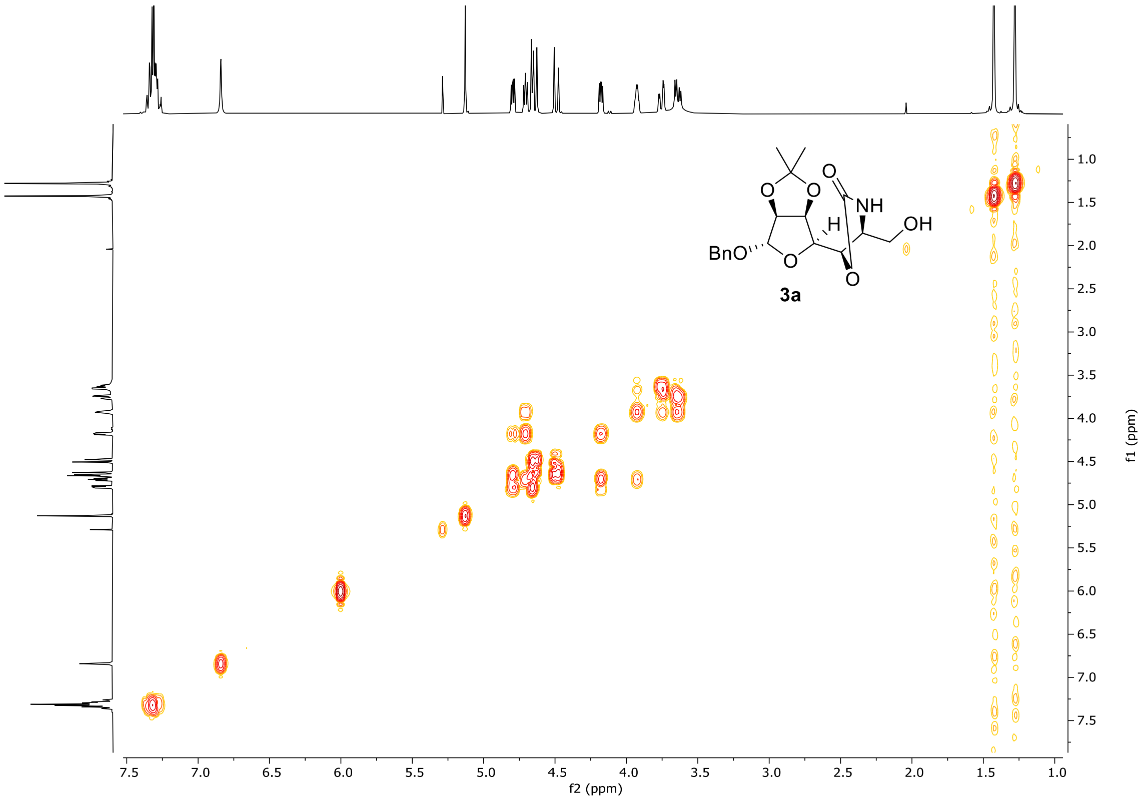

Figure S13. $^{1}H/^{1}H$ gCOSY spectrum of compound **3a** ($CDCl_3$, 400 MHz)

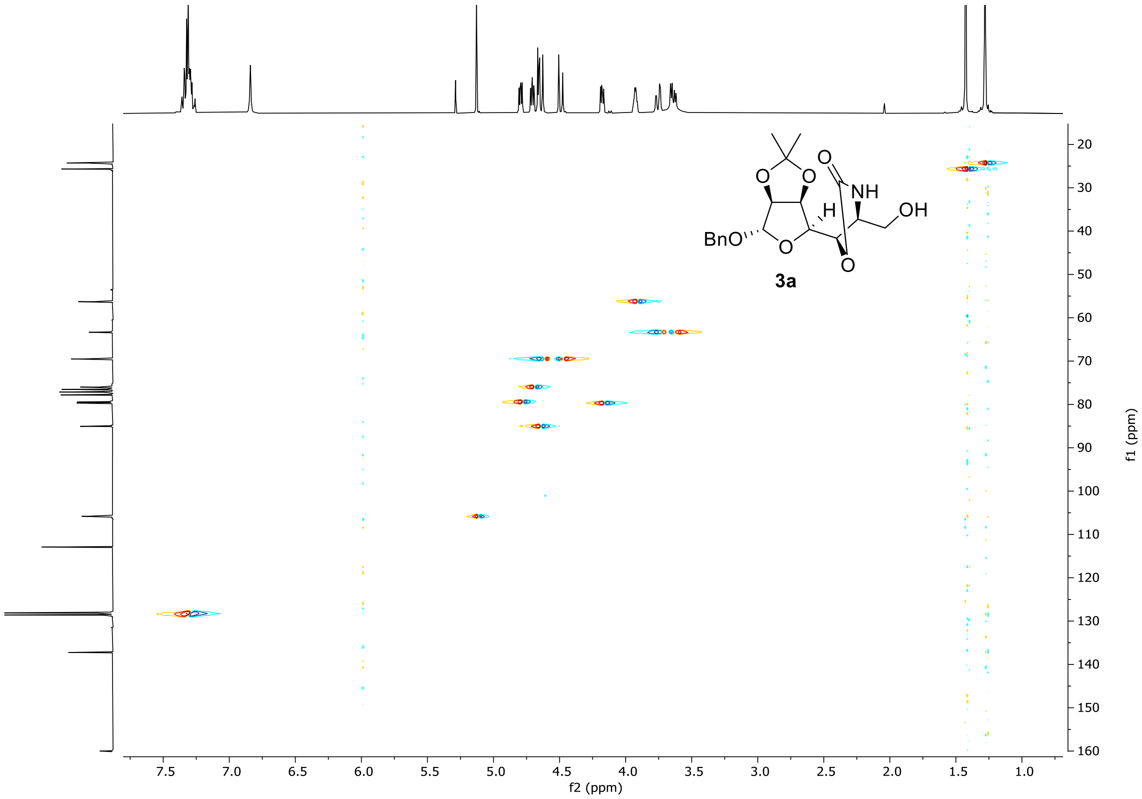

Figure S14. $^{1}H/^{13}C$ gHSQC spectrum of compound **3a** ($CDCl_3$, 400/100 MHz)

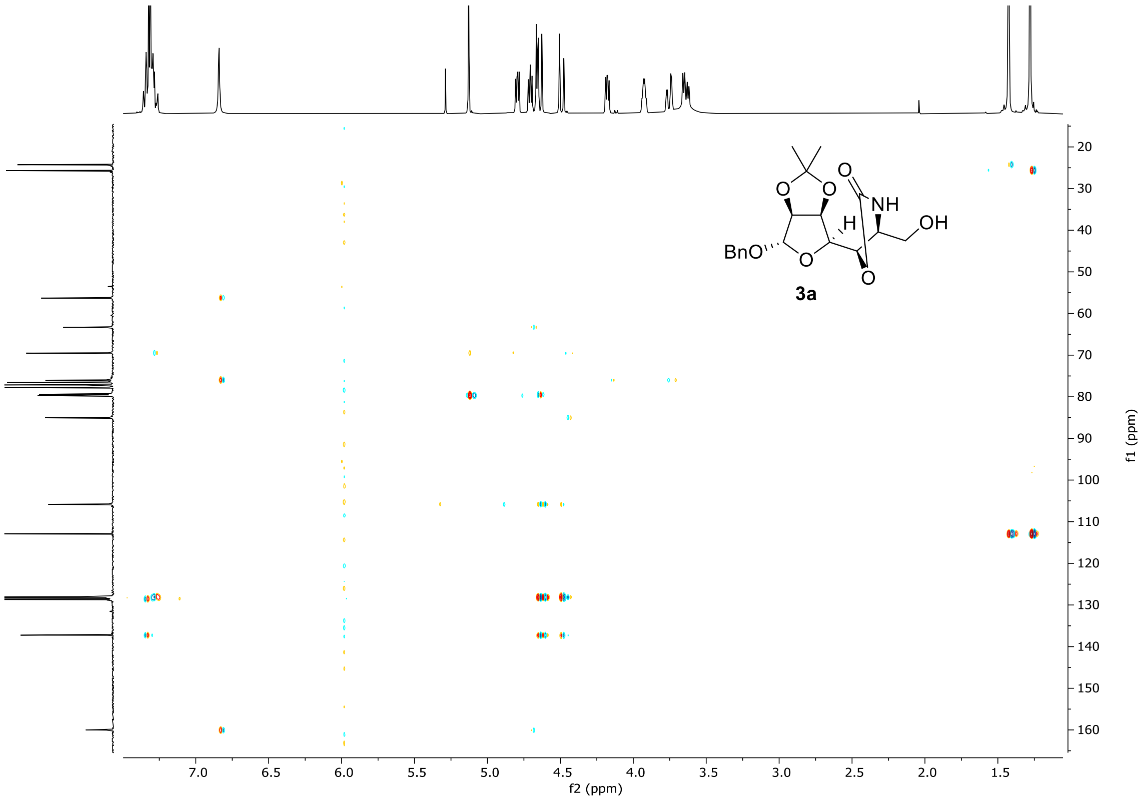

Figure S15. $^1H/^{13}C$ gHMBC spectrum of compound **3a** ($CDCl_3$, 400/100 MHz)

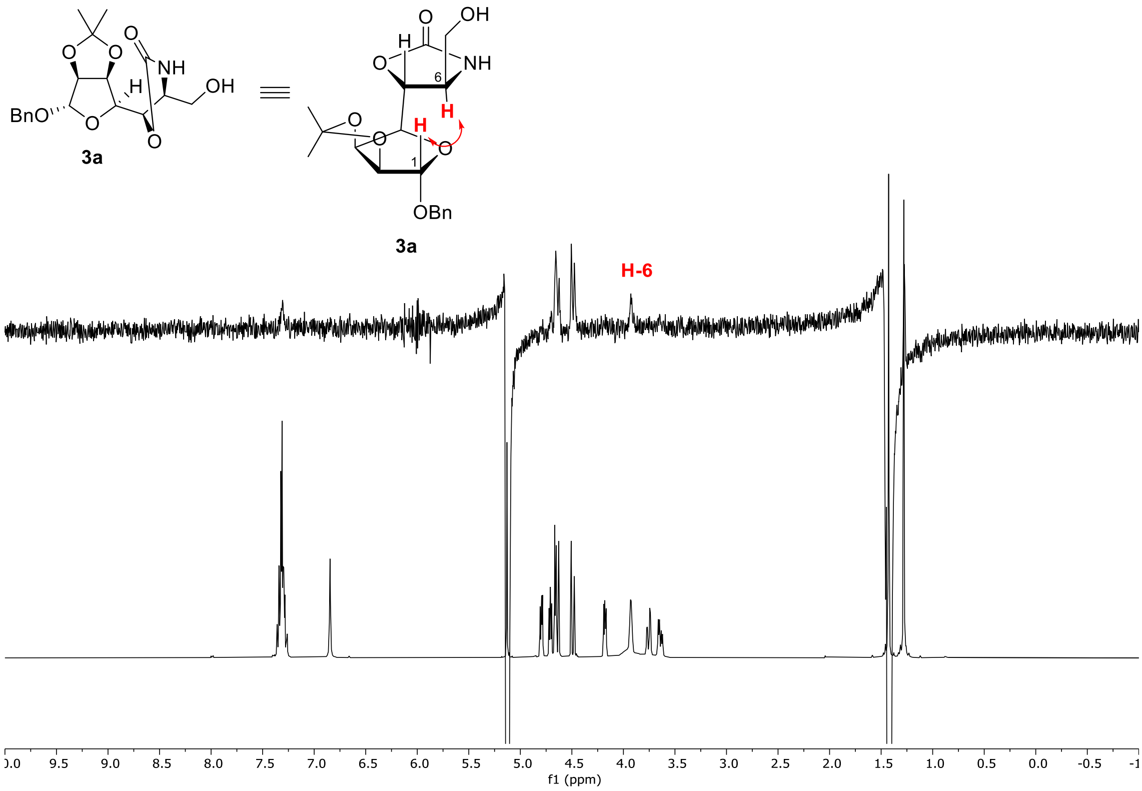

Figure S16. 1D NOESY spectrum of compound **3a** (Irradiation of H-1 at 5.13 ppm, $CDCl_3$, 400 MHz)

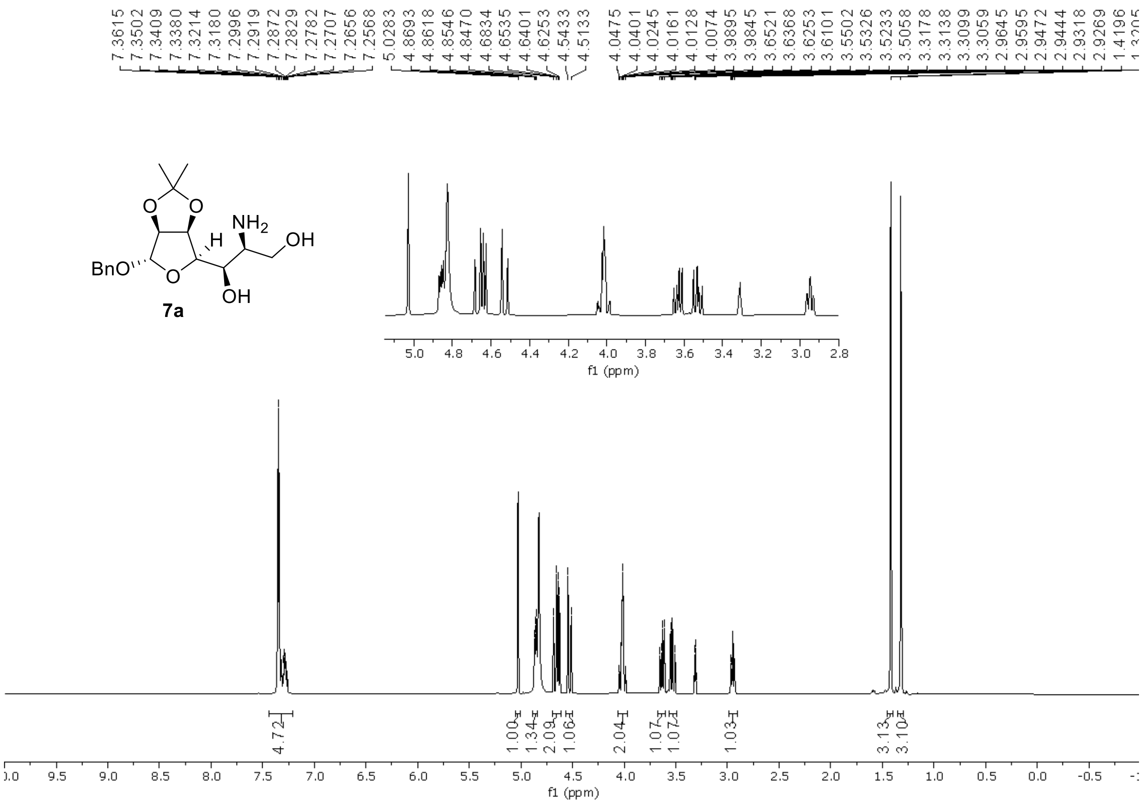

Figure S17. ^{1}H NMR spectrum of compound **7a** (CD_3OD, 400 MHz)

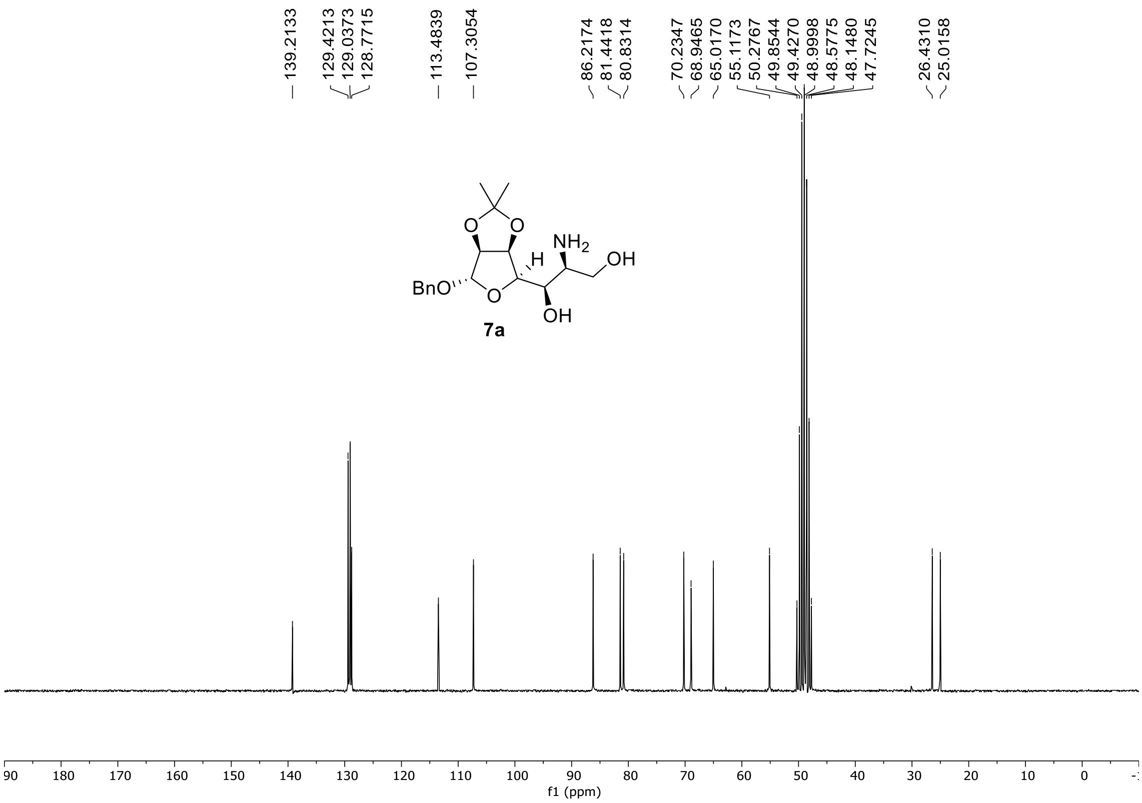

Figure S18. $^{13}C\{^{1}H\}$ NMR spectrum of compound **7a** (CD_3OD, 50 MHz)

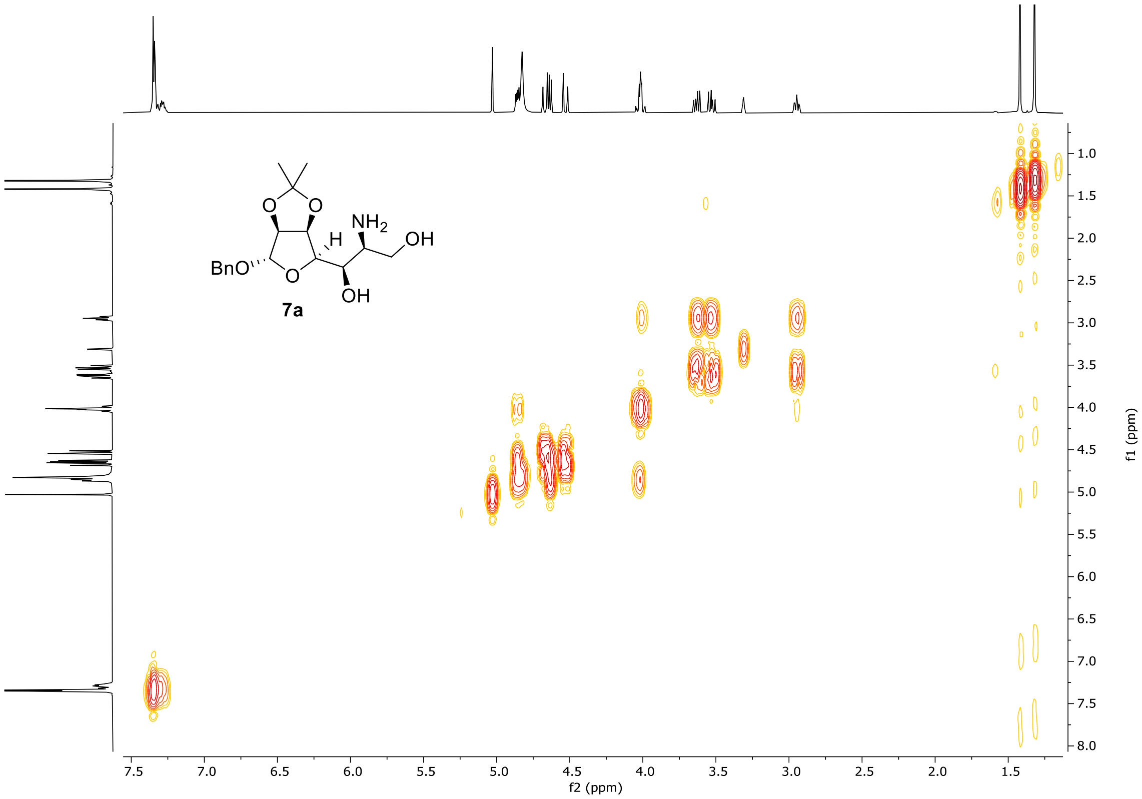

Figure S19. $^{1}H/^{1}H$ gCOSY spectrum of compound **7a** (CD_3OD, 400 MHz)

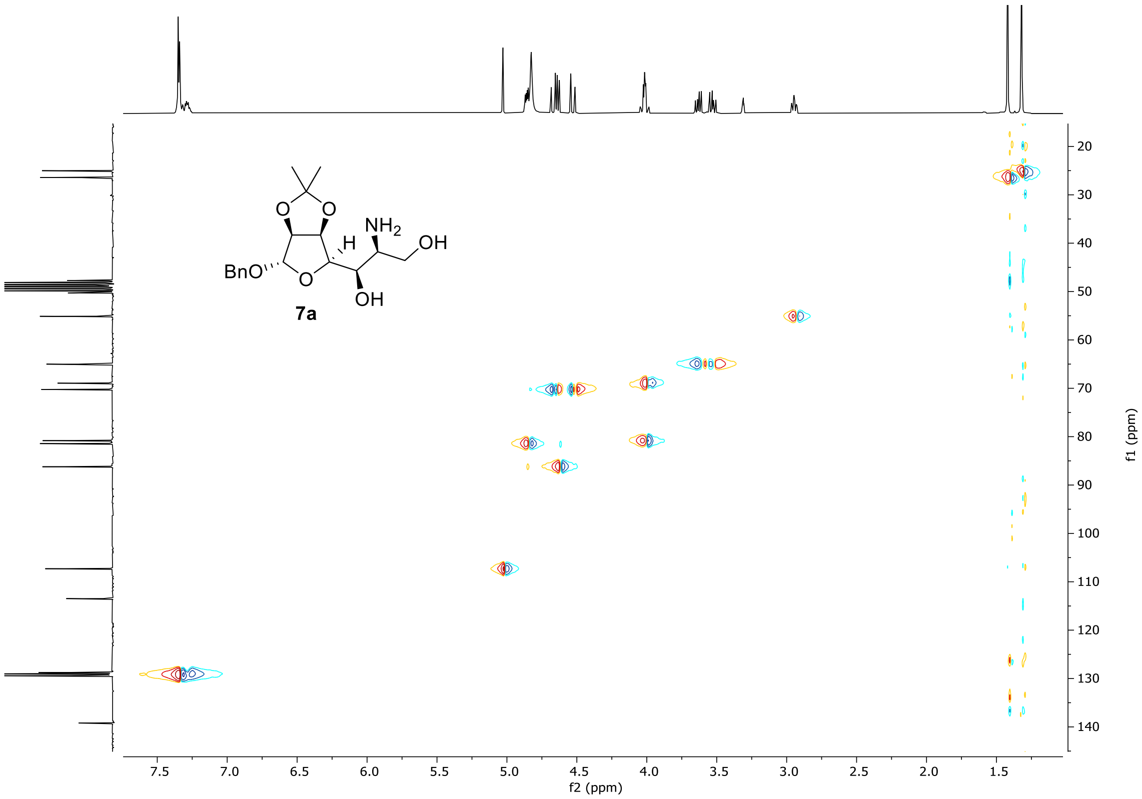

Figure S20. $^{1}H/^{13}C$ gHSQC spectrum of compound **7a** (CD_3OD, 400/100 MHz)

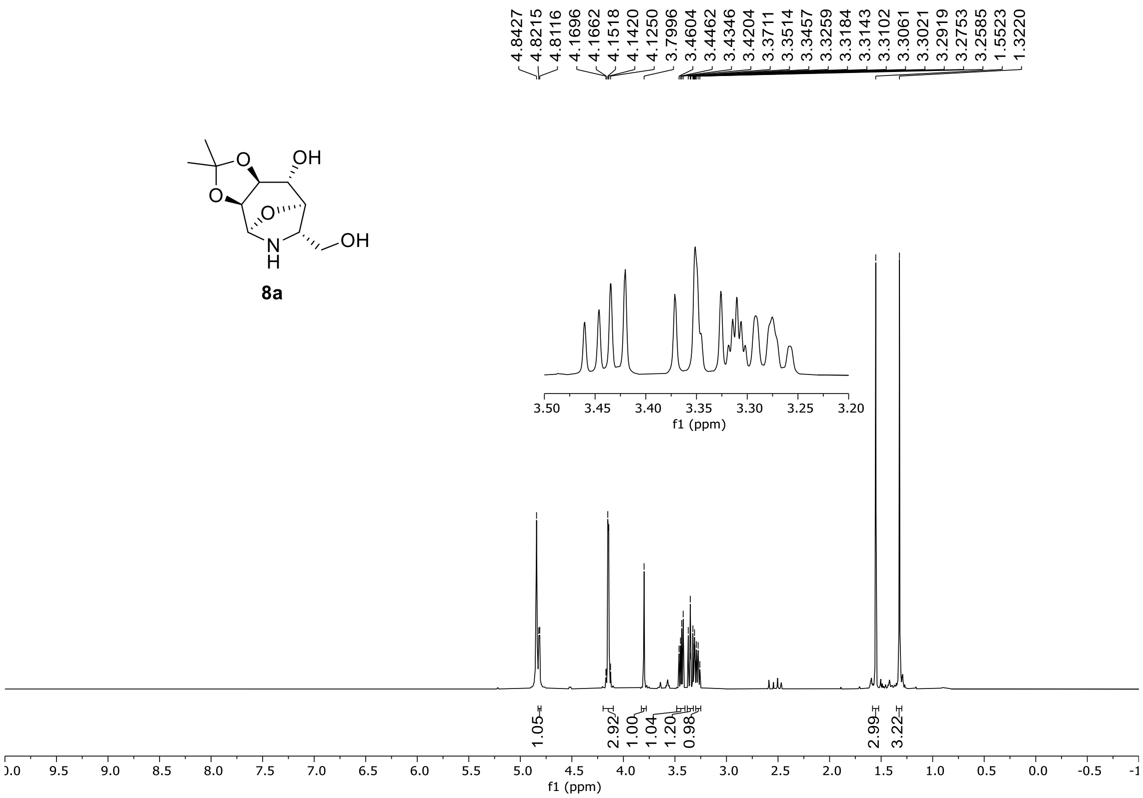

Figure S21. ^{1}H NMR spectrum of compound **8a** (CD_3OD, 400 MHz)

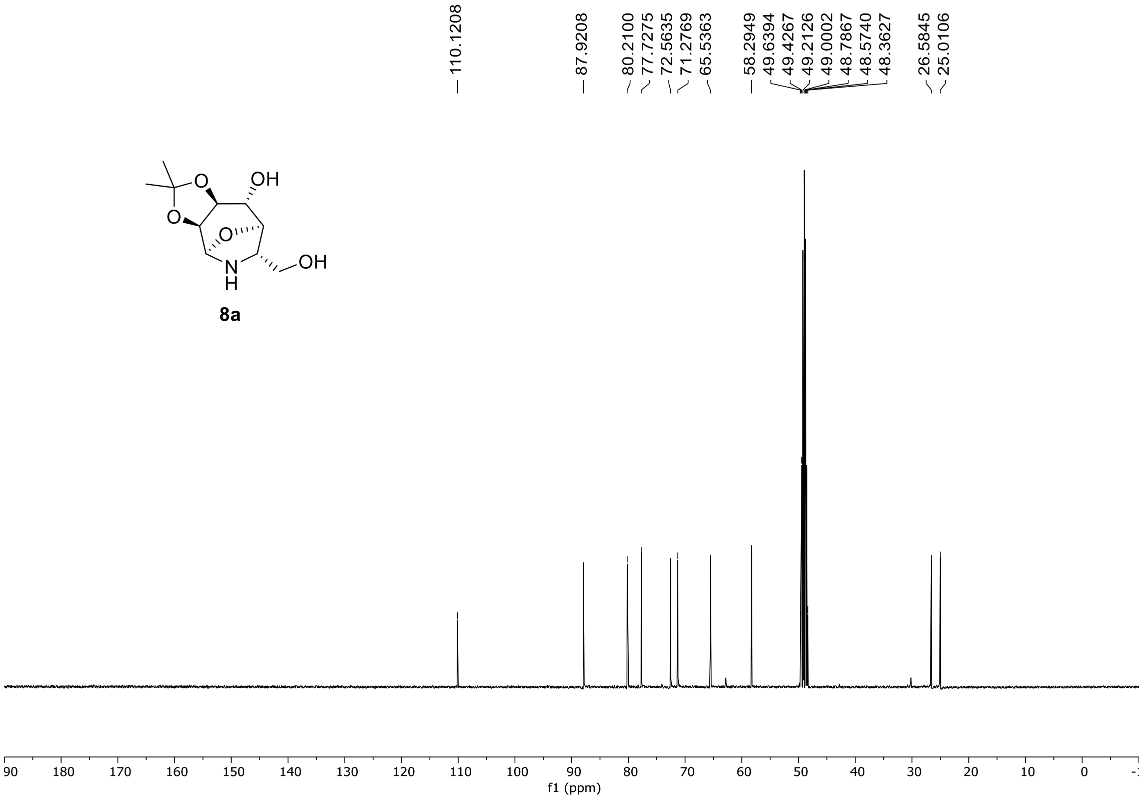

Figure S22. $^{13}C\{^{1}H\}$ NMR spectrum of compound **8a** (CD_3OD, 100 MHz)

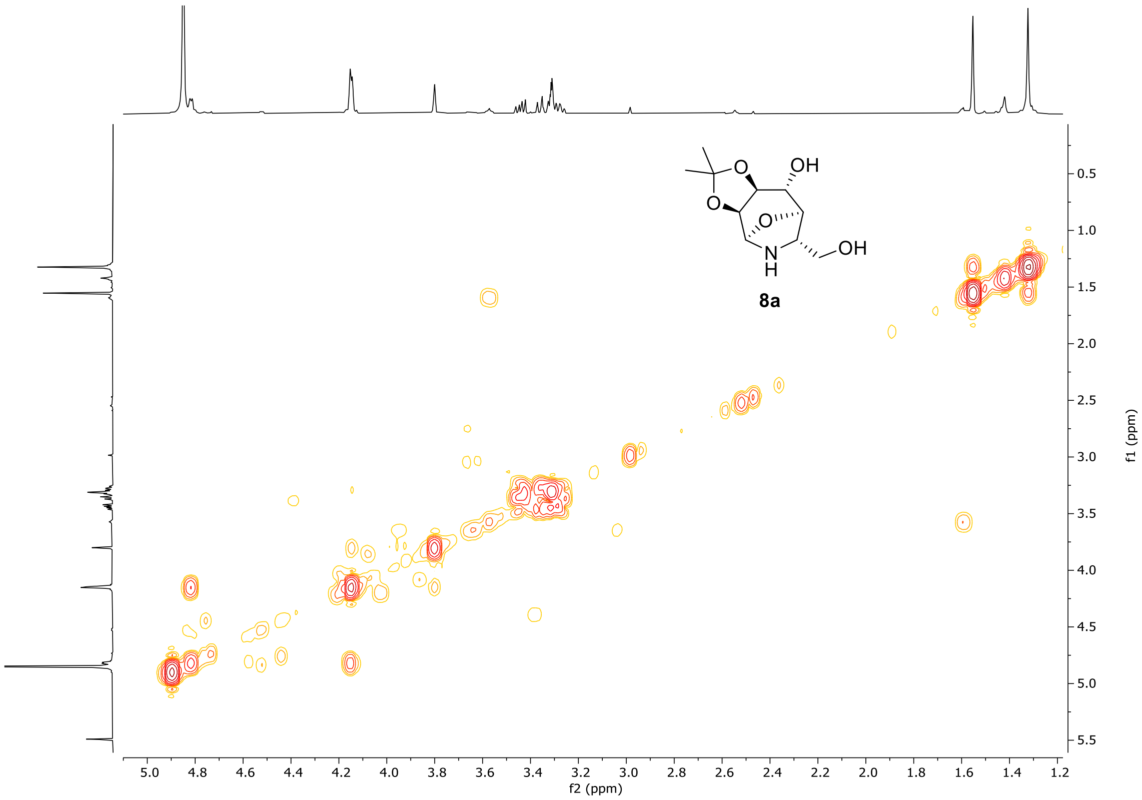

Figure S23. ¹H/¹H gCOSY spectrum of compound **8a** (CD_3OD, 400 MHz)

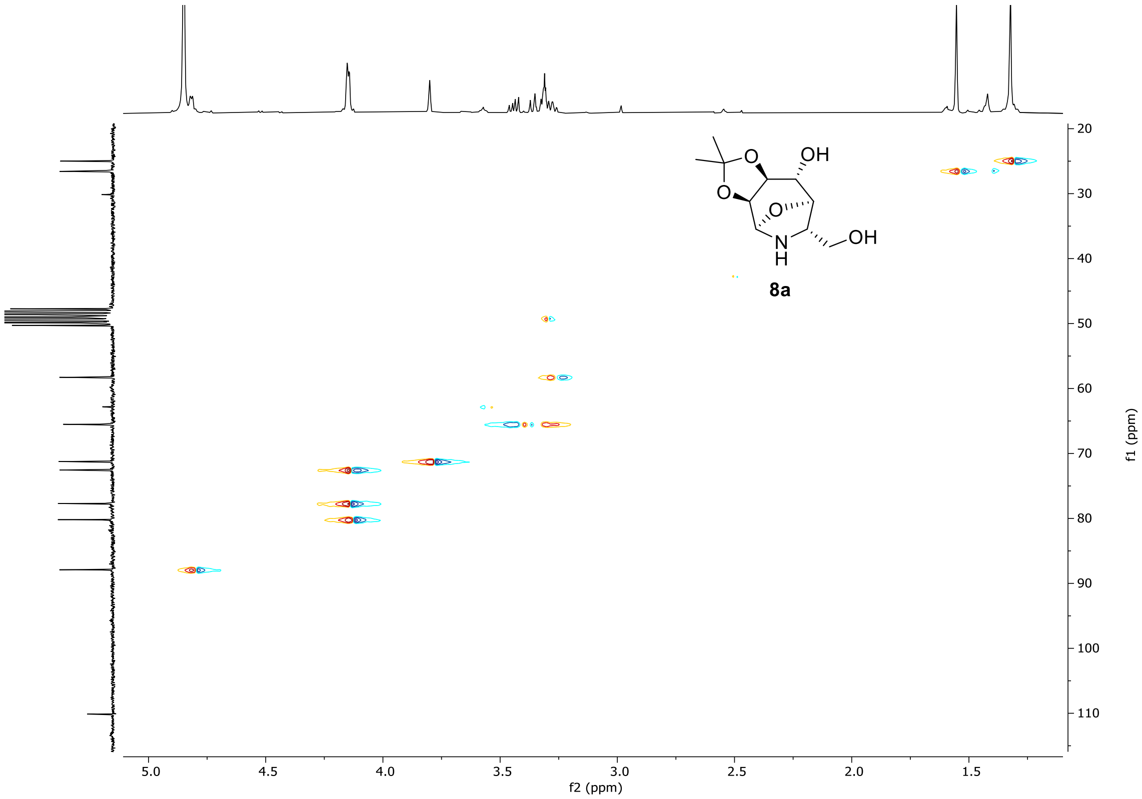

Figure S24. ¹H/¹³C gHSQC spectrum of compound **8a** (CD_3OD, 400/100 MHz)

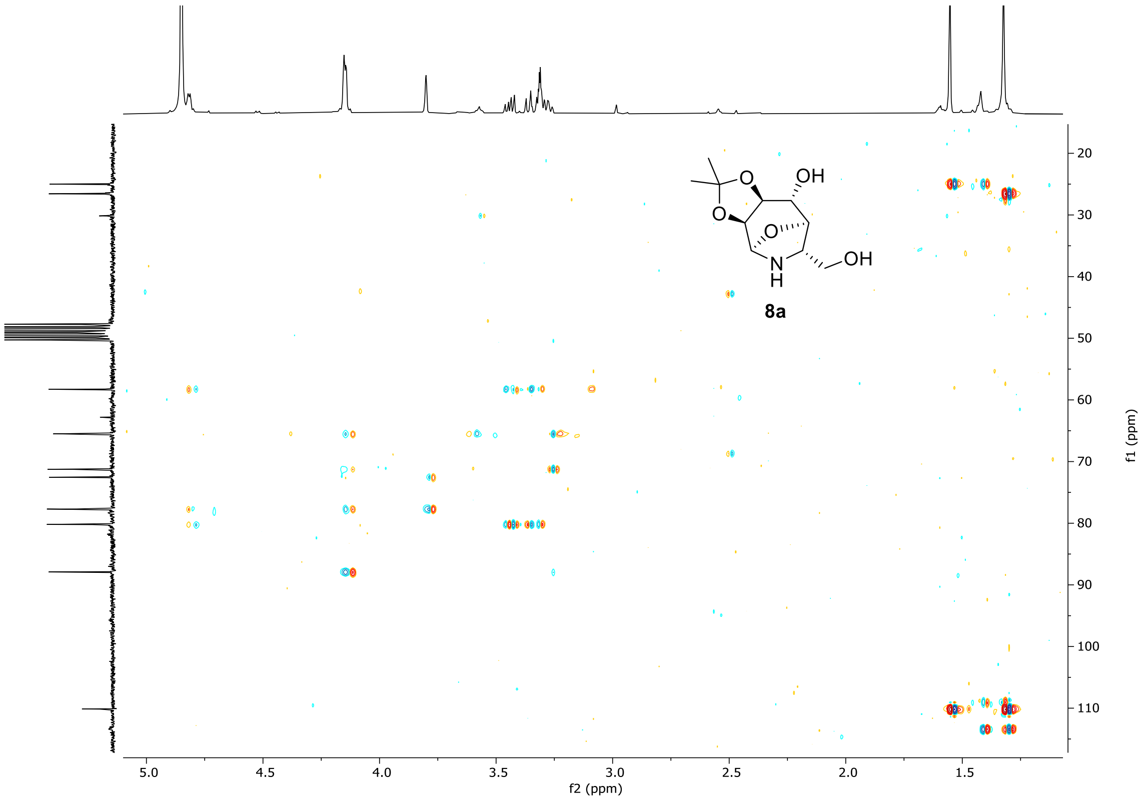

Figure S25. $^{1}H/^{13}C$ gHMBC spectrum of compound **8a** (CD_3OD, 400/100 MHz)

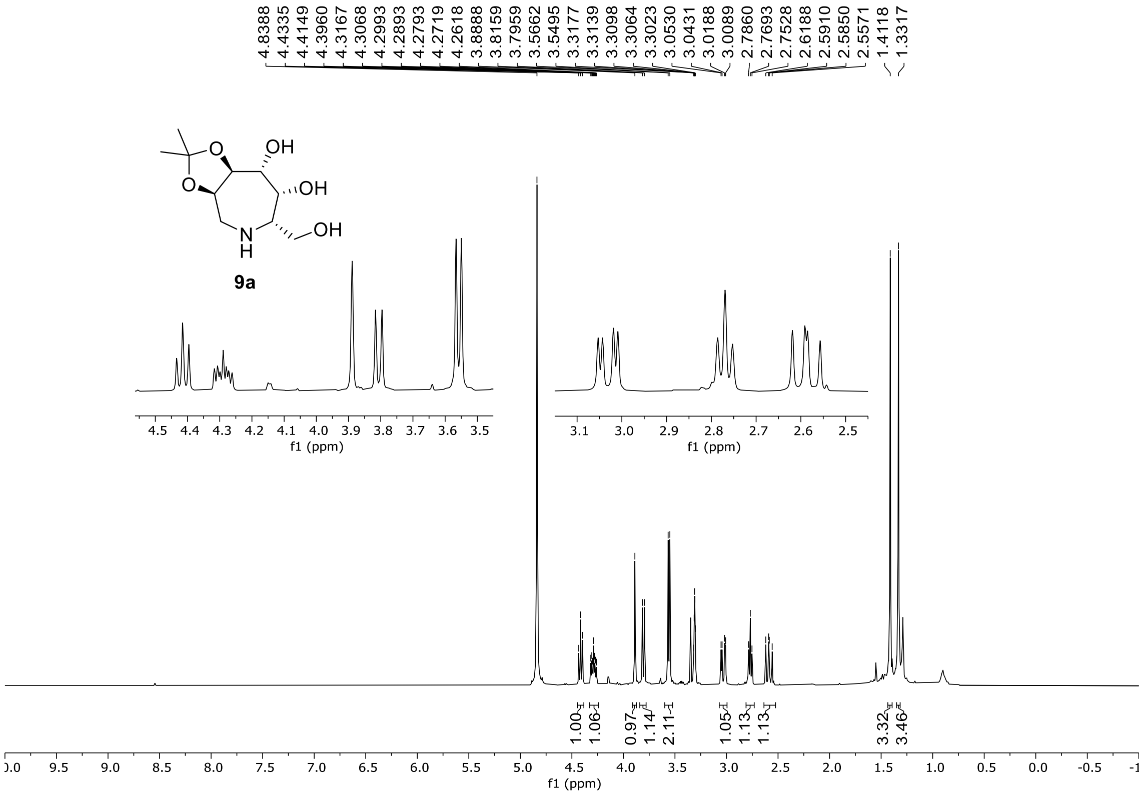

Figure S26. ^{1}H NMR spectrum of compound **9a** (CD_3OD, 400 MHz)

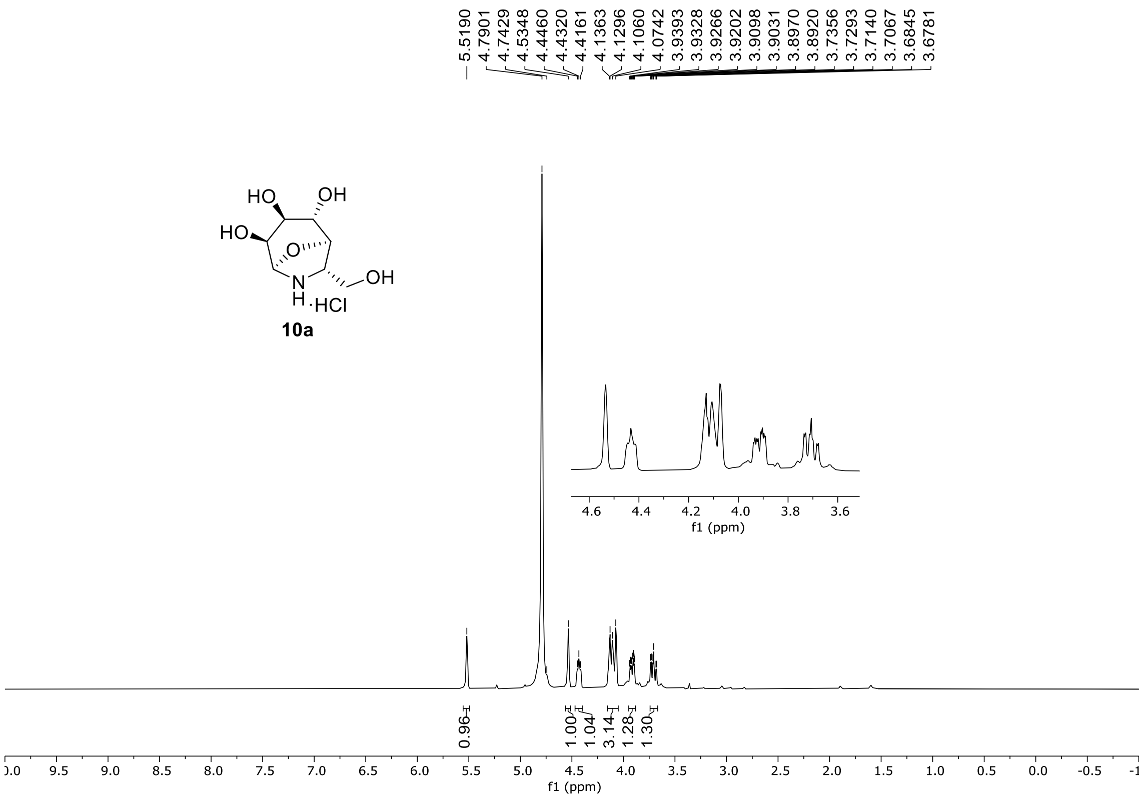

Figure S27. ^{1}H NMR spectrum of compound **10a** (D_2O, 400 MHz)

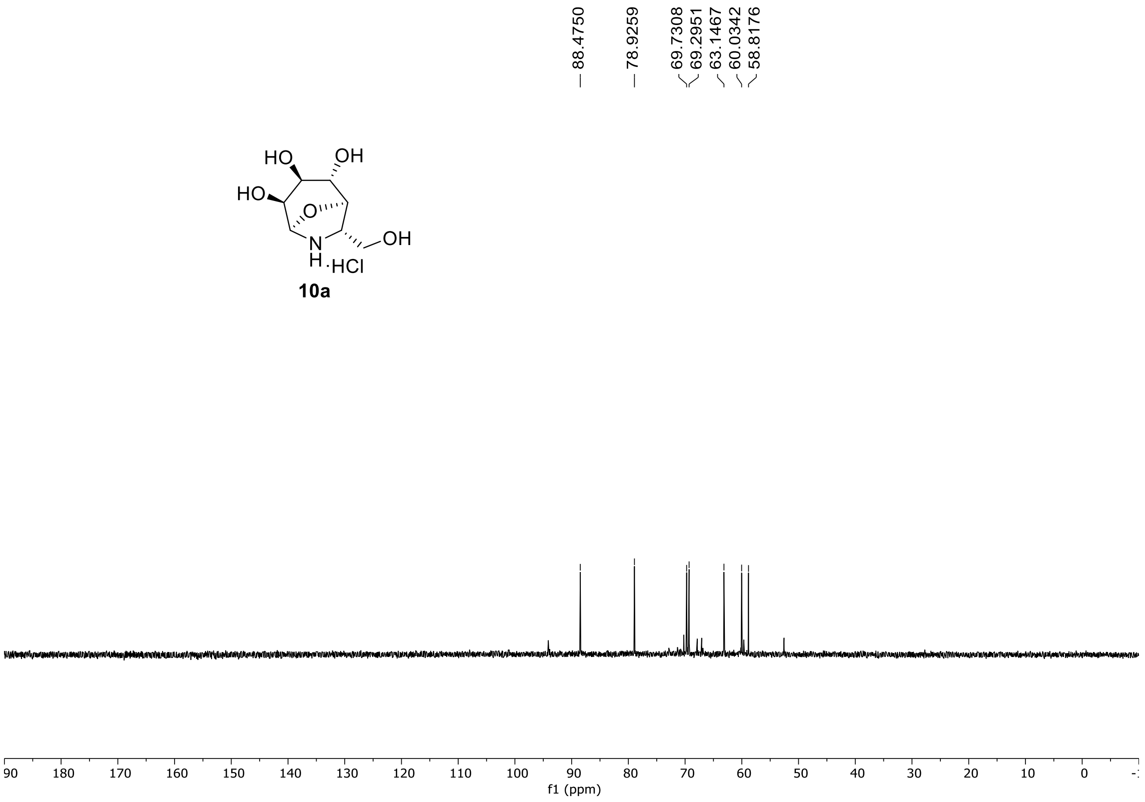

Figure S28. $^{13}C\{^{1}H\}$ NMR spectrum of compound **10a** (D_2O, 100 MHz)

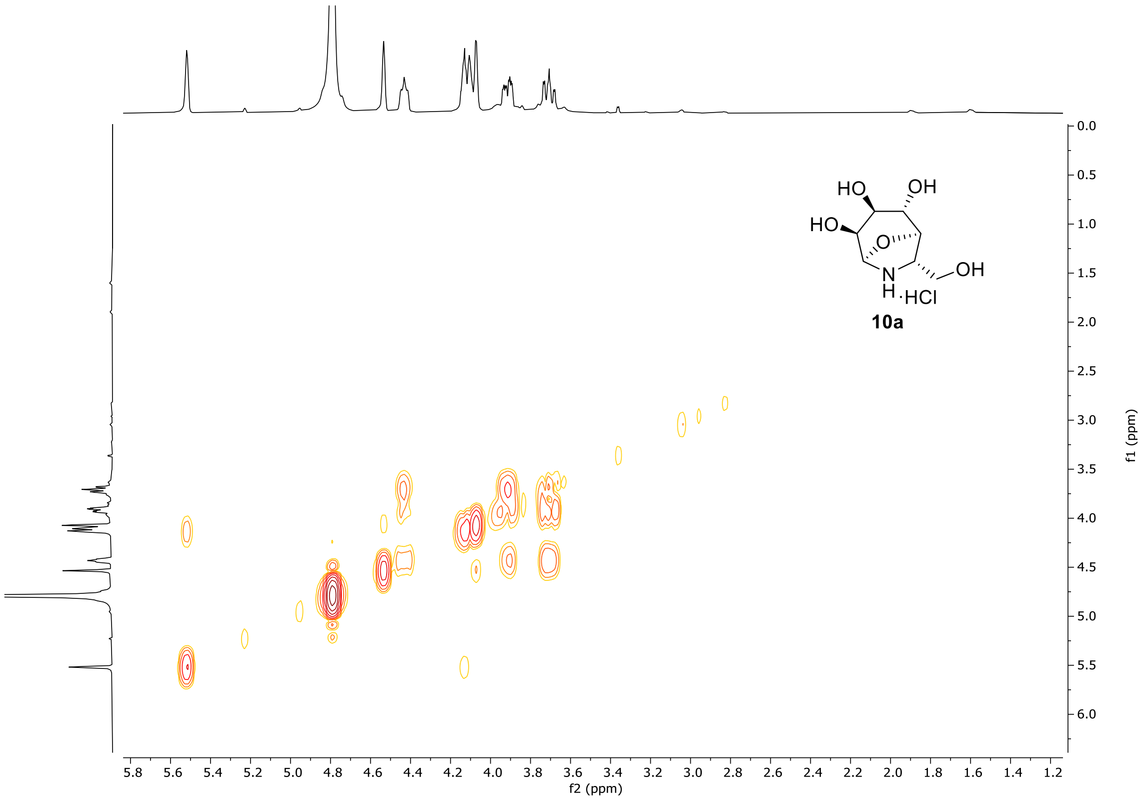

Figure S29. $^{1}H/^{1}H$ gCOSY spectrum of compound **10a** (D_2O, 400 MHz)

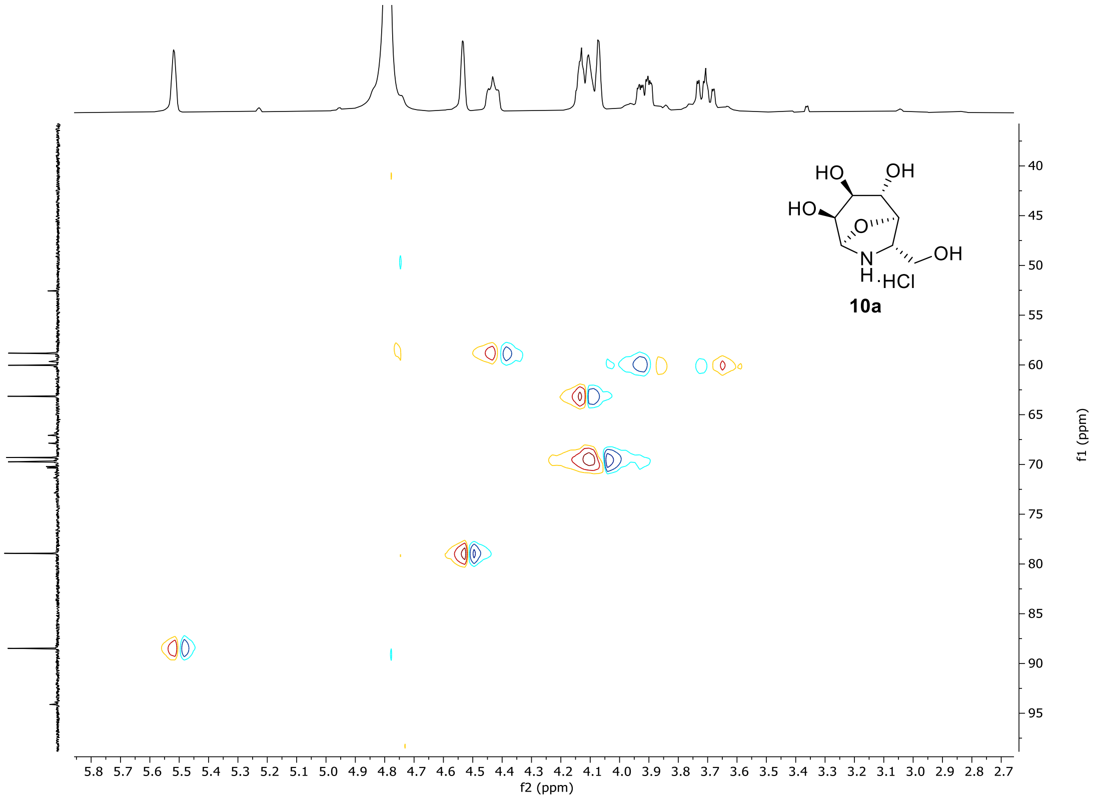

Figure S30. $^{1}H/^{13}C$ gHSQC spectrum of compound **10a** (D_2O, 400/100 MHz)

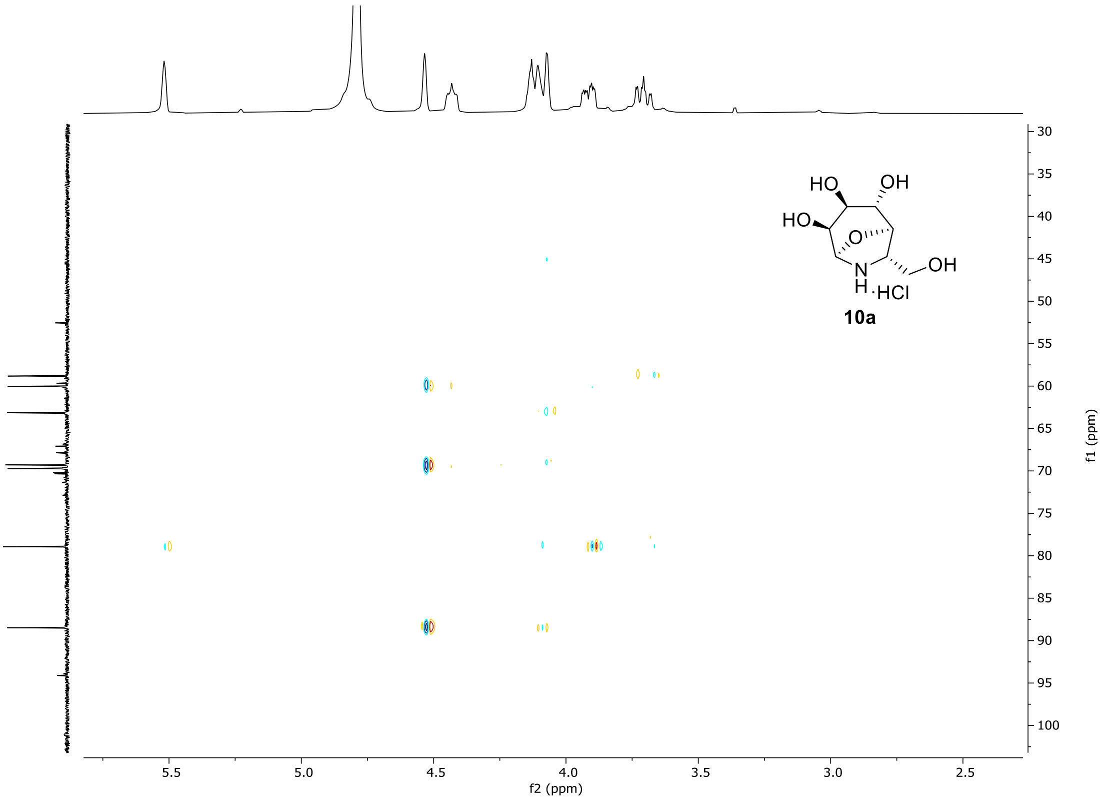

Figure S31. $^1H/^{13}C$ gHMBC spectrum of compound **10a** (D_2O, 400/100 MHz)

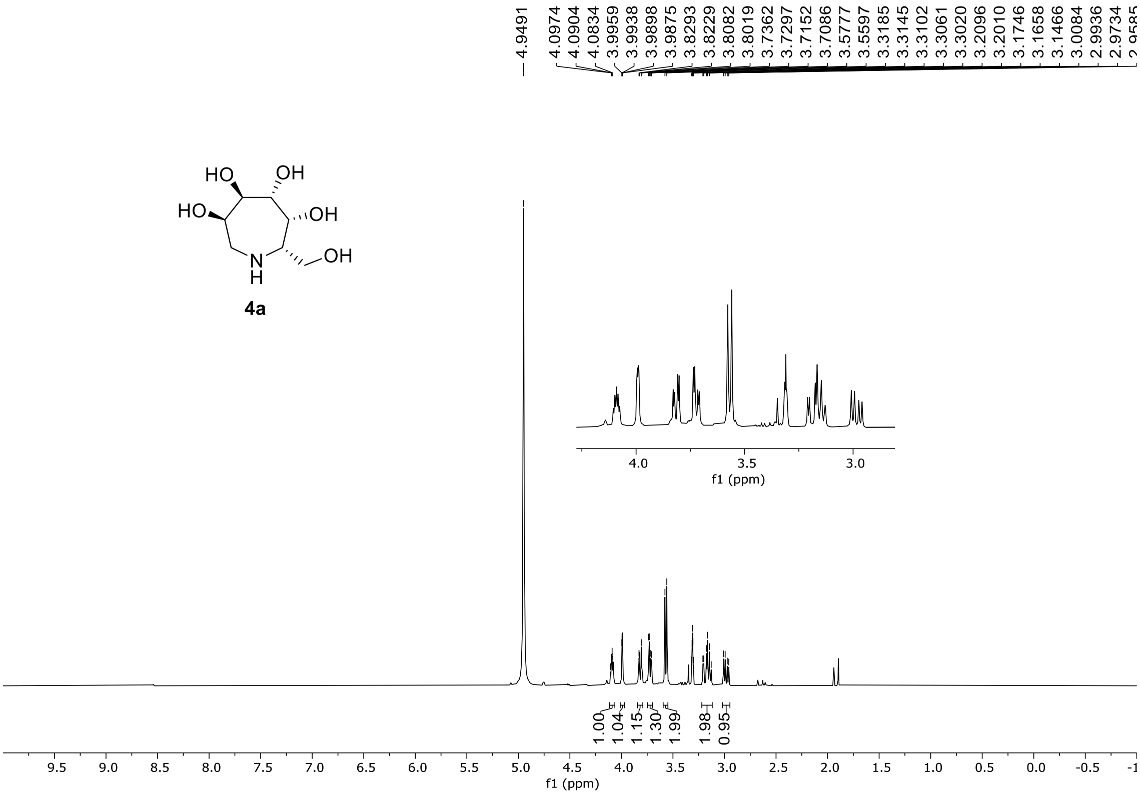

Figure S32. ^1H NMR spectrum of compound **4a** (CD_3OD, 400 MHz)

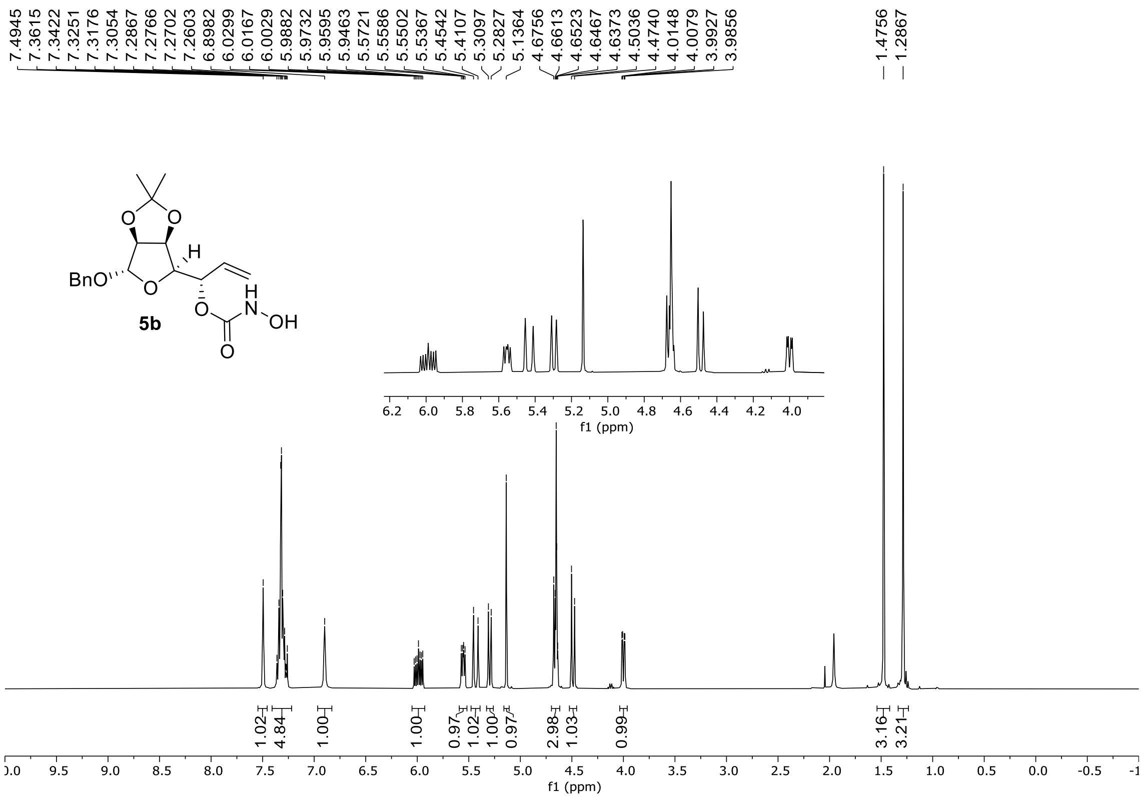

Figure S33. ^{1}H NMR spectrum of compound **5b** ($CDCl_3$, 400 MHz)

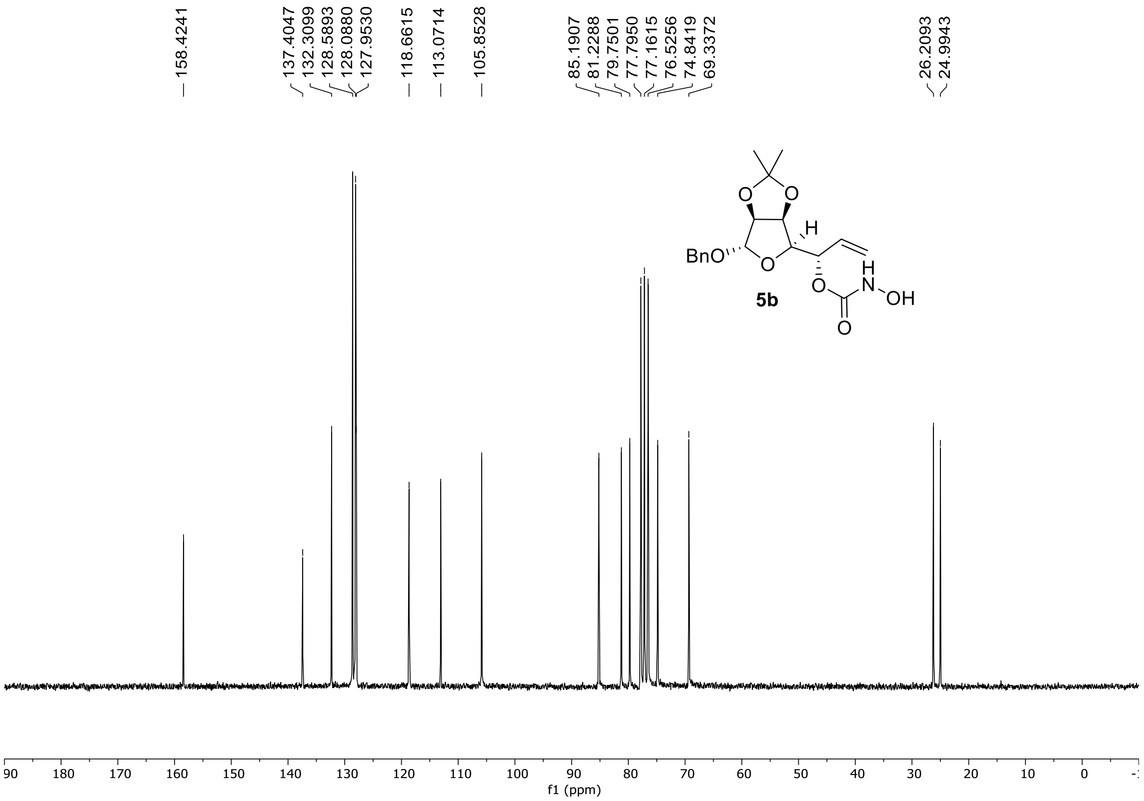

Figure S34. $^{13}C\{^{1}H\}$ NMR spectrum of compound **5b** ($CDCl_3$, 50 MHz)

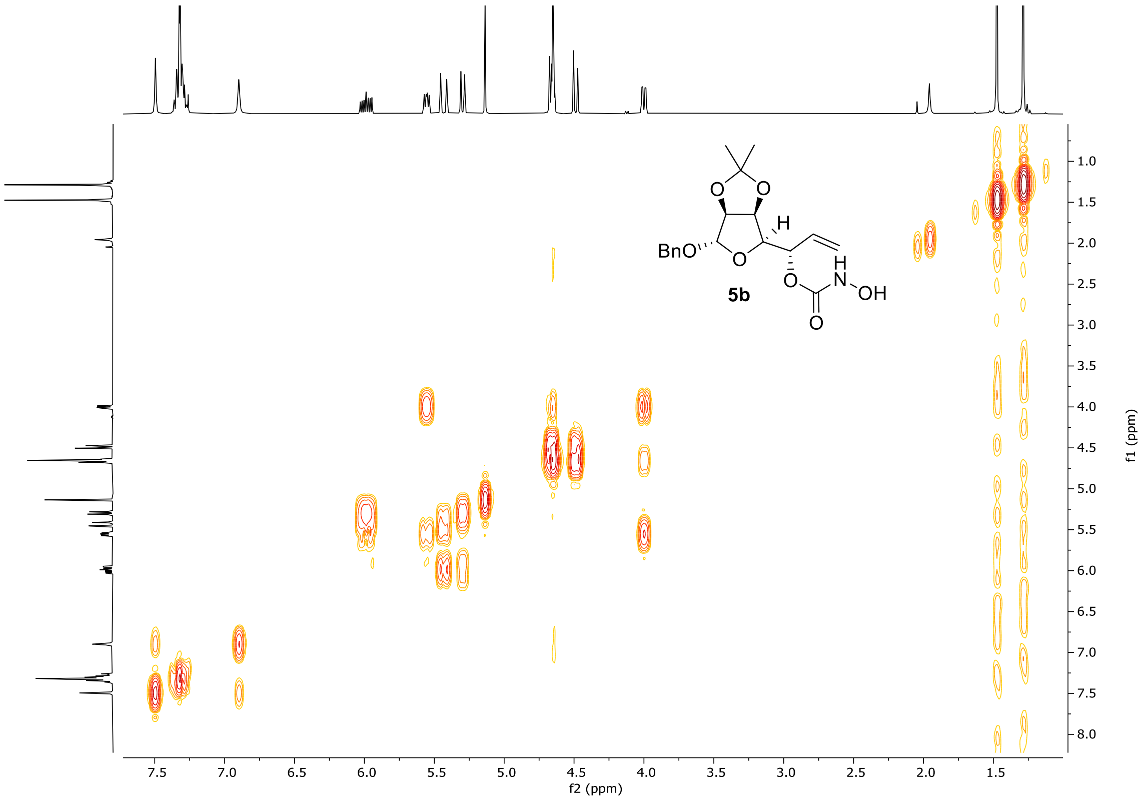

Figure S35. $^1H/^1H$ gCOSY spectrum of compound **5b** ($CDCl_3$, 400 MHz)

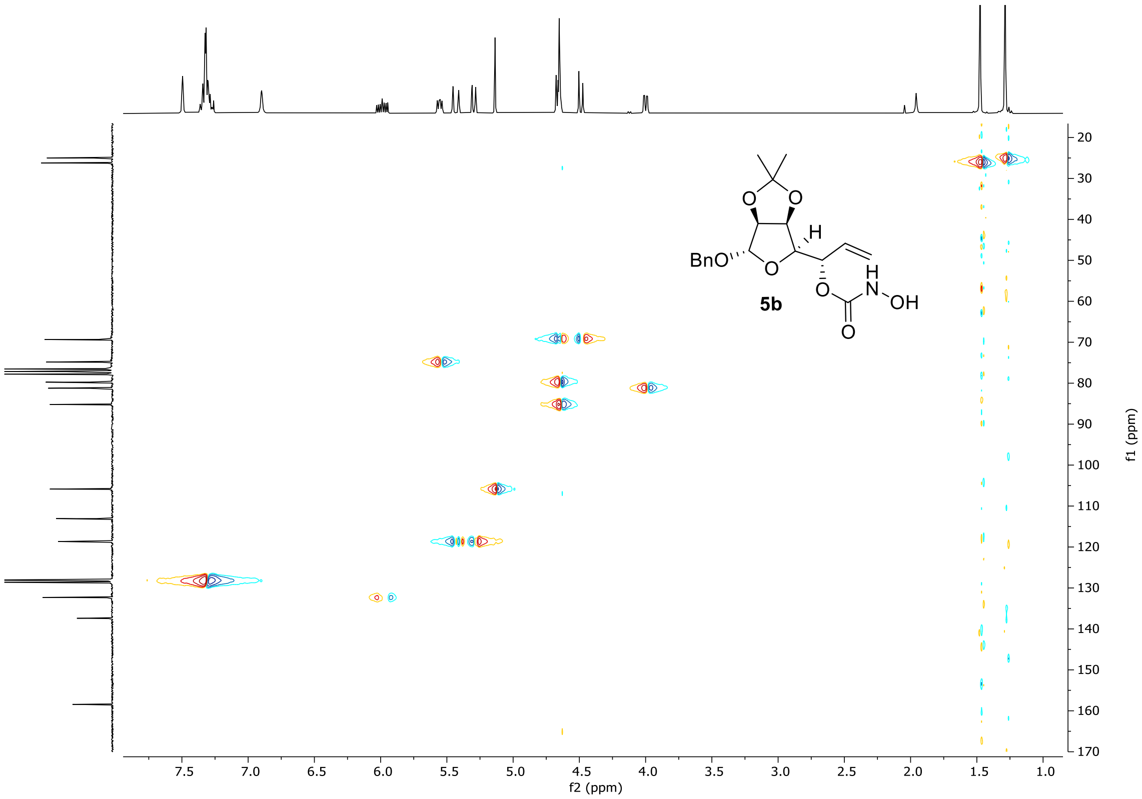

Figure S36. $^1H/^{13}C$ gHSQC spectrum of compound **5b** ($CDCl_3$, 400/100 MHz)

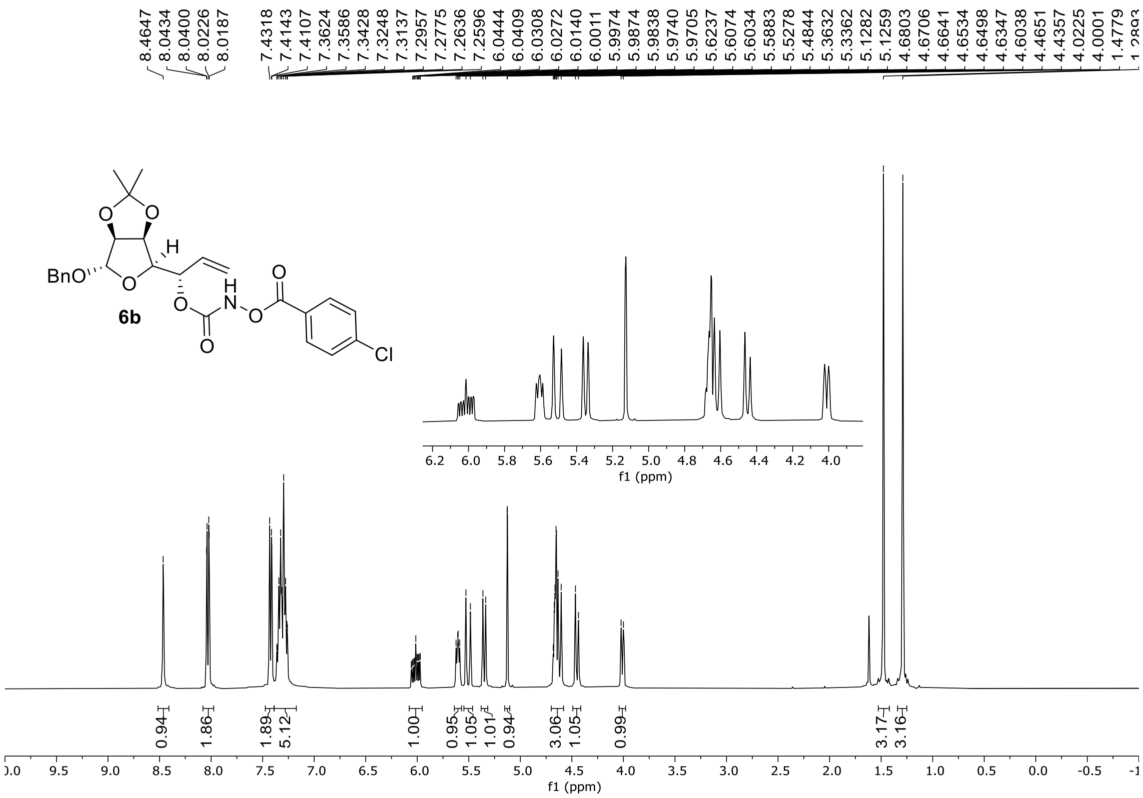

Figure S37. ^{1}H NMR spectrum of compound **6b** ($CDCl_3$, 400 MHz)

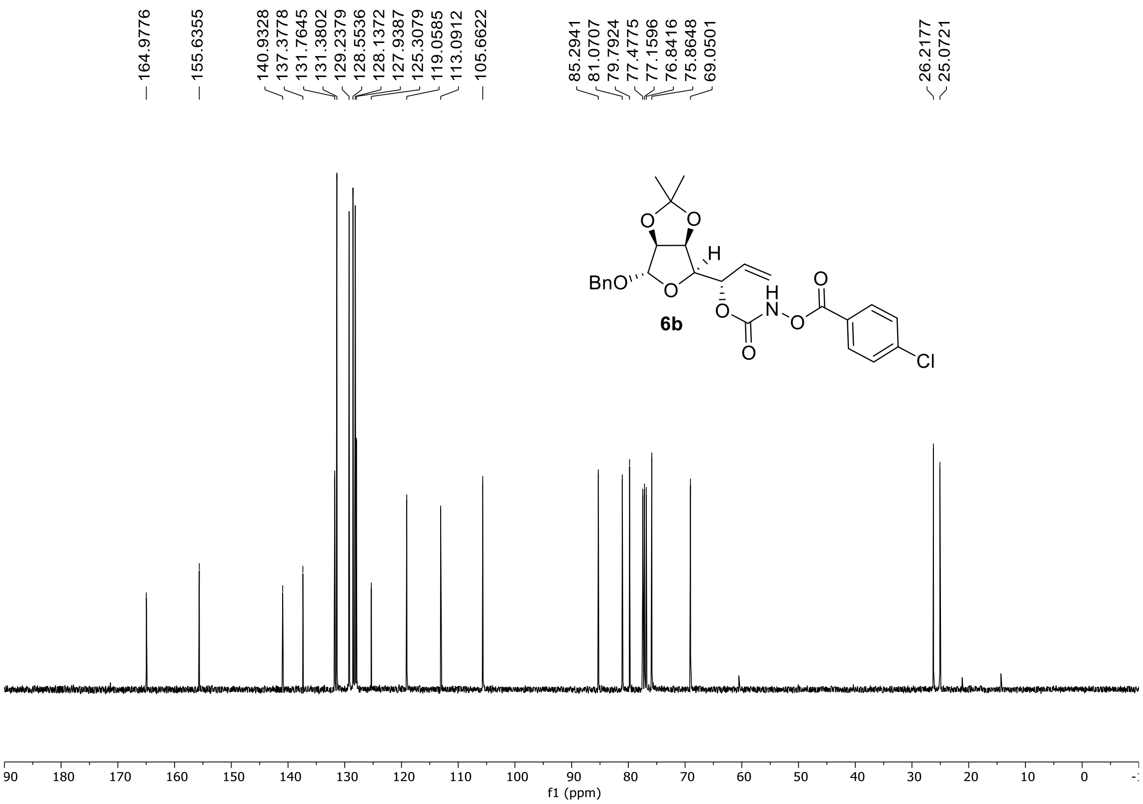

Figure S38. $^{13}C\{^{1}H\}$ NMR spectrum of compound **6b** ($CDCl_3$, 100 MHz)

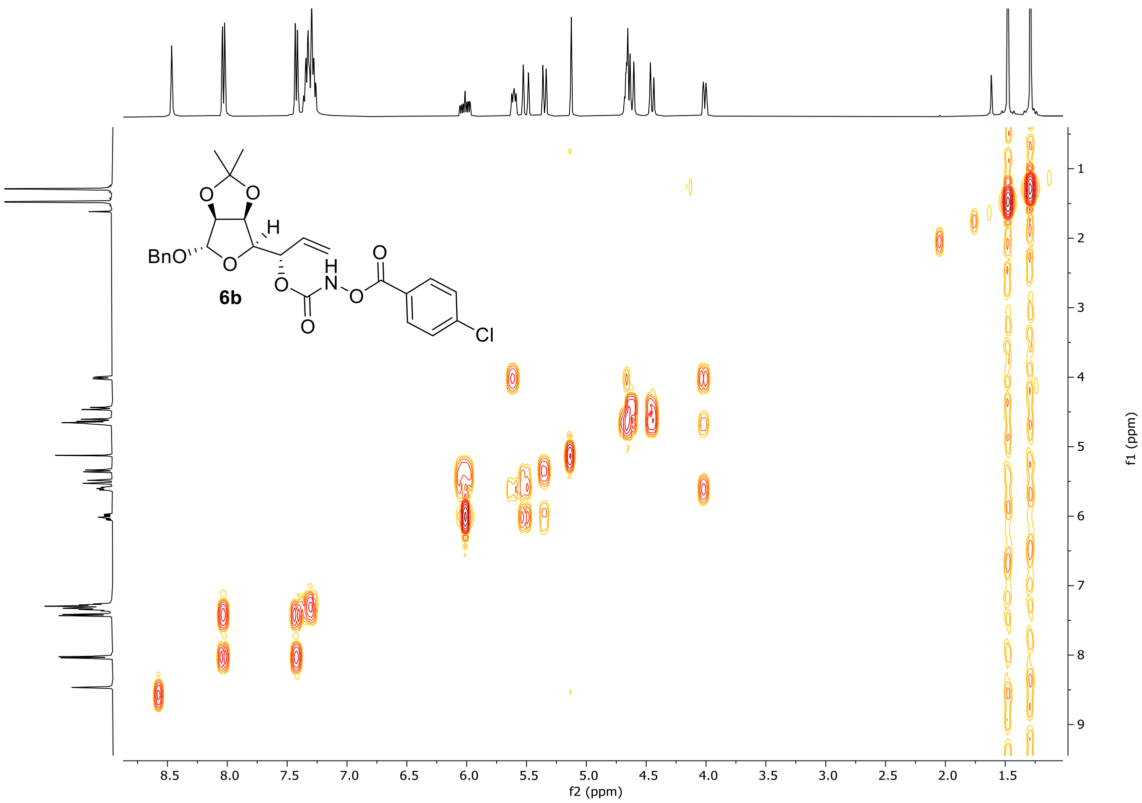

Figure S39. ¹H/¹H gCOSY spectrum of compound **6b** ($CDCl_3$, 400 MHz)

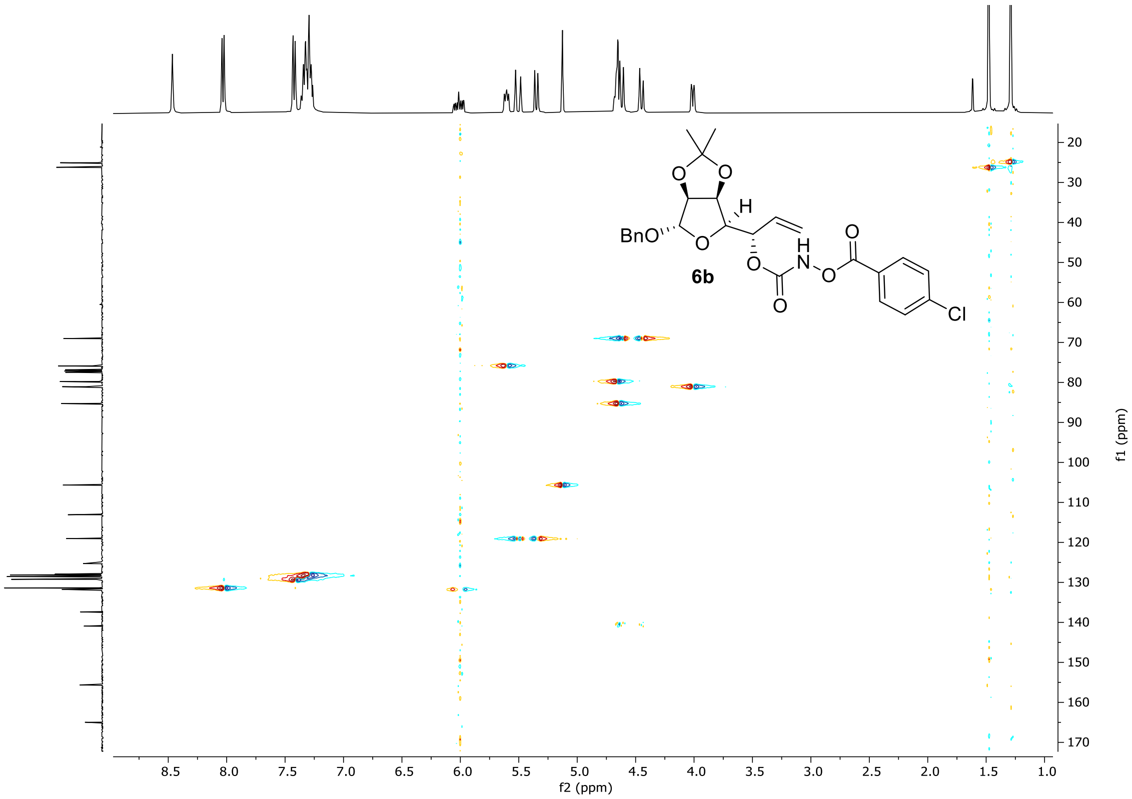

Figure S40. ¹H/¹³C gHSQC spectrum of compound **6b** ($CDCl_3$, 400/100 MHz)

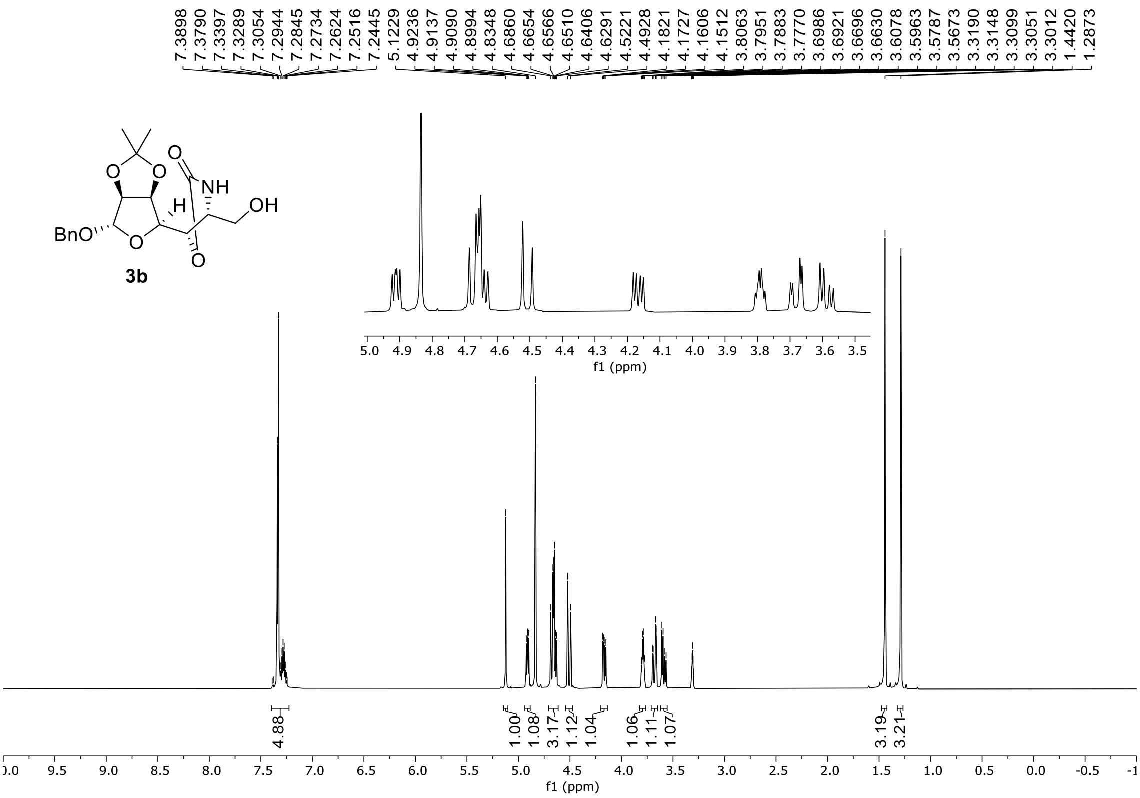

Figure S41. ^{1}H NMR spectrum of compound **3b** (CD_3OD, 400 MHz)

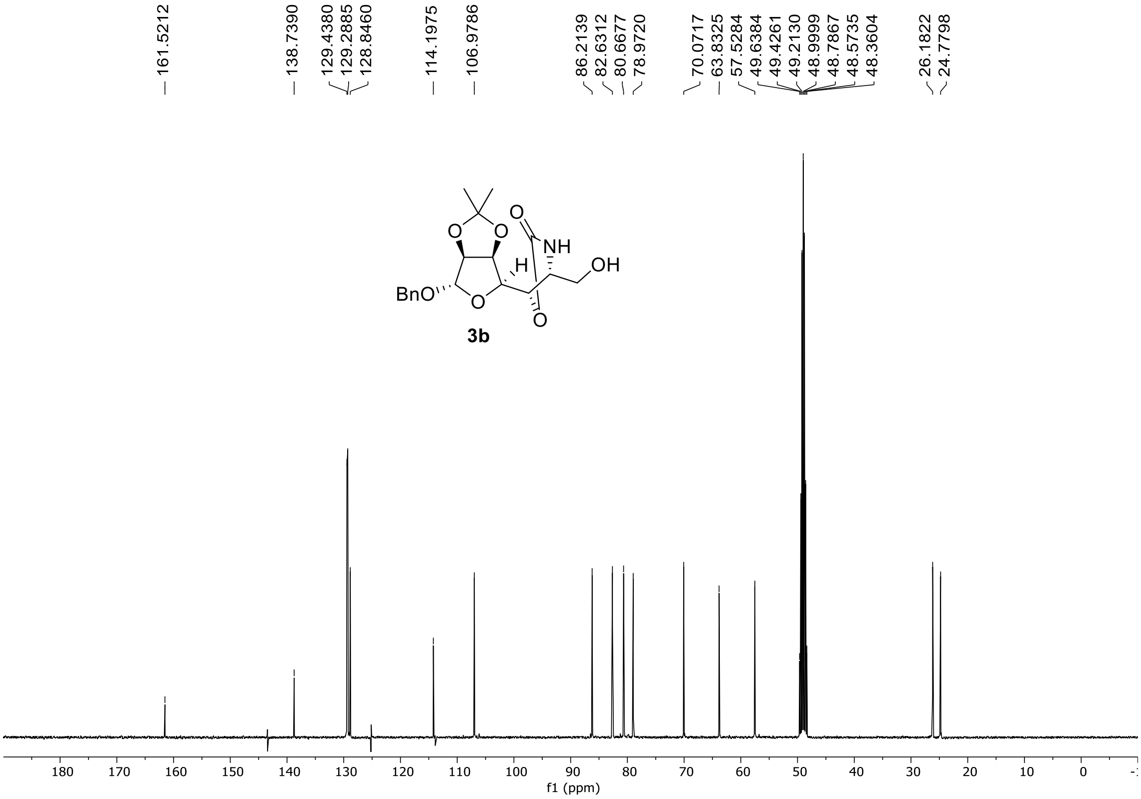

Figure S42. $^{13}C\{^{1}H\}$ NMR spectrum of compound **3b** (CD_3OD, 100 MHz)

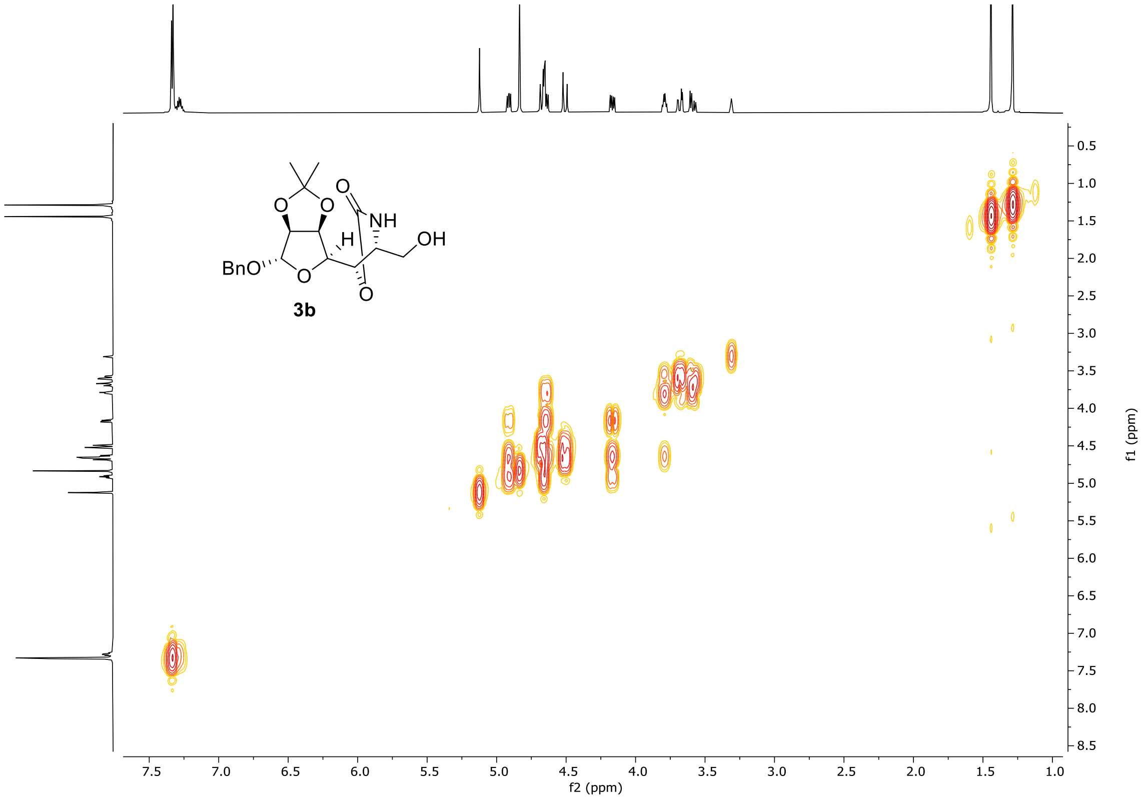

Figure S43. ^{1}H/^{1}H gCOSY spectrum of compound **3b** (CD_3OD, 400 MHz)

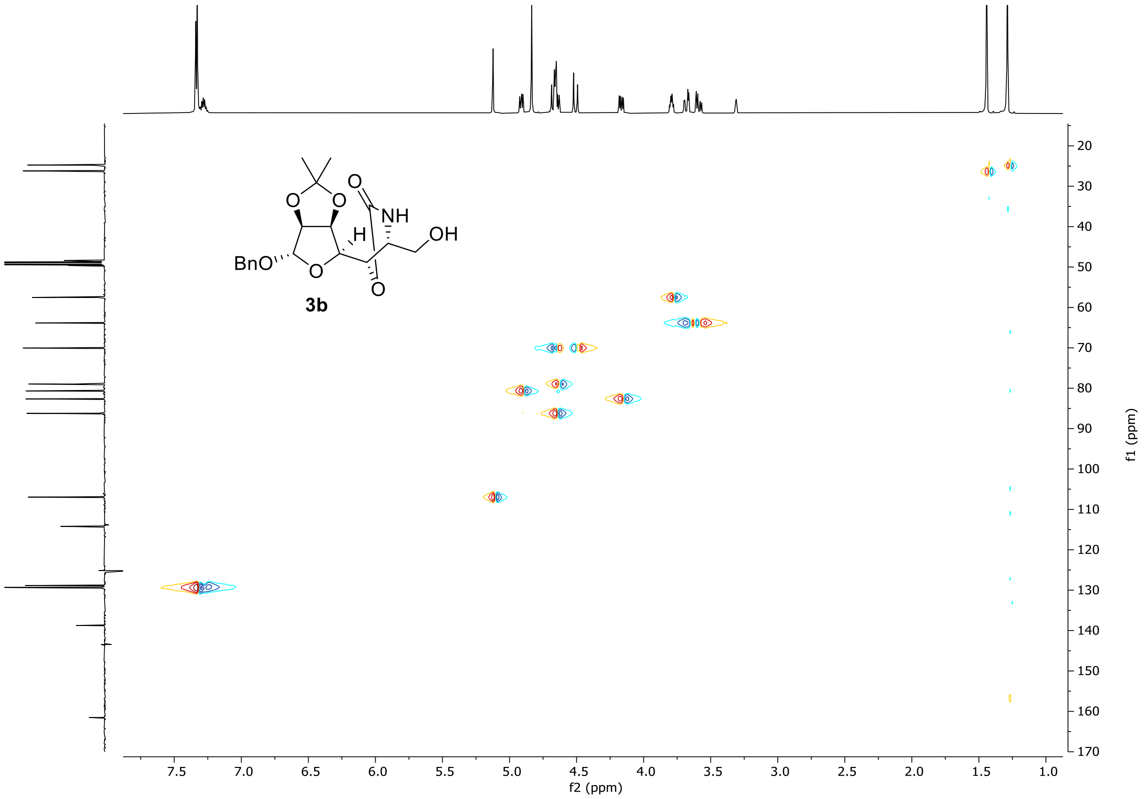

Figure S44. ^{1}H/^{13}C gHSQC spectrum of compound **3b** (CD_3OD, 400/100 MHz)

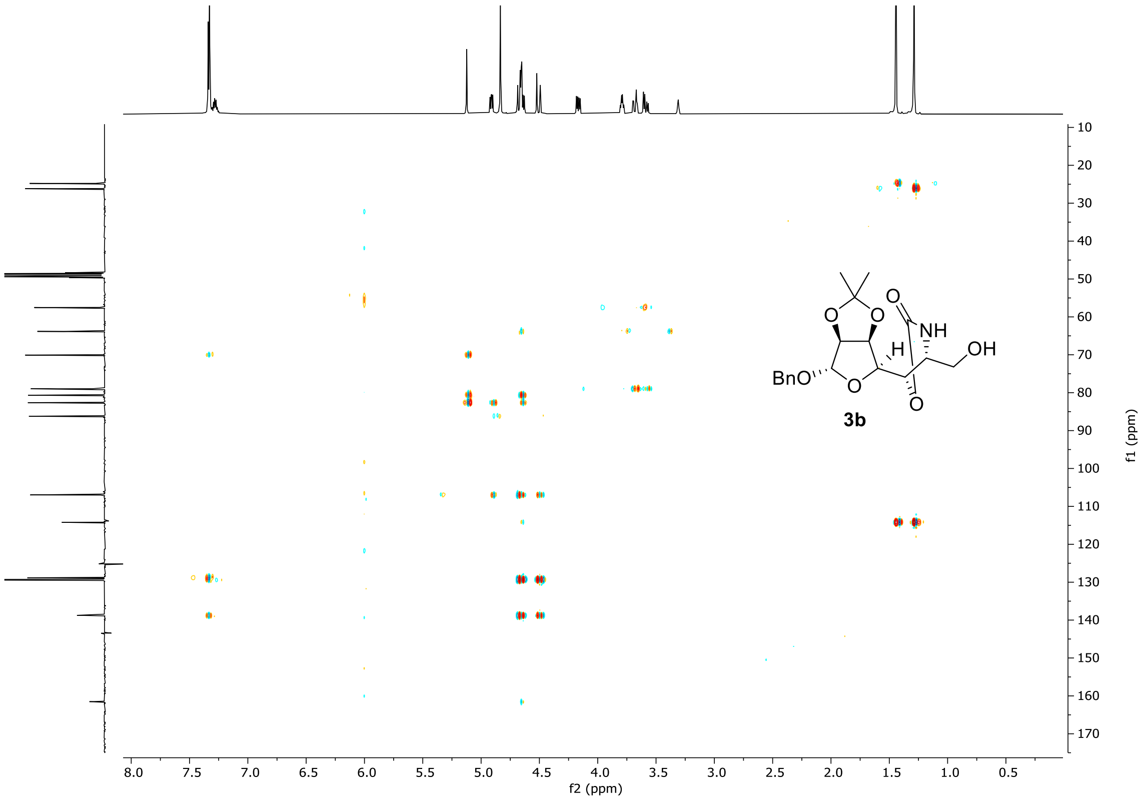

Figure S45. $^{1}H/^{13}C$ gHMBC spectrum of compound **3b** (CD_3OD, 400/100 MHz)

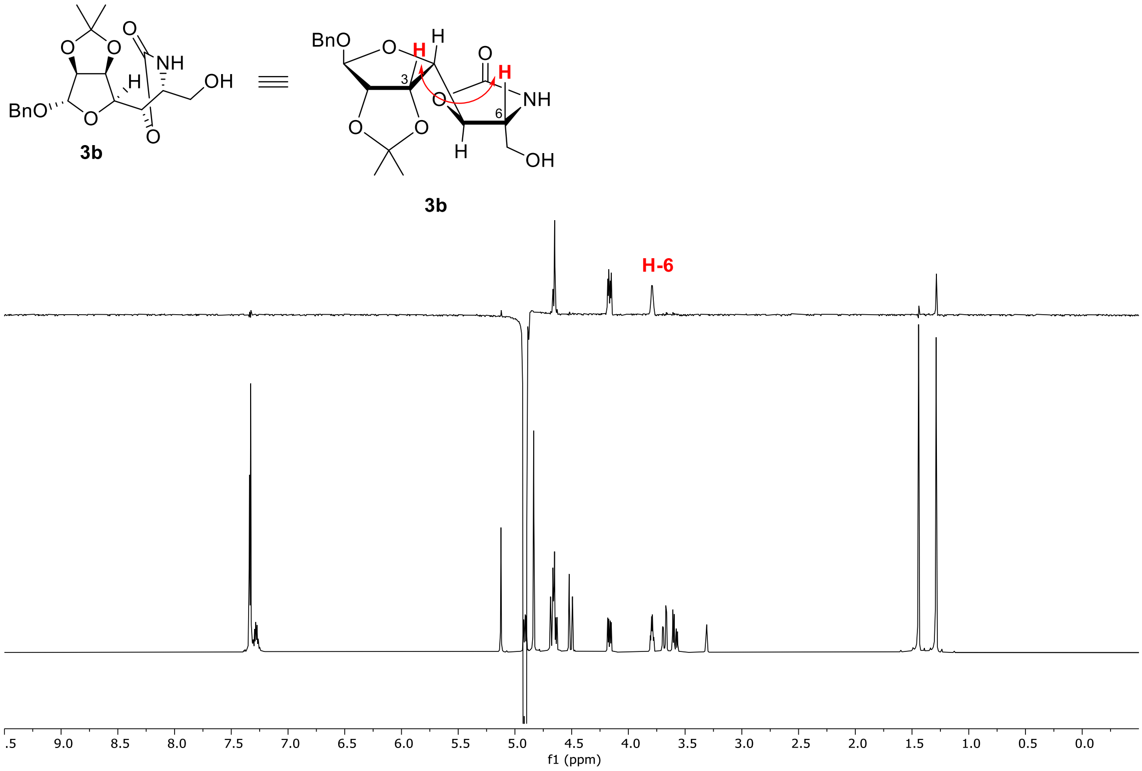

Figure S46. 1D NOESY spectrum of compound **3b** (Irradiation of H-3 at 4.91 ppm, CD_3OD, 400 MHz)

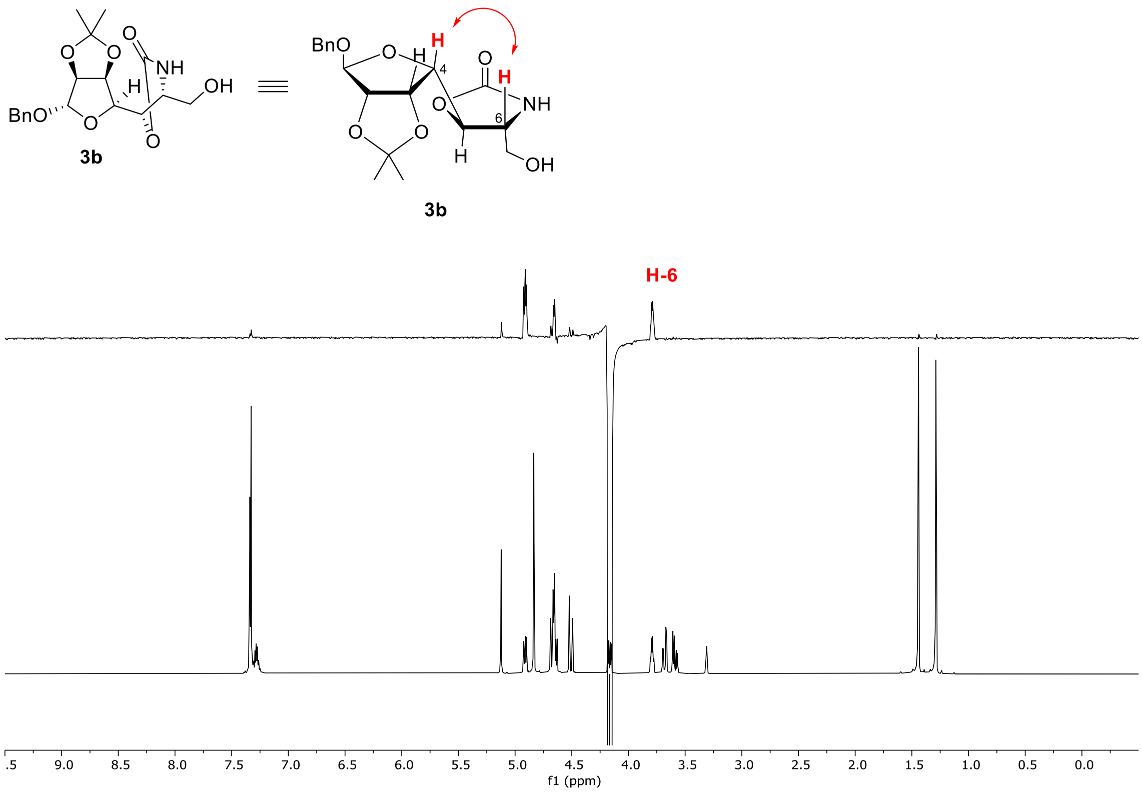

Figure S47. 1D NOESY spectrum of compound **3b** (Irradiation of H-4 at 4.17 ppm, CD_3OD, 400 MHz)

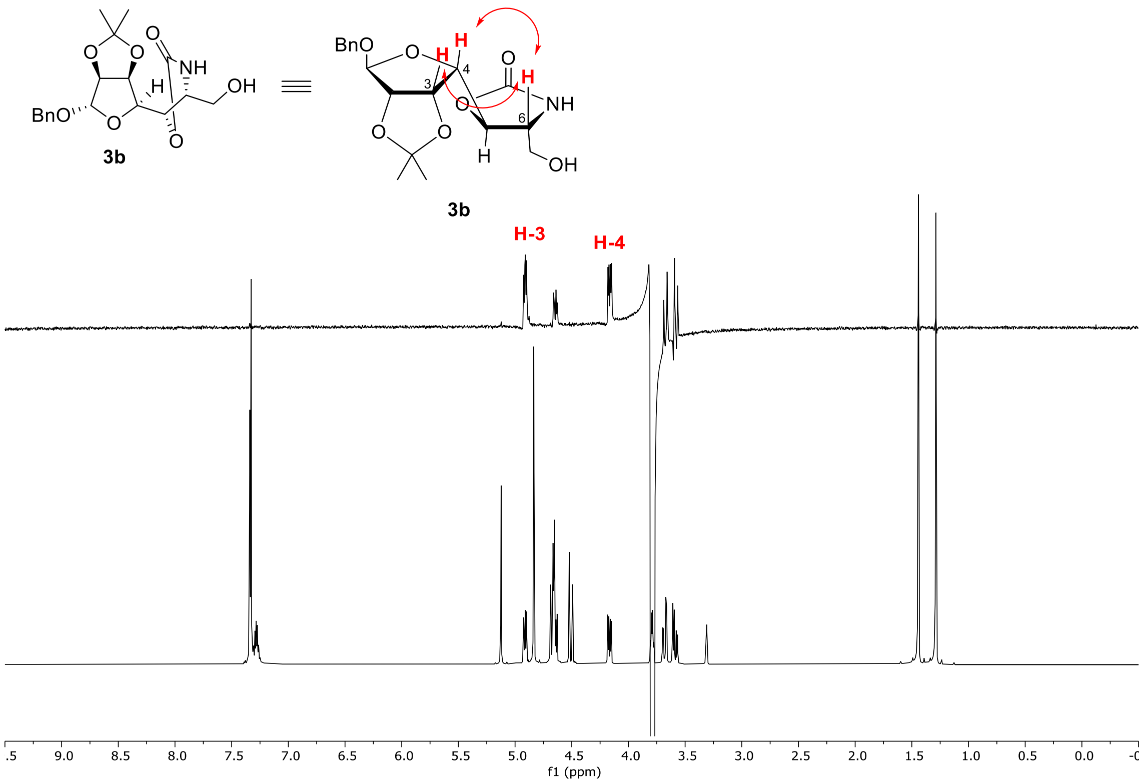

Figure S48. 1D NOESY spectrum of compound **3b** (Irradiation of H-6 at 3.79 ppm, CD_3OD, 400 MHz)

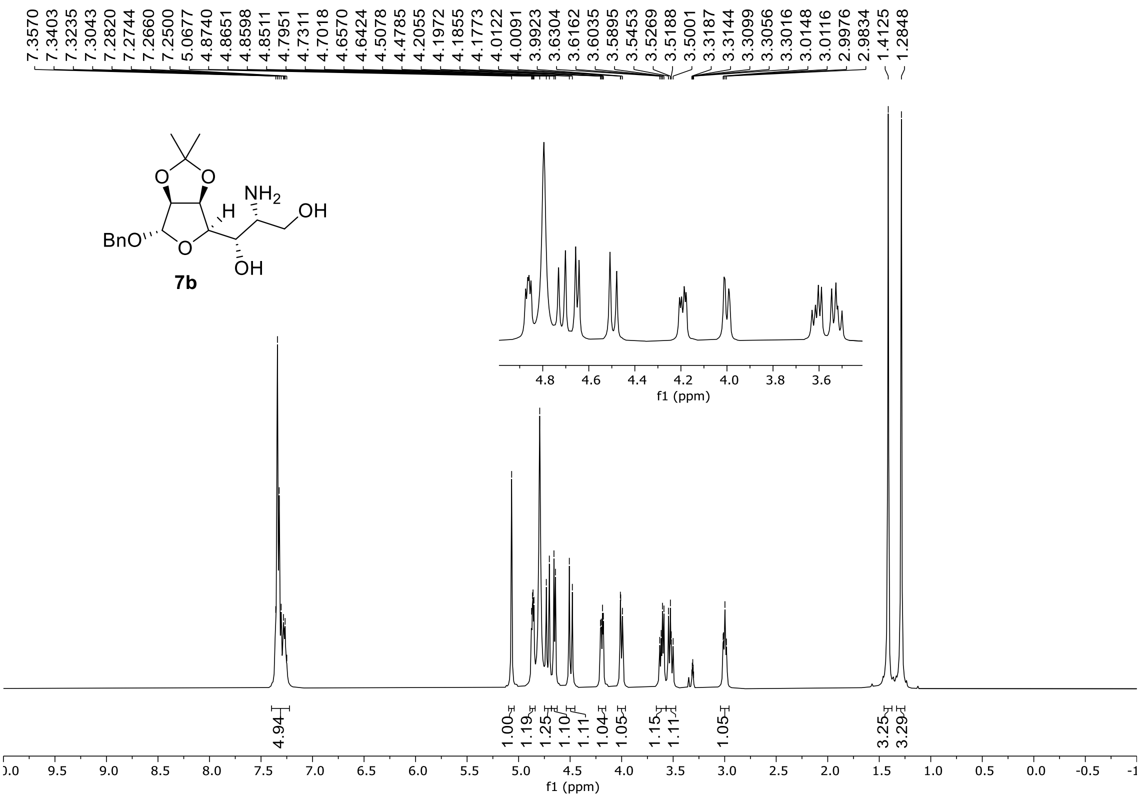

Figure S49. ^{1}H NMR spectrum of compound **7b** (CD_3OD, 400 MHz)

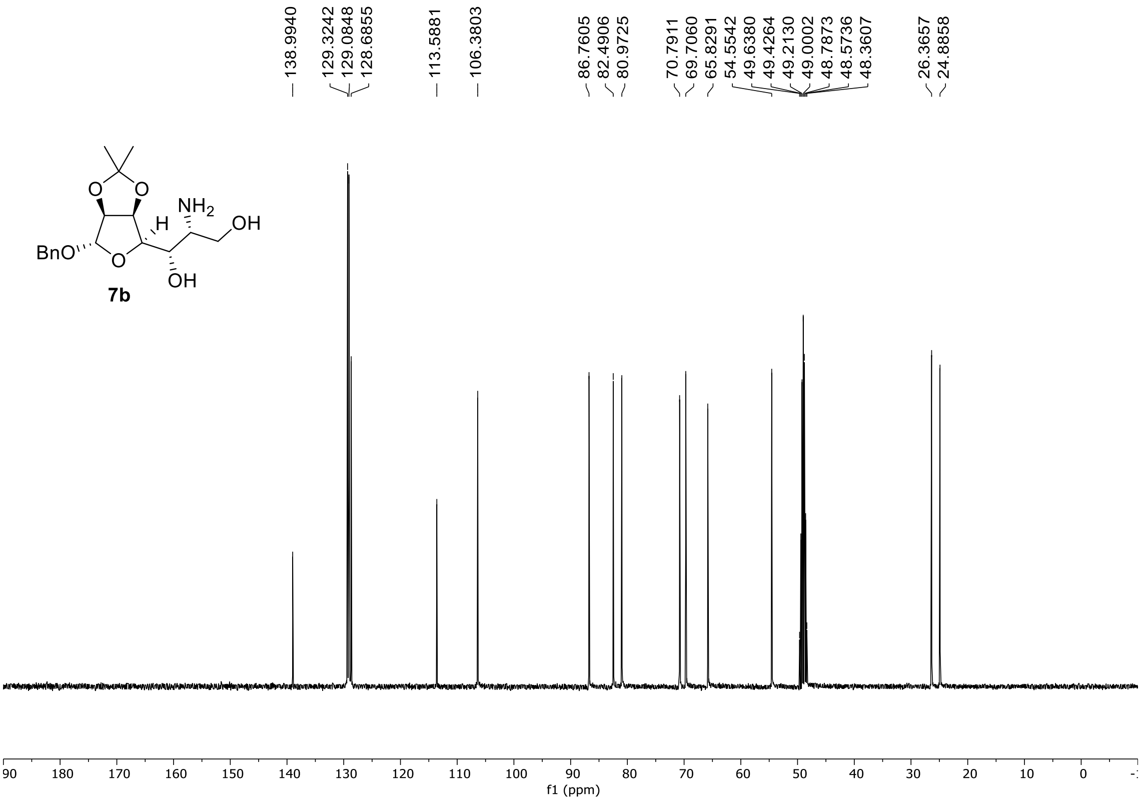

Figure S50. $^{13}C\{^{1}H\}$ NMR spectrum of compound **7b** (CD_3OD, 100 MHz)

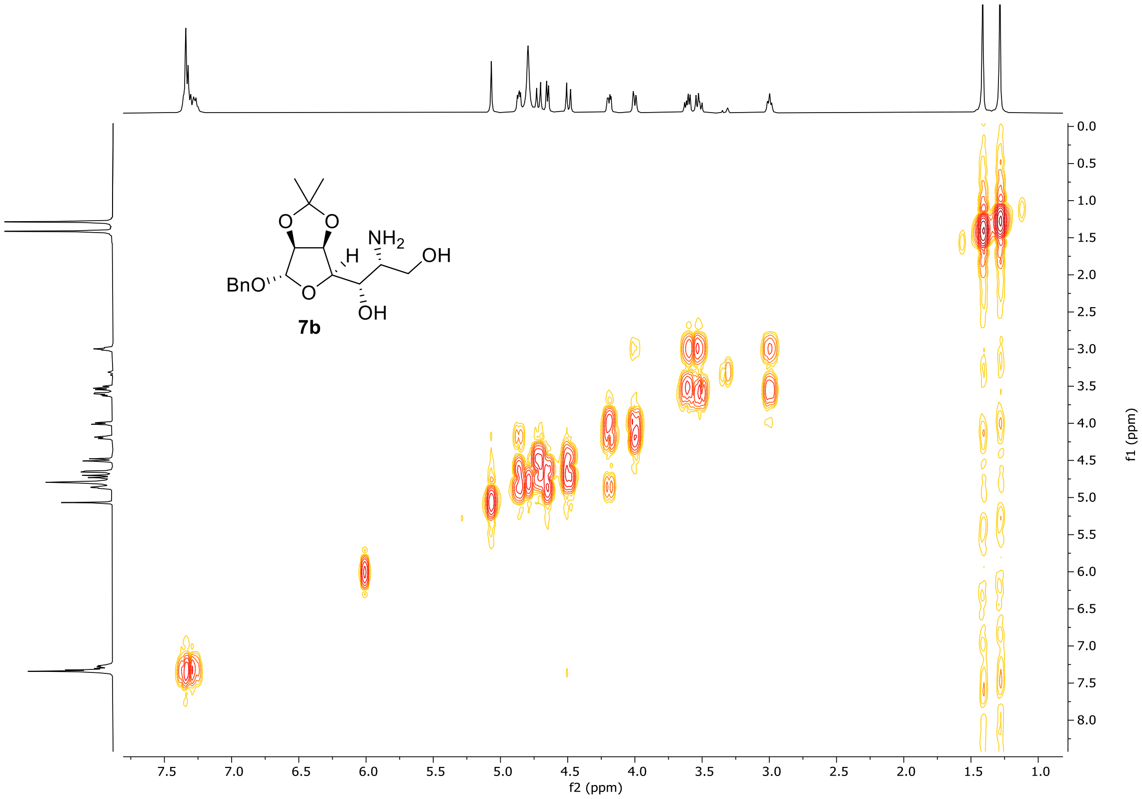

Figure S51. $^{1}H/^{1}H$ gCOSY spectrum of compound **7b** (CD_3OD, 400 MHz)

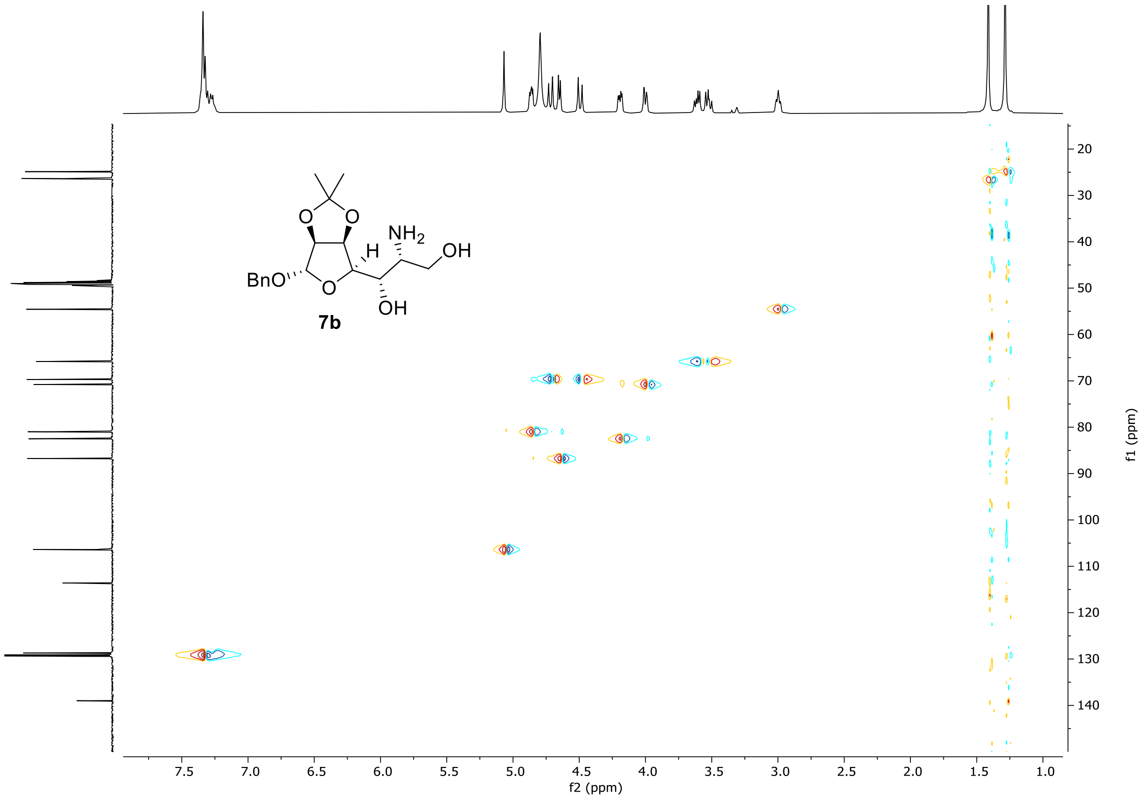

Figure S52. $^{1}H/^{13}C$ gHSQC spectrum of compound **7b** (CD_3OD, 400/100 MHz)

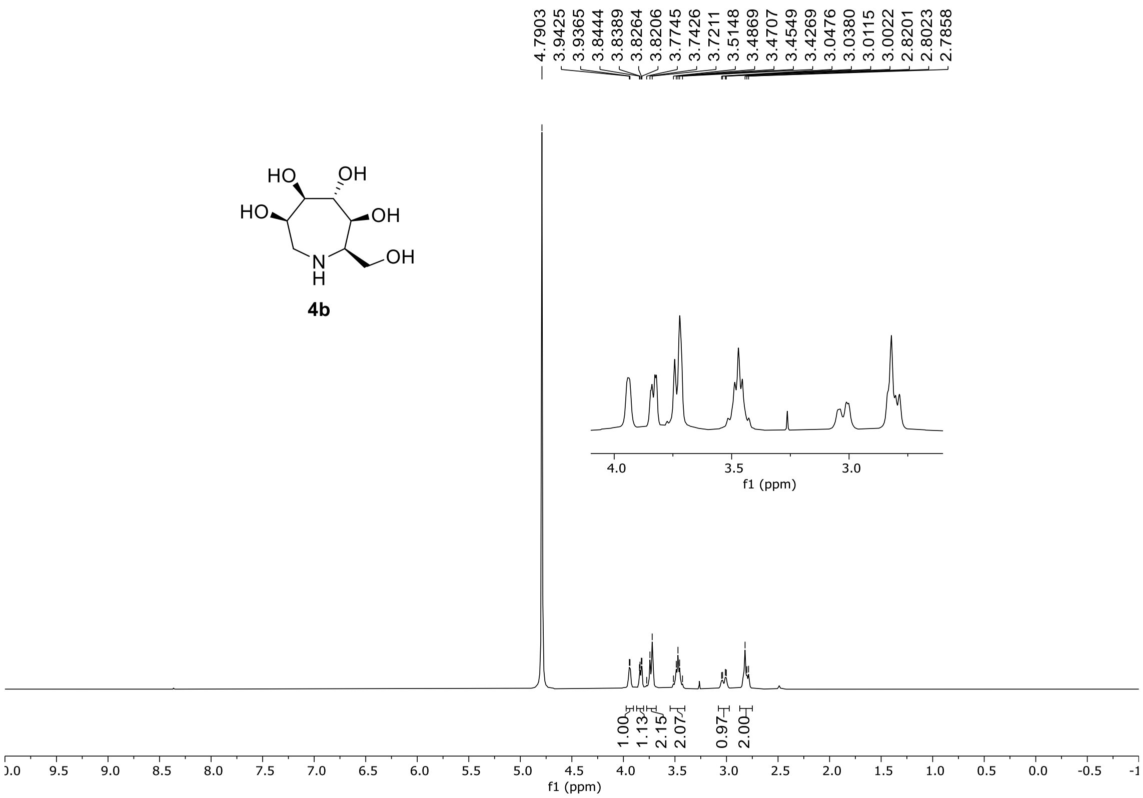

Figure S53. ^{1}H NMR spectrum of compound **4b** (D_2O, 400 MHz)

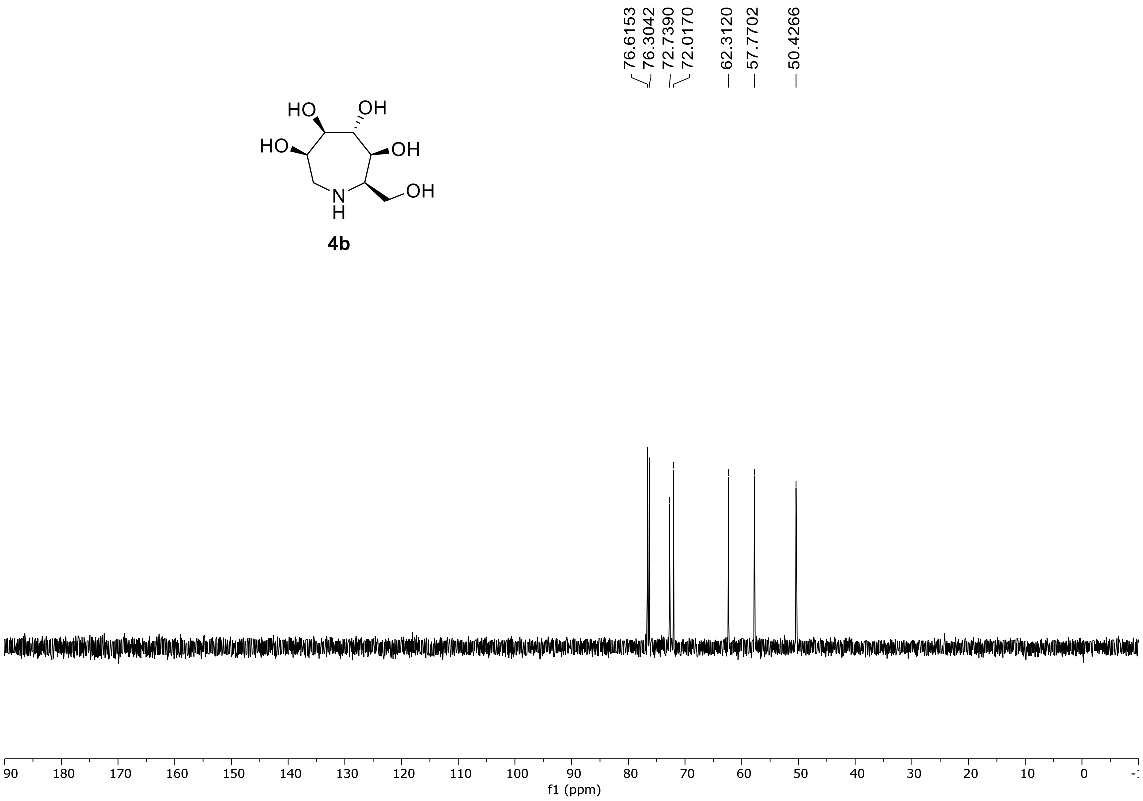

Figure S54. $^{13}C\{^{1}H\}$ NMR spectrum of compound **4b** (D_2O, 100 MHz)

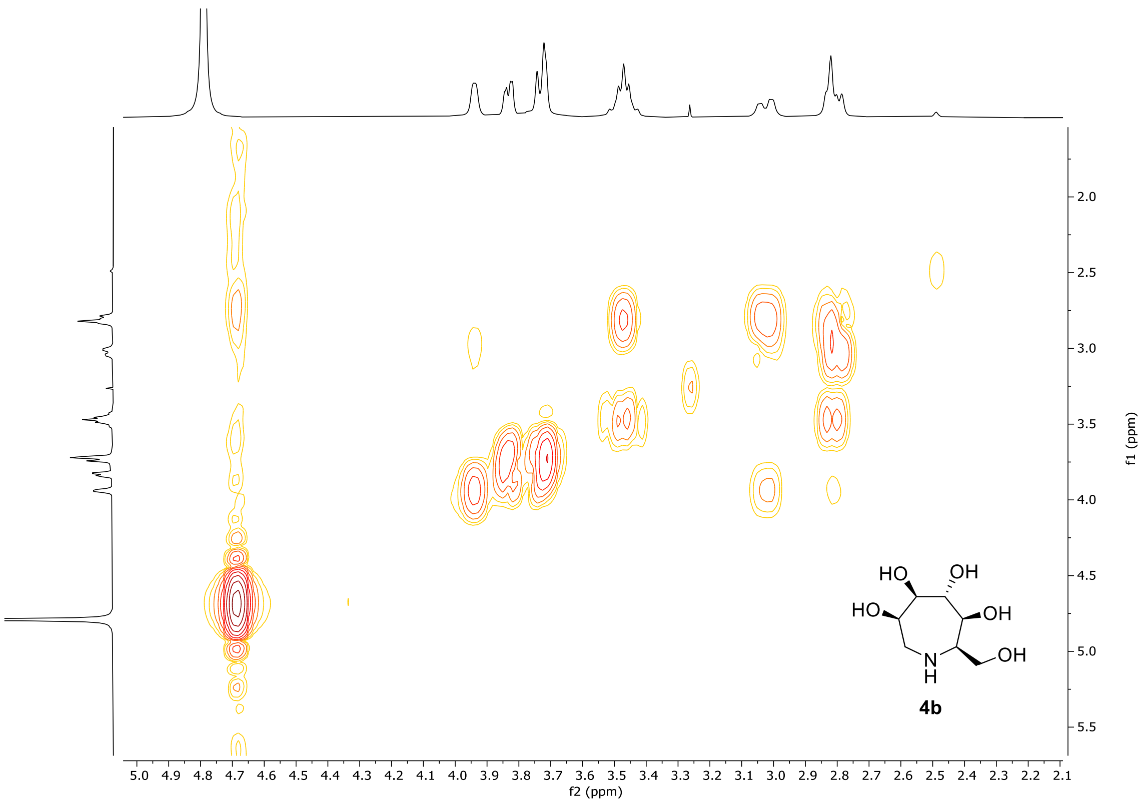

Figure S55. $^1H/^1H$ gCOSY spectrum of compound **4b** (D_2O, 400 MHz)

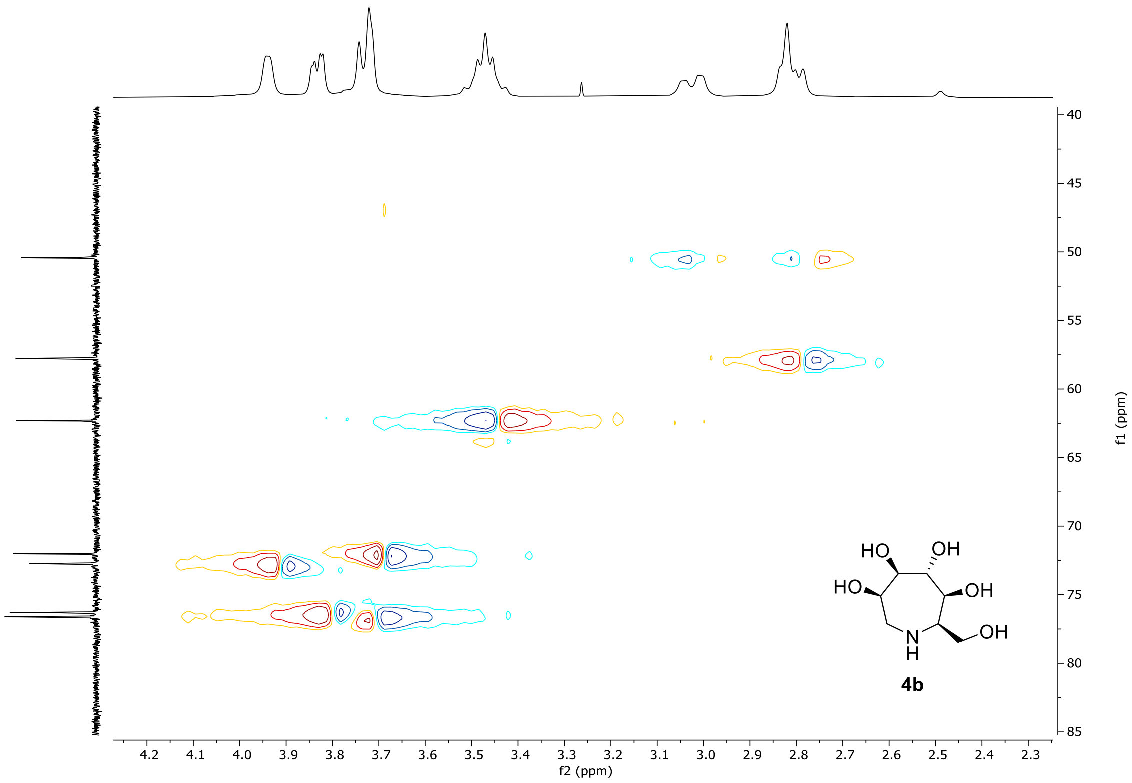

Figure S56. $^1H/^{13}C$ gHSQC spectrum of compound **4b** (D_2O, 400/100 MHz)

3. Data for lysosomal glycosidase inhibition assays

All experiments on biological materials were performed in accordance with the ethical standards of the institutional research committee and with the 1964 Helsinki Declaration and its later amendments. In keeping with ethical guidelines, all blood samples were obtained for storage and analyzed only after written informed consent of the patients (and/or their family members) was obtained, using a form approved by the local Ethics Committee (Assigned code: Lysolate "Late - onset Lysosomal Storage Disorders (LSDs) in the differential diagnosis of neurodegenerative diseases: development of new diagnostic procedures and focus on potential pharmacological chaperones (PCs)." Project ID code: 16774_bio, 5 May 2020, Comitato Etico Regionale per la Sperimentazione Clinica della Regione Toscana, Area Vasta Centro, Florence, Italy). Control samples were anonymized and used only for research purposes. Polyhydroxylated azepane iminosugars **4a**, **4b** and **10a** were screened towards 12 human lysosomal glycosidases at 1 mM in leukocytes (or lymphocytes) isolated from healthy donors (controls). Isolated leukocytes (or lymphocytes) were disrupted by sonication, and a micro-BCA protein assay kit (Sigma–Aldrich) was used to determine the total protein amount for the enzymatic assay, according to the manufacturer instructions.

α-Mannosidase activity was measured in a flat-bottomed 96-well plate. Compound solution (3 µL), 4.29 µg/µL leukocytes homogenate 1:10 (7 µL), and substrate 4-methylumbelliferyl-α-D-mannopyranoside (2.67 mM, 20 µL, Sigma–Aldrich) in Na phosphate/citrate buffer (0.2:0.1, M/M, pH 4.0) containing sodium azide (0.02%) were incubated at 37 °C for 1 h. The reaction was stopped by addition of sodium carbonate (200 µL; 0.5M, pH 10.7) containing Triton X-100 (0.0025 %), and the fluorescence 4-methylumbelliferone released by α-mannosidase activity was measured in SpectraMax M2 microplate reader (λex=365 nm, λem=435 nm; Molecular Devices). Inhibition is given with respect to the control (without compound). Data are mean SD ± (n=3).

β-Mannosidase activity was measured in a flat-bottomed 96-well plate. Compound solution (3 µL), 4.29 µg/µL leukocytes homogenate 1:10 (7 µL), and substrate 4-methylumbelliferyl-β-D-mannopyranoside (1.33 mM, 20 µL, Sigma–Aldrich) in Na phosphate/citrate buffer (0.2:0.1, M/M, pH 4.0) containing sodium azide (0.02%) were incubated at 37 °C for 1h. The reaction was stopped by addition of sodium carbonate (200 µL; 0.5M, pH 10.7) containing Triton X-100 (0.0025 %), and the fluorescence of 4-methylumbelliferone released by β-mannosidase activity was measured in SpectraMax M2 microplate reader (λex=365 nm, λem=435 nm; Molecular Devices). Inhibition is given with respect to the control (without compound). Data are mean SD ± (n=3).

α-Galactosidase activity was measured in a flat-bottomed 96-well plate. Compound solution (3 µL), 4.29 µg/µL leukocytes homogenate 1:3 (7 µL), and substrate 4-methylumbelliferyl α-D-galactopyranoside (1.47 mM, 20 µL, Sigma–Aldrich) in acetate buffer (0.1 M, pH 4.5) containing sodium azide (0.02%) were incubated at 37 °C for 1 h. The reaction was stopped by addition of sodium carbonate (200 µL; 0.5M, pH 10.7) containing Triton X-100

(0.0025 %), and the fluorescence 4-methylumbelliferone released by α-galactosidase activity was measured in SpectraMax M2 microplate reader (λex=365 nm, λem=435 nm; Molecular Devices). Inhibition is given with respect to the control (without compound). Data are mean SD ± (n=3).

β-Galactosidase activity was measured in a flat-bottomed 96-well plate. Compound solution (3 µL), 4.29 µg/µL leukocytes homogenate 1:10 (7 µL), and substrate 4-methylumbelliferyl β-D-galactopyranoside (1.47 mM, 20 µL, Sigma–Aldrich) in acetate buffer (0.1 M, pH 4.3) containing NaCl (0.1M) and sodium azide (0.02%) were incubated at 37 °C for 1 h. The reaction was stopped by addition of sodium carbonate (200 µL; 0.5M, pH 10.7) containing Triton X-100 (0.0025 %), and the fluorescence 4-methylumbelliferone released by β-galactosidase activity was measured in SpectraMax M2 microplate reader (λex=365 nm, λem=435 nm; Molecular Devices). Inhibition is given with respect to the control (without compound). Data are mean SD ± (n=3).

α-Glucosidase activity was measured in a flat–bottomed 96 well plate. Compound solution (3 µL), 4.29 µg/µL lymphocytes homogenate (7 µL) and 20 µL of substrate solution of 4-methylumbelliferyl-α-D-glucopyranoside (Sigma-Aldrich) in Na acetate buffer (0.2 M, pH 4.0) containing sodium azide (0.02%) were incubated for 1 h at 37 °C. The reaction was stopped by the addition of a solution of sodium carbonate (200 µL; 0.5M, pH 10.7) containing Triton X-100 (0.0025 %), and the fluorescence of 4-methylumbelliferone released by α-glucosidase activity was measured in SpectraMax M2 microplate reader (λex=365 nm, λem=435 nm; Molecular Devices). Inhibition is given with respect to the control (without compound). Data are mean SD ± (n=3).

β-Glucosidase activity was measured in a flat-bottomed 96-well plate. Compound solution (3 µL), 4.29 µg/µL leukocytes homogenate (7 µL), and substrate 4-methylumbelliferyl-β-D-glucopyranoside (3.33 mM, 20 µL, Sigma–Aldrich) in citrate/phosphate buffer (0.1:0.2, M/M, pH 5.8) containing sodium taurocholate (0.3%), Triton X-100 (0.15%) and sodium azide (0.02%) were incubated for 1 h at 37 °C. The reaction was stopped by addition of sodium carbonate (200 µL; 0.5M, pH 10.7) containing Triton X-100 (0.0025 %), and the fluorescence of 4-methylumbelliferone released by β-glucosidase activity was measured in SpectraMax M2 microplate reader (λex=365 nm, λem=435 nm; Molecular Devices). Inhibition is given with respect to the control (without compound). Data are mean SD ± (n=3).

α-*N*-Acetylgalactosaminidase (α-NAGA) activity was measured in a flat-bottomed 96-well plate. Compound solution (3 µL), 4.29 µg/µL leukocytes homogenate 1:2 (7 µL), and substrate 4-methylumbelliferyl-α-*N*-acetylgalactosaminide (1.0 mM, 20 µL, Sigma–Aldrich) in Na-phosphate/citrate buffer (0.2:0.1, M/M, pH 4.7) containing sodium azide (0.02%) were incubated for 1 h at 37 °C. The reaction was stopped by addition of sodium carbonate (200 µL; 0.5M, pH 10.7) containing Triton X-100 (0.0025 %), and the fluorescence of 4-methylumbelliferone released by α-NAGA activity was measured in SpectraMax M2 microplate

reader (λex=365 nm, λem=435 nm; Molecular Devices). Inhibition is given with respect to the control (without compound). Data are mean SD ± (n=3).

α-Iduronidase activity was measured in a flat-bottomed 96-well plate. Compound solution (3 µL), 4.29 µg/µL leukocytes homogenate 1:3 (7 µL), and substrate 4-methylumbelliferyl- α-L-iduronide (1.58 mM, 20 µL, Sigma–Aldrich) in sodium formate buffer, 0.1 M, pH 3.5 containing NaCl (37.5mM) and sodium azide (0.02%) were incubated for 1 h at 37 °C. The reaction was stopped by addition of sodium carbonate (200 µL; 0.5M, pH 10.7) containing Triton X-100 (0.0025 %), and the fluorescence of 4-methylumbelliferone released by α-iduronidase activity was measured in SpectraMax M2 microplate reader (λex=365 nm, λem=435 nm; Molecular Devices). Inhibition is given with respect to the control (without compound). Data are mean SD ± (n=3).

α-Fucosidase activity was measured in a flat-bottomed 96-well plate. Compound solution (3 µL), 4.29 µg/µL leukocytes homogenate 1:3 (7 µL), and substrate 4-methylumbelliferyl α-L-fucopyranoside (1.51 mM, 20 µL, Sigma–Aldrich) in Na phosphate/citrate buffer (0.2:0.1, M/M, pH 5.5) containing sodium azide (0.02%) were incubated at 37 °C for 1 h. The reaction was stopped by addition of sodium carbonate (200 µL; 0.5M, pH 10.7) containing Triton X-100 (0.0025 %), and the fluorescence 4-methylumbelliferone released by α- fucosidase activity was measured in SpectraMax M2 microplate reader (λex=365 nm, λem=435 nm; Molecular Devices). Inhibition is given with respect to the control (without compound). Data are mean SD ± (n=3).

Galactosamine (*N*-acetyl)-6-sulfatase (GALNS) activity was measured by setting the reaction in 0.2 ml tubes and performing the experiments in triplicates as follows. Compound solution (3 µl), 4.29 µg/µL leukocytes homogenate 1:5 (7 µL) and 20 µl of 4-methylumbelliferyl-β-galactoside-6-sulphate•Na substrate solution in Na-Acetate/acetic acid buffer (0.1M/0.1 M, pH 4.3) containing 0.1 M NaCl , sodium azide (0.02%) and 5 mM Pb-acetate were incubated for 17 h at 37 °C. After step 1 the tubes were placed on an ice cooler and the reaction was stopped by addition of 5 µl of Na-phosphate buffer (0.9 M, pH 4.3) containing sodium azide (0.02%) and by efficient mixing with vortex. Then, 10 µl of β-Gal-A-10U were added to each sample and samples were incubated for 2 h at 37°C. At the end of this period the tubes were placed on an ice cooler and the samples were transferred in a cooled flat–bottomed 96 well plate and the reaction was immediately stopped by addition of sodium carbonate (200 µL; 0.5M, pH 10.7) containing Triton X-100 (0.0025 %), and fluorescence of 4-methylumbelliferone released by GALNS activity was measured in SpectraMax M2 microplate reader (λex=365 nm, λem=435 nm; Molecular Devices). Inhibition is given with respect to the control (without compound). Data are mean SD ± (n=3).

β-Hexosaminidase A (Hex A) activity was measured in a flat-bottomed 96-well plate. Compound solution (3 µL), 4.29 µg/µL leukocytes homogenate 1:10 (7 µL), and substrate 4-Methylumbelliferyl-β-D-*N*-acetylglucosamine-6-sulfate (1.0 mM, 20 µL, Sigma–Aldrich) in Na phosphate/citrate buffer (0.2:0.1, M/M, pH 4.4) containing sodium azide (0.02%) were incubated for 1 h at 37 °C. The reaction was stopped by addition of sodium carbonate (200 µL; 0.5M, pH

10.7) containing Triton X-100 (0.0025 %), and the fluorescence of 4-methylumbelliferone released by Hex A activity was measured in SpectraMax M2 microplate reader (λex=365 nm, λem=435 nm; Molecular Devices). Inhibition is given with respect to the control (without compound). Data are mean SD + (n=3).

β-Hexosaminidases (Hex A + Hex B) activity was measured in a flat-bottomed 96-well plate. Compound solution (3 µL), 4.29 µg/µL leukocytes homogenate 1:10 (7 µL), and substrate 4-Methylumbelliferyl-*N*-acetyl-β-D-glucosaminide (5.0 mM, 20 µL, Sigma–Aldrich) in Na phosphate/citrate buffer (0.2:0.1, M/M, pH 4.4) containing sodium azide (0.02%) were incubated for 1 h at 37 °C. The reaction was stopped by addition of sodium carbonate (200 µL; 0.5M, pH 10.7) containing Triton X-100 (0.0025 %), and the fluorescence of 4-methylumbelliferone released by β-Hexosaminidases activity was measured in SpectraMax M2 microplate reader (λex=365 nm, λem=435 nm; Molecular Devices). Inhibition is given with respect to the control (without compound). Data are mean SD + (n=3).

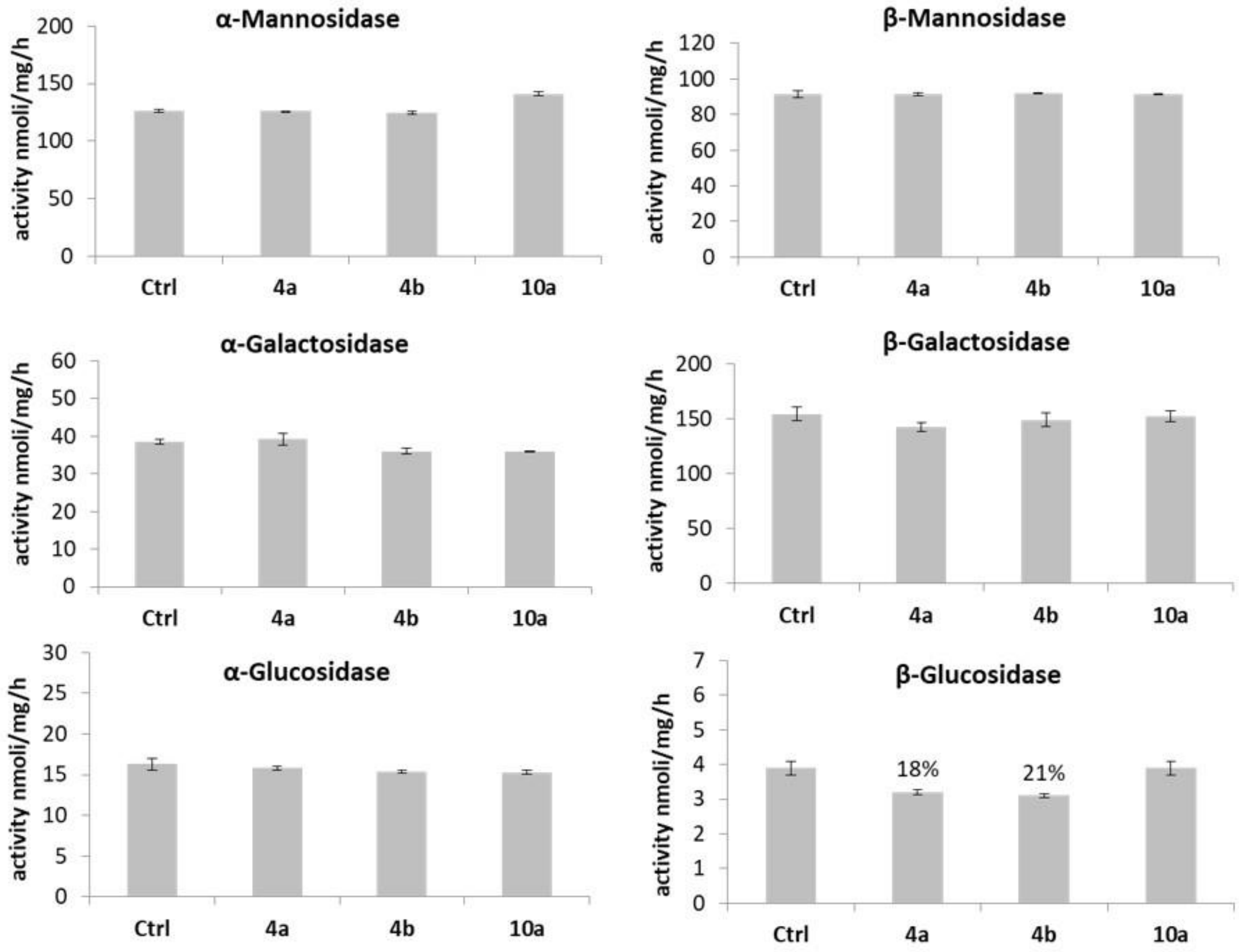

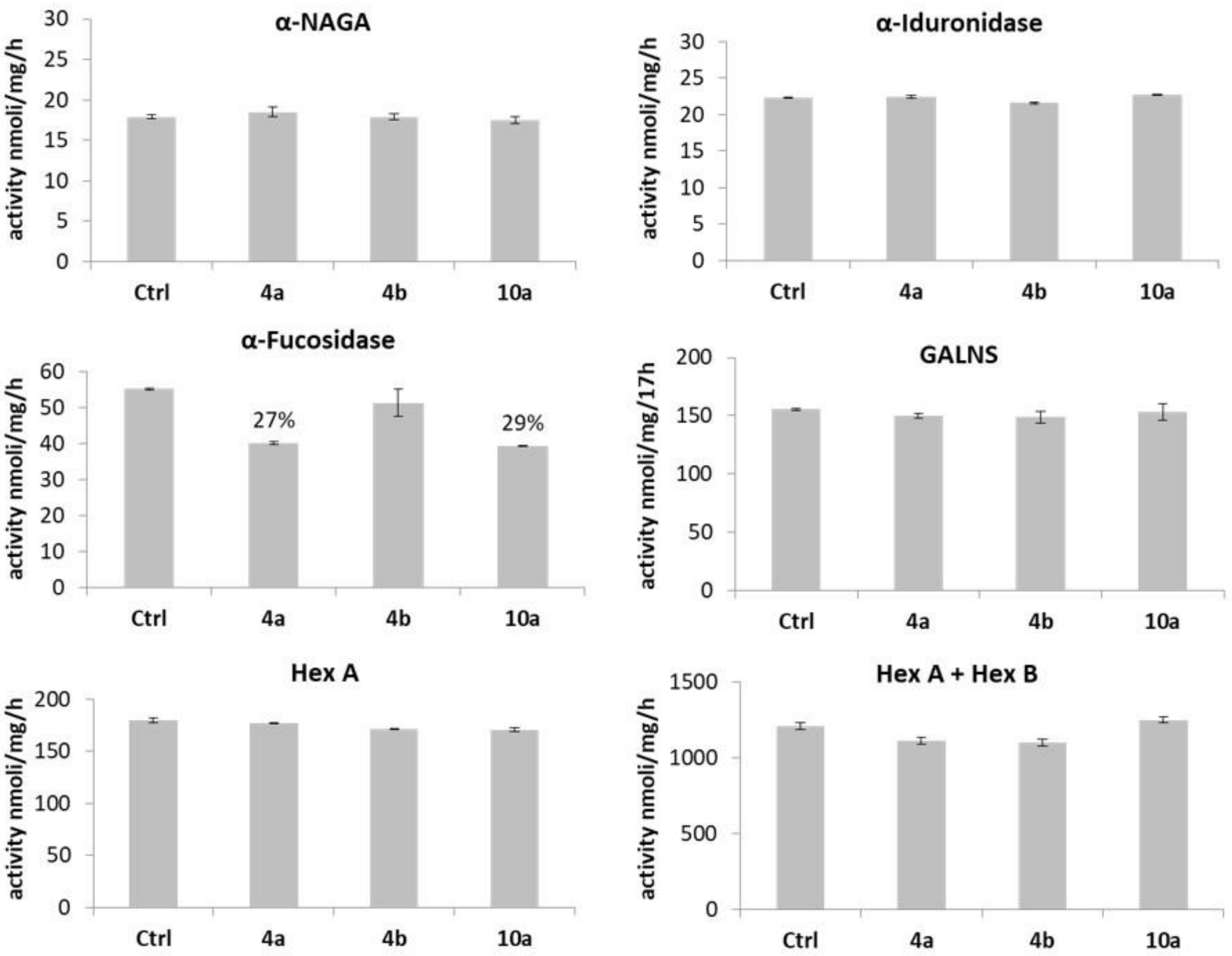

Figure S57. Activity of 12 lysosomal glycosidases in an extract from human leukocytes or lymphocytes isolated from healthy donors in the presence of polyhydroxylated azepane iminosugars **4a**, **4b** and **10a** at 1 mM. The corresponding calculated percentage of inhibition is reported above each bar (when the percentage is $\geq$10%)

4. Crystal structure determination for compounds 2b, 7a and 8a

X-Ray Data Collection

Single crystals were mounted in a loop and intensity data collected at 100° K with a Bruker Apex-II CCD diffractometer, using a Cu–Kα (λ = 1.54184 Å) radiation.

Data were collected with the Bruker APEX2 program, integrated, and reduced with the Bruker SAINT software.

The integrated intensities, measured using the φ and ω scan mode, were corrected for Lorentz and polarization effects.

The substantial redundancy in data allows empirical absorption corrections to be applied using SADABS-2016/2 (Bruker AXS area detector scaling and absorption correction).[4]

Structures were solved by direct methods of SIR2019[5] and refined using the full-matrix least squares on F^2 provided by SHELXL-2014/6.[6]

The non-hydrogen atoms were refined anisotropically whereas hydrogen atoms as isotropic.

Copies of the data can be obtained, free of charge, from CCDC, 12 Union Road, Cambridge, CB2 1EZ UK (e-mail: deposit@ccdc.cam.ac.uk; internet://www.ccdc.cam.ac.uk) with the deposition numbers reported below for each compound.

Crystallographic data for compound 2b:

Molecular formula $C_{17}H_{22}O_5$, M=306.34, Monoclinic, space group P 2_1, a=12.1271(7), b=5.2663(3), c=12.7831(7)Å, β=91.601(2)

V=816.1(1)$Å^3$, Z=2 D_c=1.247, μ=0.751 mm^{-1}, F (000) =328.

7945 reflections were collected with a 3.646<θ<68.464 range with a completeness to theta 97,9%; 2884 were independent, the parameters were 199 and the final R index was 0.625 for reflections having I>2σI.

Hydrogen atoms were all assigned in calculated positions.

Deposition number at the Cambridge Crystallographic Data Centre: **CCDC 2265730**.

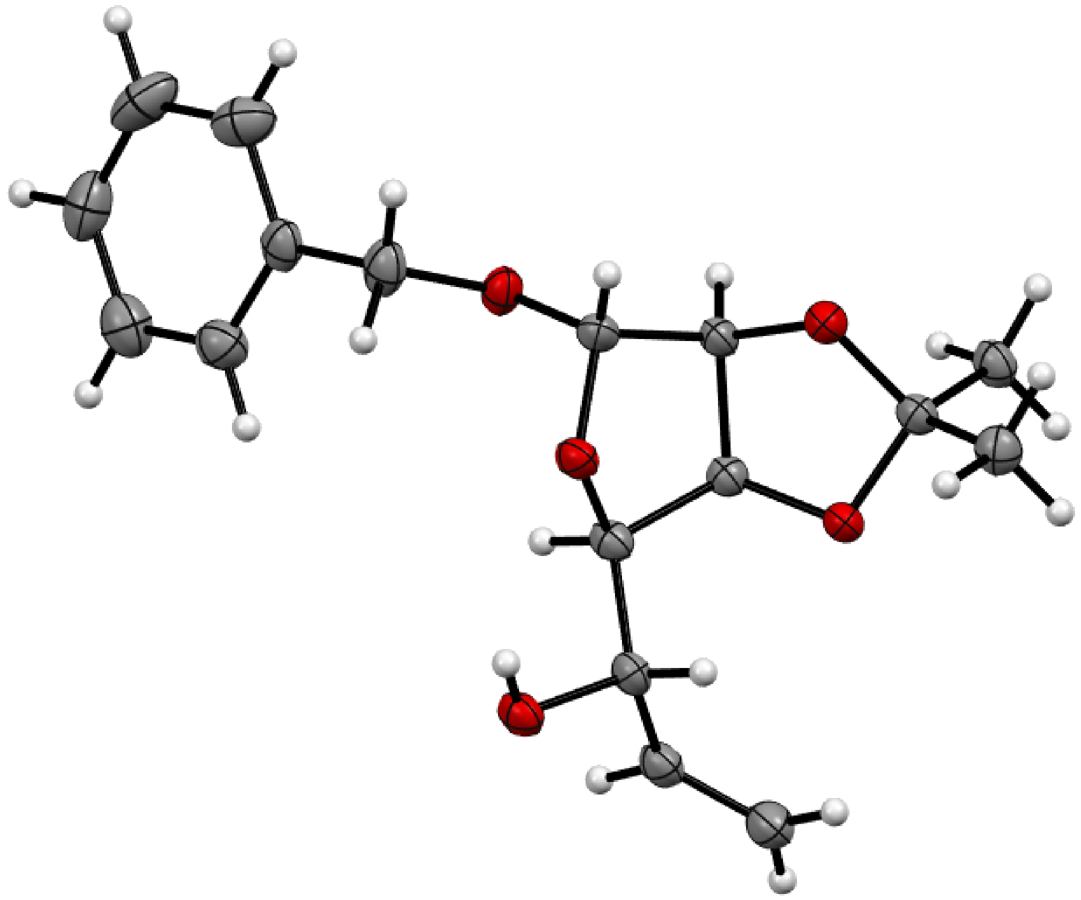

Figure S58. Thermal ellipsoid plot (30% probability level) for compound **2b**, **CCDC 2265730**

Crystallographic data for compound 7a:

Asymmetric unit contains two independent molecules.

Molecular formula 2x($C_{17}H_{25}NO_6$), M=2x339.38, monoclinic, space group P 2_1, a=6.2256(2), b=30.195(1), c=9.4572(3)Å, β=93.891(2) V=1773.7(1)$Å^3$, Z=2 D_c=1.271, μ=0.799 mm^{-1}, F (000) =728.

24634 reflections were collected with a 2.927<θ<68.119 range with a completeness to theta 99,8%; 6423 were independent, the parameters were 449 and the final R index was 0.0436 for reflections having I>2σI.

Hydrogen atoms were all assigned in calculated positions except for H on N1 (A+B) found in FD map.

Deposition number at the Cambridge Crystallographic Data Centre: **CCDC 2265731**.

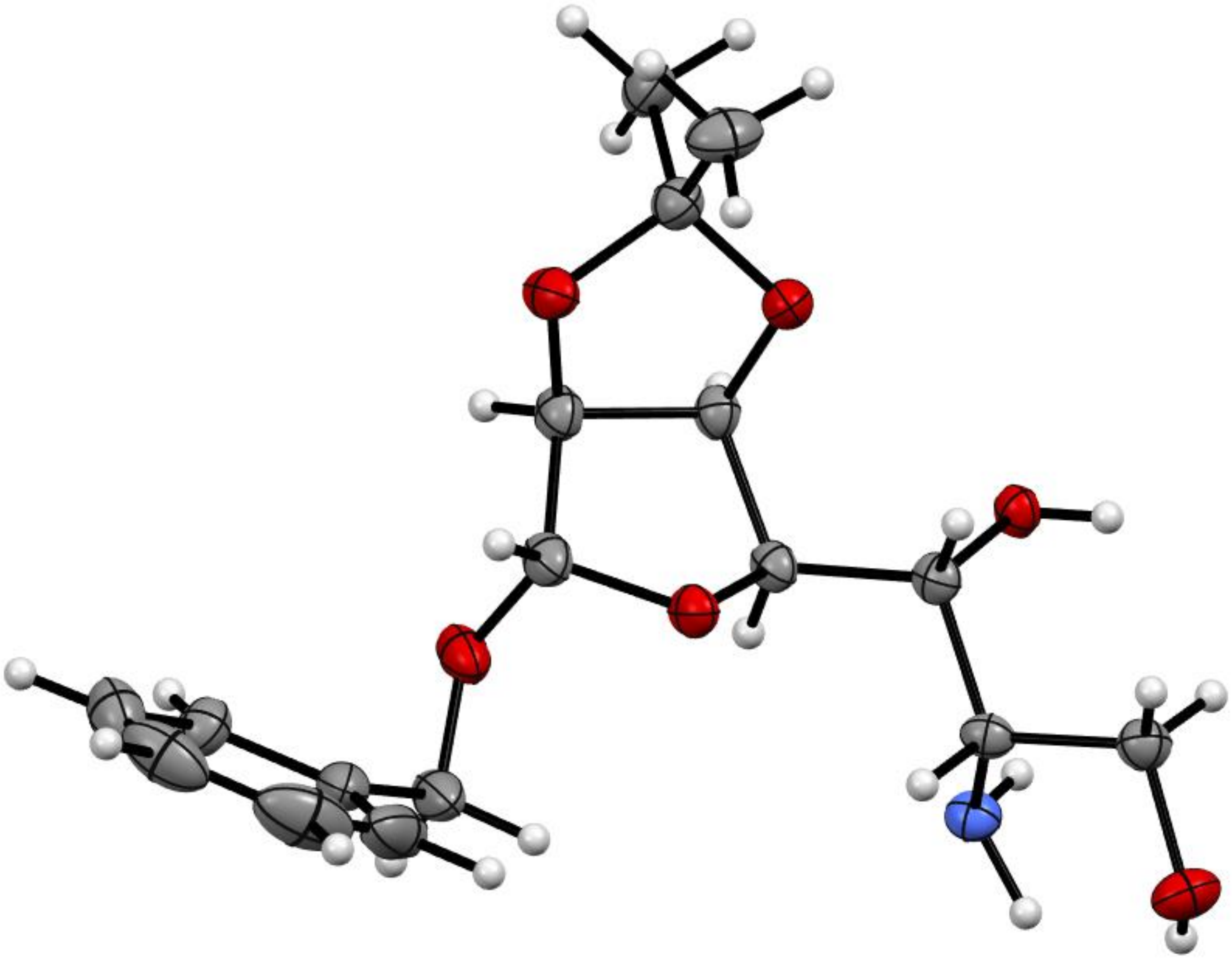

Figure S59. Thermal ellipsoid plot (30% probability level) for compound **7a**, **CCDC 2265731**

Crystallographic data for compound 8a:

Molecular formula $C_{10}H_{17}NO_5$, M=231.24, Orthorombic, space group P 2_1 2_1 2_1, a=6.2950(7), b=6.7148(7), c=25.2580(6)Å,

V=1067.6(1)$Å^3$, Z=4 D_c=1.439, μ=0.975 mm^{-1}, F (000) =496.

4142 reflections were collected with a $7.864<\theta<74.546$ range with a completeness to theta 94,2%; 1883 were independent, the parameters were 149 and the final R index was 0.0467 for reflections having $I>2\sigma I$.

Hydrogen atoms were all assigned in calculated positions, except for H on N1 found in FD map.

Two intermolecular hydrogen bonds are significant and listed below.

O1 -- H1<br>0.820 Å	O1 -- O3 (1)<br>2.763 Å	H1 – O3 (1)<br>1.955 Å	O1 – H1 – O3 (1)<br>168.31°
O3 – H3<br>0.820 Å	O3 – N1 (2)<br>2.790 Å	H3 – N1 (2)<br>2.037 Å	O3 – H3 – N1 (2)<br>152.41°

The relative symmetry operations are: (1) x, y+1, z
(2) x-1/2, -y+1/2, -z+2

Deposition number at the Cambridge Crystallographic Data Centre: **CCDC 2265732**.

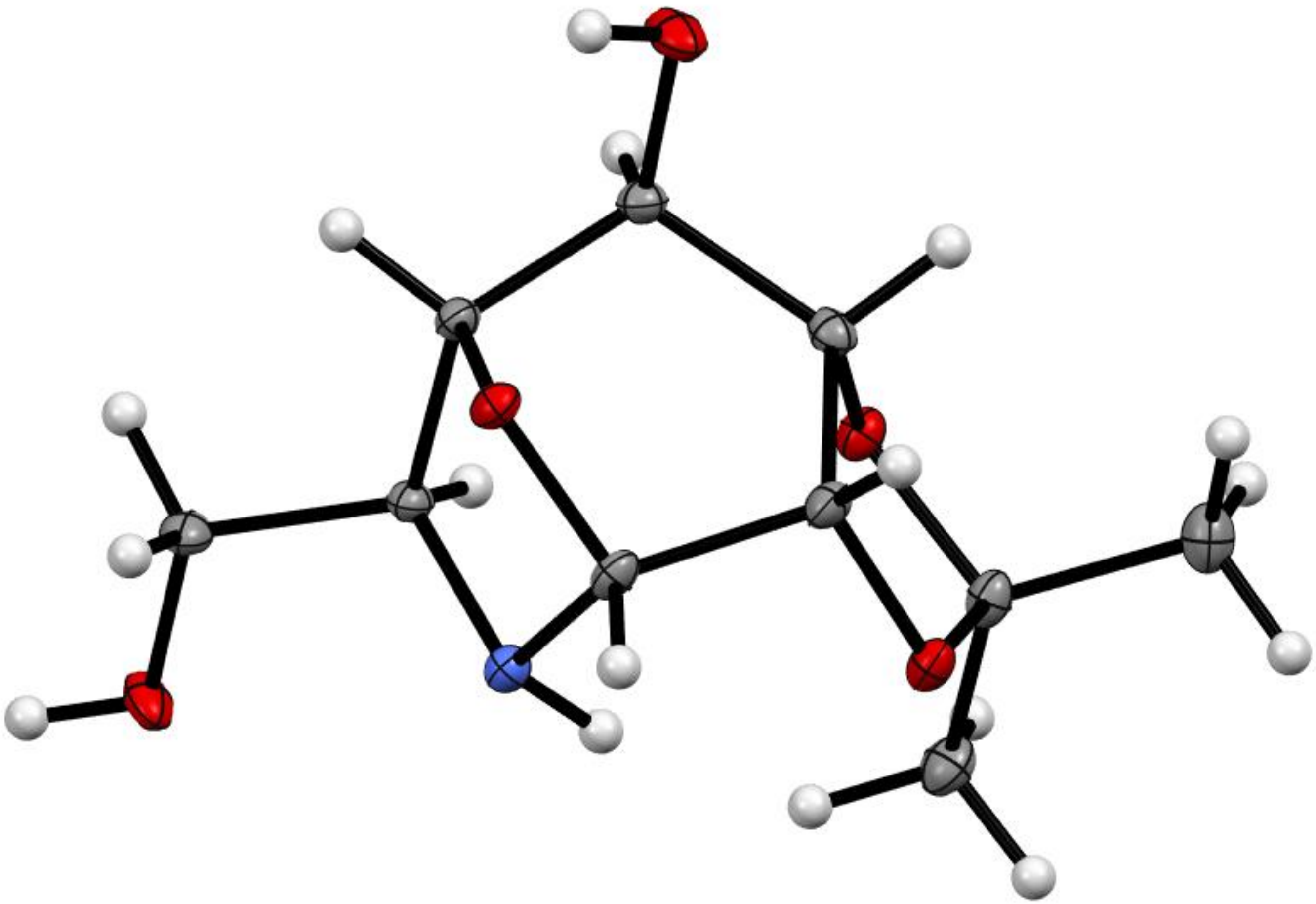

Figure S60. Thermal ellipsoid plot (30% probability level) for compound **8a**, **CCDC 2265732**

5. References

1. Matassini, C.; Mirabella, S.; Goti, A.; Cardona, F., Double Reductive Amination and Selective Strecker Reaction of a D-Lyxaric Aldehyde: Synthesis of Diversely Functionalized 3,4,5-Trihydroxypiperidines. *Eur. J. Org. Chem.* **2012,** *2012*, 3920-3924.

2. Mirabella, S.; Fibbi, G.; Matassini, C.; Faggi, C.; Goti, A.; Cardona, F., Accessing 2-substituted piperidine iminosugars by organometallic addition/intramolecular reductive amination: aldehyde *vs*. nitrone route. *Org. Biomol. Chem.* **2017,** *15*, 9121-9126.

3. Estévez, A. M.; Soengas, R. G.; Otero, J. M.; Estévez, J. C.; Nash, R. J.; Estévez, R. J., Studies on the transformation of nitrosugars into iminosugars III: synthesis of (2*R*,3*R*,4*R*,5*R*,6*R*)-2-(hydroxymethyl)azepane-3,4,5,6-tetraol and (2*R*,3*R*,4*R*,5*R*,6*S*)-2-(hydroxymethyl)azepane-3,4,5,6-tetraol. *Tetrahedron: Asymmetry* **2010,** *21*, 21-26.

4. Krause, L.; Herbst-Irmer, R.; Sheldrick G.M.; Stalke D., J., Comparison of silver and molybdenum microfocus X-ray sources for single-crystal structure determination. *Appl. Cryst.* **2015**, *48*, 3-10.

5. Burla, M.C.; Caliandro R.; Carrozzini B.; Cascarano, G. L.; Cuocci, C.; Giacovazzo, C.; Mallamo, M.; Mazzone A.; Polidori G., *Crystal structure determination and refinement via SIR2014. J. Appl. Cryst.* **2015**, *48*, 306–309.

6. Sheldrick, G. M., Crystal structure refinement with SHELXL. *Acta Cryst.* **2015**, *C71*, 3-8.
